# Advancing open-source visual analytics in digital pathology: A systematic review of tools, trends, and clinical applications

**DOI:** 10.1016/j.jpi.2025.100454

**Published:** 2025-05-23

**Authors:** Zahoor Ahmad, Mahmood Alzubaidi, Khaled Al-Thelaya, Corrado Calí, Sabri Boughorbel, Jens Schneider, Marco Agus

**Affiliations:** a College of Science and Engineering, Hamad Bin Khalifa University, Qatar Foundation, 34110 Doha, Qatar; b Neuroscience Institute Cavalieri Ottolenghi, University of Turin, Orbassano, Italy; c Department of Neuroscience ‘‘Rita Levi Montalcini’’, University of Turin, Turin, Italy; d Qatar Computing Research Institute, Hamad Bin Khalifa University, Qatar Foundation, 34110 Doha, Qatar

**Keywords:** Open-source, Visual analytics, Digital pathology, Histopathology, Computational pathology, Whole slide imaging, Machine learning in pathology, Cancer research, Image analysis, Clinical implementation

## Abstract

Histopathology is critical for disease diagnosis, and digital pathology has transformed traditional workflows by digitizing slides, enabling remote consultations, and enhancing analysis through computational methods. In this systematic review, we evaluated open-source visual analytics abilities in digital pathology by screening 254 studies and including 52 that met predefined criteria. Our analysis reveals that these solutions—comprising abilities (*n* = 29), software (*n* = 13), and frameworks (*n* = 10)—are predominantly applied in cancer research (e.g., breast, colon, ovarian, and prostate cancers) and primarily utilize whole slide images. Key contributions include advanced image analysis capabilities (as demonstrated by platforms such as QuPath and CellProfiler) and the integration of machine learning for diagnostic support, treatment planning, automated tissue segmentation, and collaborative research. Despite these promising advancements, challenges such as high computational demands, limited external validation, and difficulties integrating into clinical workflows remain. Future research should focus on establishing standardized validation frameworks, aligning with regulatory requirements, and enhancing user-centric designs to promote robust, interoperable solutions for clinical adoption.

## Introduction

Histopathology is a key part of medical diagnosis, involves studying disease manifestations through microscopic tissue examination.^1^ It plays a critical role in medicine, particularly for cancer detection, classification, and staging.^2^ Traditionally, histopathologists examine thin, stained (e.g., hematoxylin and eosin, H&E) tissue sections on glass slides under a microscope,^3^ requiring extensive training to identify subtle pathological changes in cell morphology, tissue architecture, and specific markers.^4^ Beyond diagnosis, histopathology informs treatment decisions, provides prognostic information, and aids in monitoring disease progression and therapy response.^4^ The field has continually evolved, integrating immunohistochemistry (IHC), molecular pathology, and, more recently, digital imaging for increasingly powerful disease characterization.^5^

Histopathology is undergoing a significant transformation with the advent of digital pathology (DP), shifting traditional microscopy towards digital systems.^6^ Whole slide imaging (WSI) is fundamental to this shift, representing a necessary first step for integrating diverse digital tools.^7^ WSI systems digitize entire glass slides using scanners with complex optics and cameras, creating high-resolution gigapixel images.^1^^,^^3^^,^^7^^,^^8^ These “virtual slides” allow pathologists to view and interact with tissue images on computer monitors, akin to navigating online maps.^7^ The process involves sophisticated scanning techniques (e.g., tile-based, line-scanning) and image stitching, with file sizes often necessitating compression (e.g., JPEG 2000) and multi-resolution pyramid storage formats for efficient viewing and navigation.^7^ This digitization enables numerous benefits: robust digital slide archival, remote consultation and telepathology (facilitating subspecialty access and international collaboration), enhanced multi-disciplinary tumor board presentations, standardized educational materials, and powerful platforms for research, including quantitative biomarker analysis.^1^^,^^2^^,^^7^ Crucially, studies have shown that diagnostic interpretations using WSI can be equivalent to traditional light microscopy for specific applications, such as breast needle biopsies.^9^ DP thus opens the door for computer-aided diagnosis, applying artificial intelligence (AI) to assist with tasks like automated region detection and quantitative analysis, potentially improving diagnostic efficiency and consistency.^4^^,^^5^^,^^7^^,^^8^ Visual analytics, which synthesizes automated analysis derived from AI and machine learning (ML) with interactive visualizations, is particularly suited to supporting reasoning on the large, complex datasets generated in DP.^10^ AI, particularly deep learning (DL) using architectures like Convolutional Neural Networks (CNNs), excels at extracting complex features from raw image data, mirroring capabilities seen across medical imaging and beyond.^11^ Visual analytic approaches leverage these AI/ML capabilities (e.g., for classification or segmentation) and present the results interactively, enabling pathologists to explore and interpret the vast information within WSIs.^11^^,^^12^ These tools aim to enhance pathologists' abilities by highlighting salient information, facilitating navigation, providing quantitative measurements, and allowing integration with other data sources (e.g., genomic, clinical) for comprehensive disease analysis.^13^, ^14^, ^15^

Significant interest has focused recently on developing visual analytics techniques and AI applications for DP workflows. AI algorithms are being developed for diverse tasks, including screening slides, automated cancer grading (e.g., Gleason grading in prostate cancer), identifying prognostically significant features like perineural invasion, and even performing *in silico* staining.^11^^,^^16^ Various studies propose interactive visual analytics methods to support WSI analysis, including systems for tissue microarray study, visualizing multi-variate features, and spatial overviews.^14^^,^^15^^,^^17^ Despite these advancements and the potential demonstrated by AI tools (some achieving regulatory approval for specific tasks like prostate pathology assistance^16^), widespread adoption of DP for routine clinical diagnosis remains limited.^18^ A recent systematic review found only 4% of DP publications focused on routine diagnostic applications, with most covering technical development, AI research, or education.^18^ Many proposed solutions remain primarily research-focused, facing significant hurdles to clinical implementation.^5^^,^^10^^,^^18^ Key challenges include the technical complexity of WSI systems, the need for robust IT infrastructure (high-capacity networks, storage solutions, and high-quality calibrated monitors), substantial initial investment and unclear reimbursement pathways, lack of familiarity among staff requiring extensive training, ensuring data security and privacy, seamless integration with existing laboratory information systems (LIS), and the critical need for rigorous validation and adherence to regulatory requirements (though pathways like the FDA's Class II designation for WSI are simplifying this).^7^^,^^18^, ^19^, ^20^, ^21^, ^22^ Slower adoption is also noted in specific areas like cytopathology due to the challenges of digitizing 3D cell structures.^18^

Whereas commercial platforms exist, the availability of open-source solutions is paramount in this field.^23^ Open source promotes transparency and reproducibility via accessible source code and fosters collaborative development.^24^ It allows researchers and practitioners to build upon existing work rather than starting anew, adapting tools for specific workflows. Furthermore, open-source democratizes access to advanced analytical tools, especially for institutions with limited resources, spurs innovation through global contributions, and facilitates the standardization and interoperability crucial for widespread DP adoption.^23^, ^24^, ^25^

**Fig. 1:**
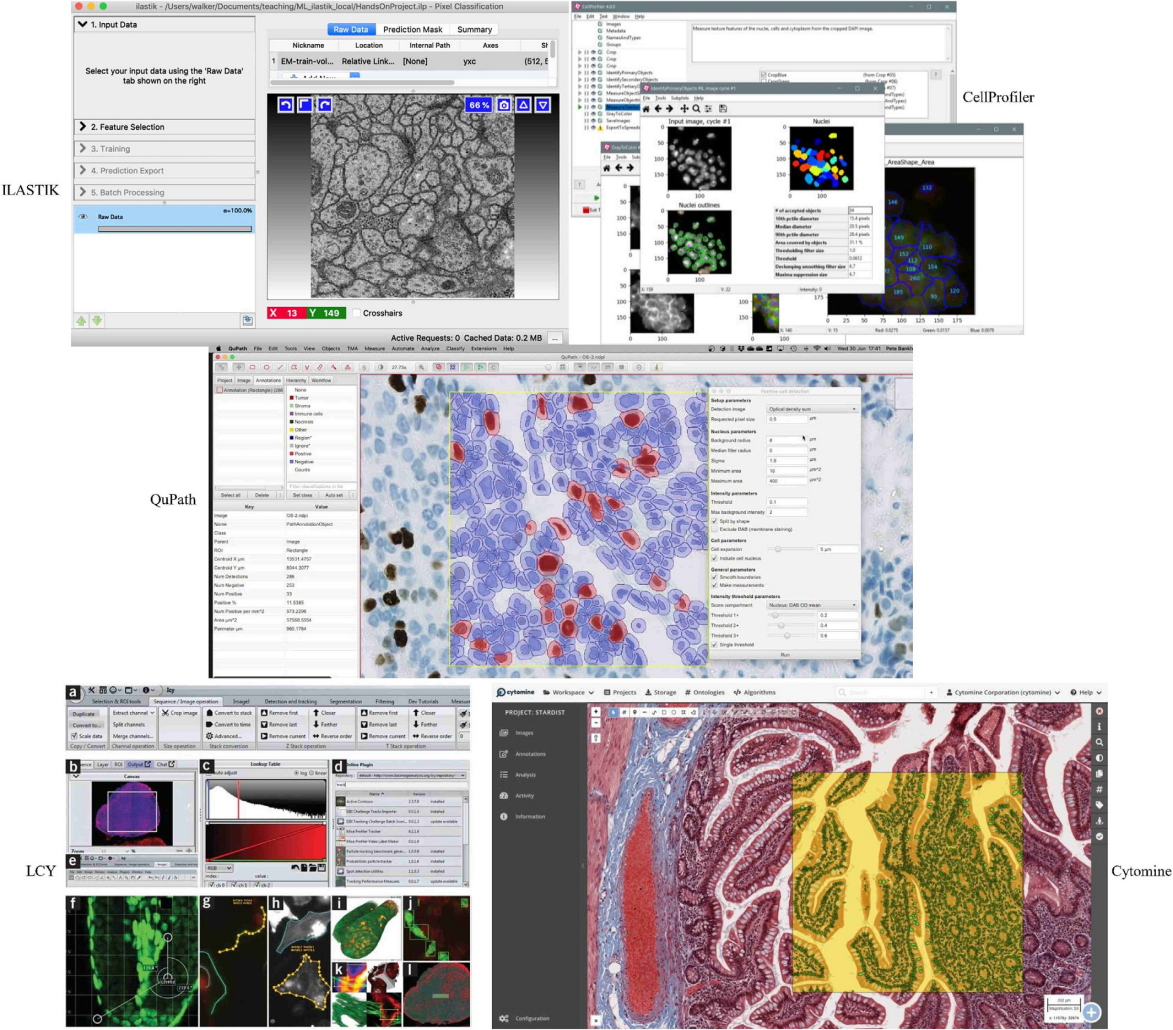
Representative open source software tools for visual analytics in digital pathology. This figure illustrates five prominent tools: QuPath,^26^ Ilastik,^28^ CellProfiler,^27^ ICY,^29^ and Cytomine,^30^ showcasing the diverse capabilities available in open source digital pathology software.

Some key open-source software tools for analysis and visualization of histopathology images are shown in Fig. 1. These range from general-purpose platforms like QuPath^26^ (bioimage analysis) and CellProfiler^27^ (cell image analysis) to more specialized ones like Ilastik^28^ (interactive segmentation), ICY^29^ (cell detection/classification), and Cytomine^30^ (web-based collaborative analysis of multi-gigapixel images). Our interest lies particularly in tools suited for visual analytics tasks beyond basic classification or segmentation. However, the impact of open-source solutions is sometimes limited as many innovative approaches described in the literature remain commercial or unshared. This creates a gap between cutting-edge research and practical tools readily available to the pathology community. Promoting the development and sharing of open-source visual analytics tools is essential to bridge this gap, ensuring wider adoption and refinement of innovations.

Supplementary Table 11, provides a detailed comparison of major open-source bioimage analysis tools currently available for DP. The comparison spans across four key dimensions: core capabilities, performance characteristics, user features, and application focus. QuPath and Cytomine stand out for their robust WSI support and ability to handle large images (>40 GB), whereas Ilastik, CellProfiler, and ICY excel in cell biology applications. Notably, Cytomine is the only platform offering comprehensive web-based collaborative features, making it particularly suitable for multi-user environments. All tools support ML integration and plugin development, though they differ in their specific strengths and primary applications. This comparison highlights how different tools address various needs in the DP workflow, from basic image analysis to sophisticated collaborative research platforms.

To ensure a systematic and objective evaluation of the open-source visual analytics tools, we assessed each based on its developmental stage and a set of predefined criteria designed to compare maturity and practical applicability. We classified tools into three categories:

• **Frameworks:** Foundational pipelines that require additional integration and customization.
• **Tools:** Interactive solutions that perform specific tasks with a focus on usability.
• **Software:** Comprehensive, user-friendly applications with integrated interfaces for multiple tasks. Furthermore, our evaluation considered the following aspects:
• **Core functionalities:** Visualization methods, ML integration, and interactivity.
• **Performance characteristics:** Ability to handle large datasets (e.g., WSIs), processing speed, and scalability.
• **Usability factors:** Ease of integration into clinical workflows, user interface design, and overall user acceptance.

This combined approach allowed for a systematic comparison of each solution's readiness for practical use.

Whereas existing surveys of DP and visual analytics offer valuable insights into computational methods, they often lack comprehensive coverage of available open-source tools and specific visual analytics techniques. This can leave practitioners uncertain about how to effectively implement advanced VA in their workflows. Our systematic review addresses these gaps. As highlighted by the comparison with recent literature (2021–2024) in Table 1, we provide a broader analysis across multiple dimensions, including publication trends, disease spectrum, image modalities, dataset accessibility, visualization methods, user interaction, and clinical implementation readiness. Crucially, our review places a unique emphasis on open-source solutions, specifically evaluating their developmental maturity (categorized as frameworks, tools, or software) to assess their practical availability and applicability for bridging the gap between research and practice.

**Table 1:** Comparison of our review with other histopathology image analysis reviews and surveys from 2021 to 2024.

Comparative dimensions	Our review	^17^	^15^	^14^	^31^	^13^	^12^
Temporal distribution^a^	✓					✓	✓
Journal profile^a^	✓						
Citation metrics^b^	✓						
Disease spectrum^b^	✓	✓	✓	✓	✓	✓	✓
Image modalities^b^	✓	✓	✓	✓	✓	✓	✓
Open-Source Emphasis	✓						
Dataset accessibility^c^	✓	✓	✓		✓	✓	✓
Visualization methodologies^c^	✓	✓		✓			
UI & interaction^c^	✓						✓
Clinical implementation^c^	✓	✓		✓			

a Temporal Distribution: Pattern of publication frequency over time. Journal Profile: Analytical review of journal impact and scope.

b Citation Metrics: Measurement of research impact using Google Scholar citations. Disease Spectrum: Range of histopathological disease classifications. Image Modalities: Various histopathological imaging techniques.

c Dataset Accessibility: Availability and usability of datasets. Visualization Methodologies: Techniques for data representation. UI & Interaction: User interface and engagement methods. Clinical Implementation: Integration into medical workflows.

Despite significant advancements, a systematic evaluation synthesizing the capabilities, limitations, maturity, and practical applications of open-source visual analytics tools for DP has been missing. This review critically analyzes the current landscape of these open-source tools to highlight their utility, assess their developmental stages, and identify key areas for improvement, thereby aiming to facilitate their integration into clinical and research workflows for enhanced diagnostic accuracy and patient care. Specifically, our analysis addresses the following primary research questions:

• **RQ1:** What are the prevalent open-source visual analytics techniques in histopathology imaging, and what stages of development do they encompass (frameworks, tools, and software)?
• **RQ2:** How do visual analytics methods enhance the interpretation of histopathological images, and what is their comparative efficacy?
• **RQ3:** What are the existing challenges in visual analytics for histopathology, and what are the practical considerations for clinical implementation?

The remainder of this article is organized as follows: The second section details the Methods employed in our systematic review, including the Inclusion and exclusion criteria, the process for Study selection and data extraction, and the approach to Data synthesis. The next section presents the Results, starting with Study Selection and Bibliometric Analysis. It then delves into Data Characteristics and Availability, covering Tool Development Trends, Disease Focus, Histopathology Image Modalities, and Dataset Characteristics and Availability. This is followed by an analysis of Technical Approaches and Methodologies, including Data Preprocessing Techniques, Algorithms and Models, and Performance Metrics and Evaluation Methods. Next, we examine Visualization and Interaction Techniques, detailing specific Visualization Techniques, User Interaction Features, a Discussion of Key Open-Source Tools, and their Technical Contributions. The results section concludes with Clinical Applications and Impact, discussing the Strengths and Limitations of Open Source Visual Analytics Solutions, along with their Clinical Applications and Real-World Implementation. The fourth section provides the Discussion, summarizing the Key Findings and outlining Implications and Future Directions. Finally, fifth section offers the Conclusion, summarizing the review's main insights and contributions.

## Methods

This systematic review adhered to the Preferred Reporting Items for Systematic Reviews and Meta-Analyses (PRISMA) guidelines^32^ (details in Supplementary Table 1). We conducted a comprehensive search across Scopus, PubMed, IEEE Xplore, ScienceDirect, ACM, Eurographics DL, and Google Scholar for studies published between January 2000 and July 2024. PubMed includes MEDLINE citations, so a separate MEDLINE search was unnecessary. For Google Scholar, the initial 200 relevant results were reviewed. Reference lists of included studies were also screened.

Search terms identified through prior literature and domain expertise focused on studies applying visual analytics to histopathology images. The core search string was: (“Visual Analytics” OR “Data Visualization” OR “Visual Annotation” OR “Image Visualization” OR “Interactive” OR “Visual Data”) AND (“Histopathology” OR “Digital Pathology” OR “Microscopic Imaging” OR “Slide Image” OR “Tissue Analysis”). This string was adapted for each database; the complete strategy is detailed in Supplementary Table 2.

### Inclusion and exclusion criteria

The authorship team collaboratively defined selection criteria. Included studies must: (1) utilize histopathology modalities; (2) primarily use visual analytics for interacting with and visualizing histopathology images (as per search terms in Supplementary Table 2); (3) focus on developing an interactive tool, framework, or software usable without extensive coding; (4) be open source and publicly available; and (5) be original, peer-reviewed research or conference proceedings in English.

Exclusions comprised studies performing only classical analysis (e.g., regression), simple segmentation/classification/diagnosis tasks, those unrelated to histopathology, non-open source research, non-English publications, and non-original research formats (reviews, abstracts, proposals, editorials, commentaries, and letters).

**Fig. 2:**
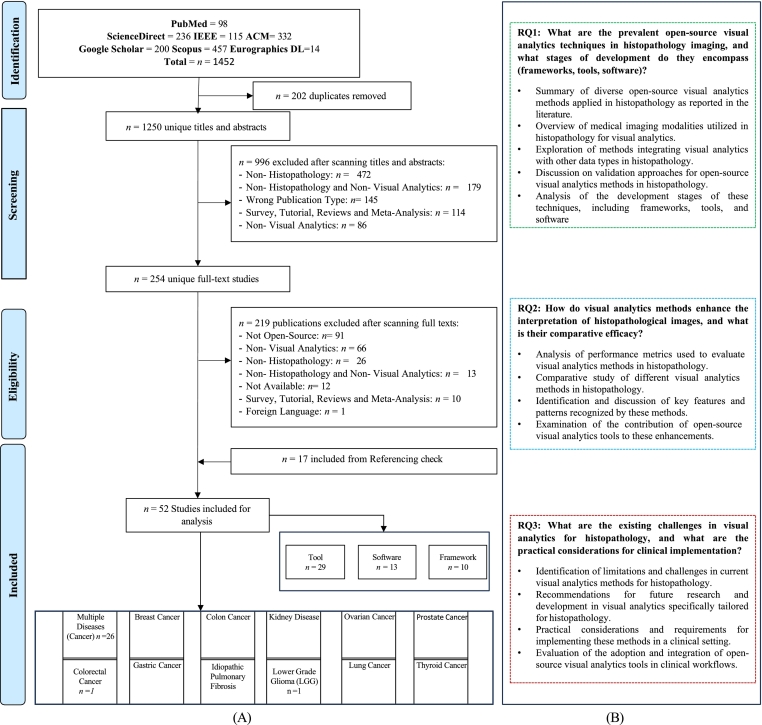
Overview of the study selection process and research questions. (A) PRISMA flow diagram of study selection process for open source visual analytics in histopathology. (B) Research questions posed.

To ensure PRISMA compliance and transparency, a list of all 254 studies screened at the full-text stage, with explicit exclusion reasons, is provided in Supplementary Table 13. This aligns with the process shown in the PRISMA flow diagram (Fig. 2) and supports reproducibility.

### Study selection and data extraction

The Rayyan web tool^33^ facilitated screening and selection. One reviewer (Z.A.) performed the search. After deduplication, titles/abstracts were screened, followed by full-text screening to finalize inclusions. Two reviewers (Z.A., M.AL) independently conducted selection and data extraction, resolving discrepancies through discussion or consultation with a third author (M.A.). A standardized data extraction form, piloted on five studies, captured key information: Paper Title, Authors, Publication Year, Journal Name, Research Objective, Study Design, Methodology, Data Source, Data Preprocessing, Algorithm/Model, Functionality, Performance Metrics, Results, Visualization Techniques, Tool/Application Developed, User Interaction, Histopathology Modalities, Evaluation Methods, Strengths, Limitations, Clinical Application, Open-Source Status, Category, Nickname, Country, Citation, and Histopathology Diseases (details in Supplementary Table 3).

### Data synthesis

We employed a narrative synthesis of the extracted data to identify and analyze open-source visual analytics tools for histopathology images. Given the heterogeneity in modalities, methods, implementations, data, and evaluations, our analysis covered multiple dimensions.

We first examined study characteristics (demographics, aims, and design) for context. Then, we summarized data types and sources used. Technical aspects explored included implementation level, ease of use, citation counts (via Google Scholar) as a proxy for impact, and verification of source code accessibility.

Based on this analysis, studies were categorized as:

• **Frameworks:** Foundational pipelines requiring integration, offering flexibility but potentially needing more technical expertise.
• **Tools:** Interactive interfaces for specific tasks or limited modalities, possibly plugins; offering targeted functionality.
• **Software:** Comprehensive, user-friendly applications with integrated interfaces for multiple tasks and modalities; offering the easiest user experience.

Our synthesis also covered dataset availability, prevalent visualization techniques, reported performance metrics, and underlying algorithms/methods. Finally, we assessed each study's strengths, limitations, and potential clinical applicability to connect research findings with practical healthcare implementation. This comprehensive approach provides a detailed overview of the current state, trends, gaps, and future opportunities in open-source visual analytics for histopathology.

## Results

This section synthesizes key findings from the systematic review, organized thematically. We begin with study selection characteristics and bibliometrics, followed by data analysis (modalities, diseases, and dataset access), a review of methodologies (pre-processing, algorithms, and evaluation), visualization techniques and user interaction, technical contributions, and finally, clinical applications, strengths, and limitations. This structure progresses from a general overview to specific technical and practical aspects of open-source visual analytics in histopathology.

### Study selection and bibliometric analysis

Initial title/abstract screening yielded 254 candidate studies. Following rigorous evaluation against inclusion criteria, 219 articles were excluded. Forward and backward reference screening identified an additional 17 studies. Ultimately, 52 studies were included for comprehensive analysis. The selection process is depicted in the PRISMA diagram (Fig. 2A). Detailed study characteristics are available in Supplementary Tables 4–12.

The corpus comprises 48 journal articles and 4 conference publications, spanning 2006–2024. A notable concentration (*n* = 31) occurred in the last 4 years, indicating accelerating interest. Research contributions are global, led by the USA (*n* = 25), followed by China (*n* = 5), the UK (*n* = 4), and India (*n* = 3), involving 19 countries overall.

**Fig. 3:**
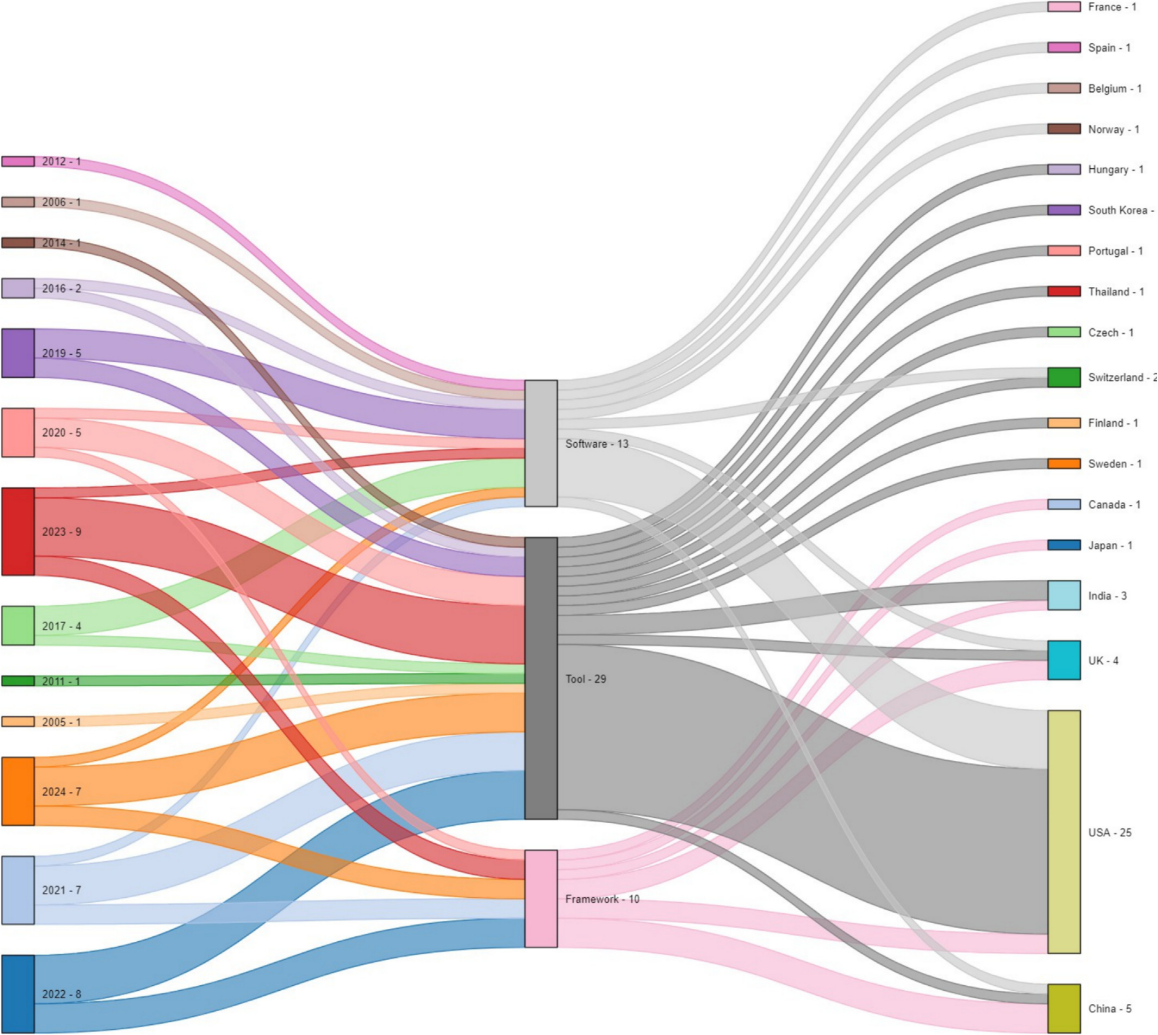
Distribution of open source visual analytics studies in histopathology by type, year (2006–2024), and country, showing trends and geographical research efforts.

The Sankey diagram (Fig. 3) illustrates the distribution of studies by type (tool, software, and framework), publication year, and country. It visually confirms the increase in publications over time, particularly since 2019, with peaks in 2022–2023, contrasting with sparser contributions in earlier years (2005–2014). Tools (*n* = 29) represent the largest category,^34^, ^35^, ^36^, ^37^, ^38^, ^39^, ^40^, ^41^, ^42^, ^43^, ^44^, ^45^, ^46^, ^47^, ^48^, ^49^, ^50^, ^51^, ^52^, ^53^, ^54^, ^55^, ^56^, ^57^, ^58^, ^59^, ^60^, ^61^, ^62^ followed by software (*n* = 13),^26^, ^27^, ^28^, ^29^, ^30^^,^^63^, ^64^, ^65^, ^66^, ^67^, ^68^, ^69^, ^70^ and frameworks (*n* = 10),^71^, ^72^, ^73^, ^74^, ^75^, ^76^, ^77^, ^78^, ^79^, ^80^ suggesting a focus on practical, user-oriented solutions. Supplementary Table 10 provides links and setup guidelines for these open-source resources. The data indicate a trend shifting from foundational framework development towards more applied tools and software, reflecting a maturation of the field and demand for clinically integrable solutions. The prevalence of tools suggests a problem-driven approach, highlighting the need for specialized solutions.

It is important to note that while our review focuses on open-source visual analytics, there is a significant emerging trend in histopathology involving large language models (LLMs) and multimodal AI systems. Recent non-open-source studies, outside our scope, demonstrate advancements using vision-language models for diagnostics and analytics.^81^^,^^82^ For example, PathChat integrated pathology-specific vision encoders with LLMs,^81^ and PathAsst emphasized data-driven training.^82^ Newer systems like GPT-4V^83^ and Claude 3^84^ show promise in histopathology tasks, whereas frameworks like MLLM4PUE^85^ and MMed-RAG^86^ aim to improve multimodal embeddings and factuality. These developments highlight critical future directions, including generalization (e.g., AIDA^87^), ethics, and human–AI collaboration, even though they fall outside our specific inclusion criteria.

**Fig. 4:**
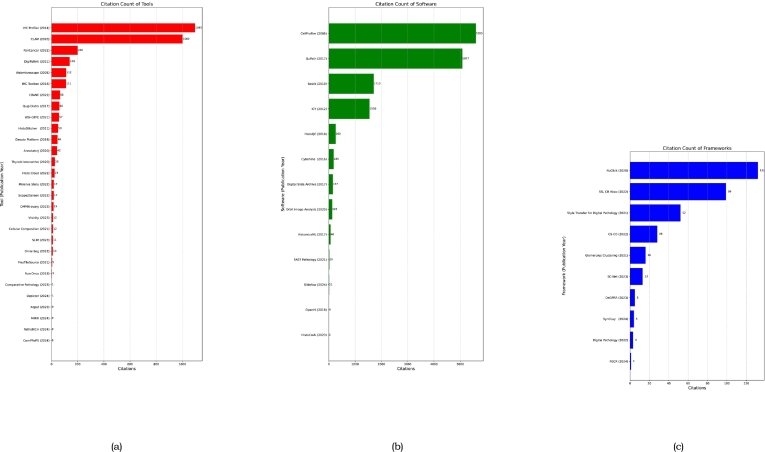
Bibliometric analysis of tools (A), software (B), and framework (C). The y-axis indicates the year and name, whereas the x-axis shows the number of citations.

Our bibliometric analysis (Fig. 4) examines citation trends for frameworks, software, and tools. Fig. 4b (Software) shows CellProfiler^27^ (2006, 5595 citations) maintaining long-term impact, whereas QuPath^26^ (2017, 5077 citations) demonstrates rapid recent adoption. ICY^29^ (2012, 1550 citations) is also notable. Supplementary Table 11 details comparisons of major tools like QuPath, Cytomine, Ilastik, CellProfiler, and ICY across capabilities, performance, user features, and application focus, highlighting their respective strengths (e.g., WSI support in QuPath/Cytomine, collaborative features in Cytomine, cell biology focus in others). Fig. 4c (Frameworks) features NuClick^74^ (2020, 132 citations) and SSL CR Histo^79^ (2022, 99 citations), indicating newer frameworks can gain traction quickly. Fig. 4a (Tools) highlights IHC Profiler^59^ (2014, 1097 citations) for sustained importance and CLAM^61^ (2020, 1002 citations) for rapid recent relevance. Comparing categories, software like CellProfiler^27^ and QuPath^26^ lead in citations, followed by tools like IHC Profiler,^59^ reflecting their foundational or pivotal roles. Overall, the analysis shows both established and innovative solutions contribute significantly, reflecting a dynamic research landscape.

**Fig. 5:**
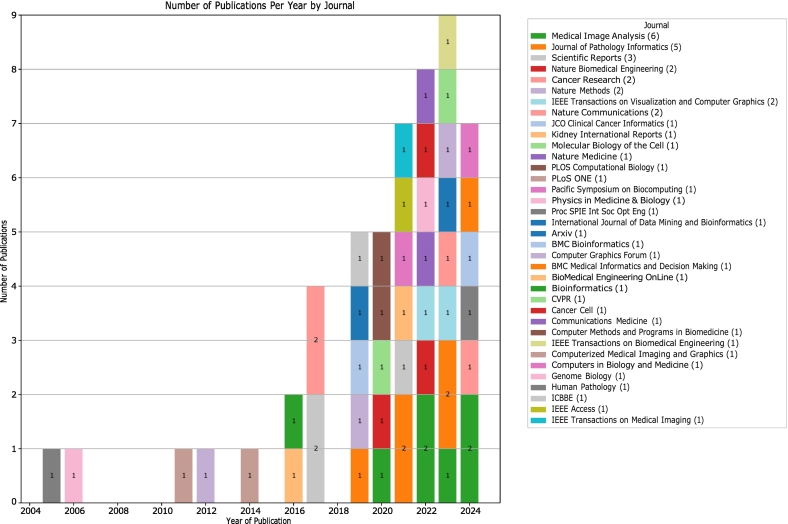
Temporal distribution of publications in open source visual analytics for histopathology by journal and conference (2004–2024).

The distribution of publications by venue (Fig. 5) reveals significant growth and diversification over the past two decades, accelerating notably since 2018. *Medical Image Analysis* (*n* = 6) and *Journal of Pathology Informatics* (*n* = 5) are leading journals, followed by *Scientific Reports* (*n* = 3). Publications in high-impact journals like *Nature Biomedical Engineering*, *Nature Methods*, and *Nature Communications* (*n* = 2 each) underscore the quality of research. The wide range of journals and conferences (including CVPR, ICBBE) reflects the field's interdisciplinary nature, bridging medical imaging, pathology, and computer science. The presence of open-access platforms (*PLoS ONE*, *arXiv*) suggests a trend towards accessible dissemination. This diverse publication landscape, combining specialized and general venues, fosters cross-pollination of ideas. The recent surge and venue diversity indicate a rapidly evolving field gaining momentum and recognition, poised for further integration and potentially transformative advancements.

### Data characteristics and availability

Our systematic review of visual analytics in histopathology imaging revealed significant trends in tool development, disease focus, and data modalities. These findings provide crucial insights into the current landscape of visual analytics and highlight areas for future research and development. We detailed this in Supplementary Tables 5.

#### Tool development trends

The analysis of visual analytics tools, as defined in our methodology, revealed a clear preference for standalone tools (*n* = 29) over independent software (*n* = 13) and frameworks (*n* = 10). This distribution, detailed in Table 2, suggests a trend towards more specialized, task-specific solutions in histopathology image analysis. The prevalence of standalone tools may indicate a need for flexible, modular approaches that can be easily integrated into existing workflows or customized for specific research questions.

**Table 2:** Distribution of visual analytics studies by type, disease focus, and imaging modality.

Category	No. of studies	Study references
Visual analytics type: Tool	29	34, 35, 36, 37, 38, 39, 40, 41, 42, 43, 44, 45, 46, 47, 48, 49, 50, 51, 52, 53, 54, 55, 56, 57, 58, 59, 60, 61, 62
Visual analytics type: Software	13	26, 27, 28, 29, 30^,^^63^, ^64^, ^65^, ^66^, ^67^, ^68^, ^69^, ^70^
Visual analytics type: Framework	10	71, 72, 73, 74, 75, 76, 77, 78, 79, 80
Disease focus: Multiple diseases (Cancer)	26	27, 28, 29, 30^,^^35^^,^^41^^,^^42^^,^^44^^,^^45^^,^^47^^,^^50^^,^^51^^,^^53^, ^54^, ^55^^,^^59^^,^^60^^,^^62^^,^^63^^,^^68^, ^69^, ^70^^,^^72^, ^73^, ^74^^,^^77^
Disease focus: Breast cancer	6	^36^^,^^61^^,^^65^^,^^66^^,^^79^^,^^80^
Disease focus: Colon cancer	5	^26^^,^^34^^,^^43^^,^^52^^,^^75^
Disease focus: Kidney disease	4	^46^^,^^57^^,^^58^^,^^76^
Disease focus: Ovarian cancer	3	^40^^,^^48^^,^^56^
Disease focus: Prostate cancer	2	^37^^,^^38^
Disease focus: Other specific cancers/diseases	6	^39^^,^^49^^,^^64^^,^^67^^,^^71^^,^^78^
Histopathology image modalities: WSI	46	^26^^,^^28^, ^29^, ^30^^,^^34^, ^35^, ^36^, ^37^, ^38^^,^^40^, ^41^, ^42^, ^43^, ^44^, ^45^, ^46^, ^47^, ^48^, ^49^, ^50^, ^51^, ^52^, ^53^^,^^55^, ^56^, ^57^, ^58^^,^^61^, ^62^, ^63^, ^64^, ^65^, ^66^, ^67^, ^68^, ^69^, ^70^, ^71^, ^72^, ^73^^,^^75^, ^76^, ^77^, ^78^, ^79^, ^80^
Histopathology image modalities: Microscopy images	15	26, 27, 28, 29, 30^,^^45^^,^^46^^,^^59^^,^^60^^,^^69^^,^^71^^,^^74^, ^75^, ^76^, ^77^
Histopathology image modalities: Immunohistochemistry (IHC)	9	^26^^,^^28^, ^29^, ^30^^,^^52^^,^^59^^,^^60^^,^^67^^,^^69^
Histopathology image modalities: Multiplexed tissue imaging	6	^26^^,^^29^^,^^35^^,^^39^^,^^54^^,^^55^

#### Disease focus

Our review uncovered a broad spectrum of disease foci, with a notable emphasis on cancer research. As illustrated in Table 2, the majority of studies (*n* = 26) addressed multiple cancer types, demonstrating the versatility and wide applicability of visual analytics in oncology. This trend aligns with the increasing focus on pan-cancer analyses in the field of visual analytics.

Specific cancer types, including breast cancer (*n* = 6), colon cancer (*n* = 5), ovarian cancer (*n* = 3), and prostate cancer (*n* = 2), also received significant attention. The focus on these cancer types likely reflects their high incidence rates and the availability of large-scale datasets. Interestingly, kidney diseases (*n* = 4) emerged as a notable non-cancer focus, indicating the potential of visual analytics in nephropathology.

The diversity of disease focus, including less common cancers and non-neoplastic conditions, underscores the expanding scope of visual analytics in histopathology. This breadth suggests a growing recognition of the technique's potential to address a wide range of pathological conditions.

#### Histopathology image modalities

We investigated the use of different histopathology image data types and categorized them into four main modalities:

• **Whole slide images (WSIs):** High-resolution digital scans representing entire histological sections from glass slides. These are typically gigapixel-sized images that allow for interactive panning and zooming, forming the basis of most modern DP applications.
• **Multiplexed tissue images:** Images generated using techniques that visualize multiple biomarkers simultaneously on the same tissue section (e.g., cyclic immunofluorescence, mass cytometry imaging, and multiplex IHC), often resulting in multi-channel image data.
• **Microscopy images:** This category refers specifically to traditional static images captured directly through a conventional microscope, typically representing a single field-of-view at a specific magnification. Unlike WSIs, these are not scans of the entire slide and lack the inherent navigability of virtual slides. Examples include standard light microscopy snapshots, fluorescence microscopy images of specific regions, or electron micrographs. These represent a fundamentally different data acquisition approach compared to WSI, often resulting in smaller file sizes but lacking the comprehensive context of the entire tissue section.
• **Immunohistochemistry (IHC) images:** Images specifically showing tissue sections stained using antibodies to detect specific antigens. Whereas often captured as WSIs in modern workflows, this category can also include traditional static microscopy images focused on IHC staining results.

Our analysis revealed a clear predominance of WSIs as the primary data modality, used in 46 out of 52 studies. This prevalence reflects the increasing digitization of pathology workflows and the rich, comprehensive information content captured by scanning entire slides. The widespread use of WSIs also indicates their potential as a standardized format for large-scale visual analytics studies.

Other modalities, including traditional microscopy images (*n* = 15), IHC images (*n* = 9), and multiplexed tissue images (*n* = 6), were also represented, although less frequently. The inclusion of these diverse modalities demonstrates the adaptability of visual analytics techniques to various data types and acquisition methods in histopathology.

It is noteworthy that several studies utilized multiple imaging modalities, as detailed in Table 2. This multimodal approach suggests a trend towards more comprehensive and integrative analyses in visual analytics.

The datasets used (presented in Table 3) show several key trends:

• The Cancer Genome Atlas (TCGA) was the most frequent dataset (11 studies), reflecting a preference for large-scale, multi-disease resources suitable for pan-cancer analyses.
• Specialized datasets focusing on specific diseases or tasks (e.g., CAMELYON, DigestPath) were also common.
• Datasets integrating multiple data types (imaging, genomic, proteomic), such as the Human Cell Atlas and HTAN, indicate a move towards multimodal analysis.
• Most datasets are publicly available with accessible links, supporting reproducibility and open science principles.
• Datasets employing advanced imaging (e.g., CyCIF for multiplexed imaging) signal growing interest in high-dimensional tissue analysis.

**Table 3:** Overview of datasets used in histopathology visual analytics research.

Dataset name	Description	Data Link	Study Ref.
TCGA	Comprehensive collection of genomic changes in various cancer types.	https://portal.gdc.cancer.gov/	^41^^,^^45^^,^^56^^,^^61^^,^^63^^,^^66^, ^67^, ^68^, ^69^^,^^71^^,^^80^
CAMELYON	Breast cancer metastasis detection in lymph nodes.	https://camelyon17.grand-challenge.org/	^36^^,^^43^^,^^61^^,^^79^^,^^80^
NCT-CRC-HE-100 K	Large-scale dataset for colorectal cancer classification.	https://zenodo.org/record/1214456	^71^
Human Cell Atlas	Comprehensive reference map of all human cells.	https://www.humancellatlas.org/	^35^
TCGA-HCC	Hepatocellular Carcinoma dataset from TCGA.	https://portal.gdc.cancer.gov/projects/TCGA-HCC	^71^
HTAN	3D atlases of human cancers.	https://www.htanetwork.org/	^35^^,^^45^
DigestPath	Colon cancer diagnosis and gland segmentation.	https://digestpath2019.grand-challenge.org/	^36^^,^^43^
CDSA	Images associated with TCGA specimens.	https://cancer.digitalslidearchive.org/	^63^
CyCIF	Multiplexed tissue imaging data.	https://www.cycif.org/	^39^^,^^54^
BACH	Breast cancer histology image classification.	https://digitalslidearchive.org/	^65^
PanNuke	Pan-cancer histology dataset for nuclei segmentation.	https://pannuke.org/	^65^^,^^72^
CONIC	Nuclei instance segmentation and classification.	https://conic-challenge.grand-challenge.org/	^72^
MoNuSeg	Multi-organ nuclei segmentation dataset.	https://monuseg.grand-challenge.org/	^73^^,^^74^
PAIP	Liver cancer analysis dataset.	https://paip.grand-challenge.org/	^43^
GlaS	Gland segmentation in histological dataset.	https://glas.grand-challenge.org/	^74^
CoNSeP	Cell nuclei segmentation in pathology images.	https://warwick.ac.uk/fac/cross_fac/tia/data/hovernet/	^75^
CPTAC	Comprehensive proteogenomic analysis of cancer types.	https://cptac-data-portal.georgetown.edu/	^45^
IDR	Public repository of image datasets.	https://idr.openmicroscopy.org/	^50^
OMERO	Software platform for biological microscopy data.	https://www.openmicroscopy.org/omero/	^50^
SCEA	Single-cell gene expression database.	https://www.ebi.ac.uk/gxa/sc/experiments	^50^
SEER	Cancer statistics in the United States.	https://seer.cancer.gov/	^51^
KPMP	Dataset focused on kidney diseases.	https://www.kpmp.org/	^57^
HuBMAP	Molecular and cellular atlas of the human body.	https://hubmapconsortium.org/	^57^
BreastPathQ	Tumor cellularity assessment in breast cancer.	https://breastpathq.grand-challenge.org/	^79^
Human Protein Atlas	Protein expression patterns in human tissues.	https://www.proteinatlas.org/	^59^

The diversity and accessibility of these datasets highlight collaborative research efforts and the importance of data sharing.

In summary, this analysis of data characteristics reveals trends towards specialized tools, a strong focus on cancer (particularly pan-cancer studies), the dominance of WSIs alongside growing multimodal approaches, and increasing use of large, publicly available datasets. These insights can guide future research and development in histopathology visual analytics.

Table 3

### Technical approaches and methodologies data preprocessing techniques

Effective data preprocessing is vital for histopathology image analysis. Table 4 details the techniques employed across the reviewed studies (further details in Supplementary Tables 5–7). Image normalization was the most common technique (*n* = 23 studies, e.g.,^40^^,^^41^^,^^65^^,^^71^), highlighting the need for consistent intensity values across diverse datasets. Patch extraction/sampling (*n* = 15, e.g.,^36^^,^^42^^,^^73^^,^^75^) was also frequent, reflecting the standard practice of analyzing large WSIs in manageable sub-regions.

**Table 4:** Overview of data preprocessing techniques used in histopathology visual analytics research.

Technique	No. of studies	Study Ref.
Image normalization	23	^27^^,^^30^^,^^40^^,^^41^^,^^43^^,^^51^^,^^52^^,^^55^^,^^57^^,^^58^^,^^60^, ^61^, ^62^^,^^65^^,^^67^^,^^69^, ^70^, ^71^^,^^75^^,^^76^^,^^79^^,^^80^
Patch extraction/sampling	15	^28^^,^^36^^,^^42^^,^^46^^,^^49^^,^^56^, ^57^, ^58^^,^^61^^,^^62^^,^^70^^,^^73^^,^^75^^,^^76^^,^^78^
Background removal	11	^30^^,^^40^^,^^41^^,^^43^^,^^55^^,^^57^^,^^58^^,^^61^^,^^62^^,^^69^^,^^76^
Segmentation	10	^27^^,^^28^^,^^48^^,^^51^^,^^56^^,^^61^^,^^62^^,^^65^^,^^68^^,^^70^
Stain normalization/separation	6	^36^^,^^67^^,^^69^^,^^71^^,^^76^^,^^79^
Image resizing/downsampling	6	^27^^,^^46^^,^^70^^,^^75^^,^^76^^,^^80^
Data augmentation	5	^43^^,^^73^^,^^74^^,^^79^^,^^80^
Feature extraction	5	^51^^,^^61^^,^^62^^,^^64^^,^^67^^,^^78^
Conversion to pyramidal format	4	^30^^,^^42^^,^^55^^,^^77^
Color space transformation	4	^43^^,^^52^^,^^79^^,^^80^
Image annotation	3	^35^^,^^48^^,^^54^
Otsu's thresholding	3	^43^^,^^69^^,^^70^
Image stitching	2	^37^^,^^54^
Tile-based processing	2	^64^^,^^69^
Watershed algorithm	2	^48^^,^^67^
Morphological operations	2	^43^^,^^67^
Wavelet compression	1	^37^
NPY format conversion	1	^34^
Image sharpening	1	^37^
TMA dearraying	1	^26^
Nuclei object extraction	1	^72^
DICOM format conversion	1	^45^
SLIC superpixel algorithm	1	^68^
Threshold segmentation	1	^78^
Style transfer	1	^80^
Illumination correction	1	^27^

Background removal (*n* = 11, e.g.,^40^^,^^41^^,^^43^) and segmentation (*n* = 10, e.g.,^48^^,^^51^^,^^65^) are crucial for focusing analysis on relevant tissue and separating structures. Techniques addressing staining variability (stain normalization/separation) and computational load (image resizing/downsampling) were each used in (*n* = 6) studies. Data augmentation and feature extraction (*n* = 5 each) were applied to improve model generalization and capture relevant image characteristics. Less common techniques like wavelet compression, SLIC superpixels, and style transfer (*n* = 1 each) represent potential areas for further exploration. The observed diversity reflects the complexity of histopathology images and varied analytical requirements.

#### Algorithms and models

The algorithms and models used in the reviewed studies are diverse, reflecting the complexity of histopathology analysis tasks (overview in Table 5). CNNs were the dominant approach (*n* = 20 studies, e.g.,^34^^,^^36^^,^^65^^,^^71^), underscoring DL's effectiveness in capturing intricate spatial patterns. Traditional image processing techniques remain relevant (*n* = 10, e.g.,^26^^,^^37^^,^^38^^,^^64^).

**Table 5:** Overview of algorithms and models used in histopathology visual analytics research.

Algorithm/Model	No. of studies	Study Ref.
CNNs	20	^34^^,^^36^^,^^40^^,^^41^^,^^43^^,^^49^^,^^56^^,^^58^^,^^61^^,^^62^^,^^65^^,^^69^, ^70^, ^71^, ^72^, ^73^^,^^75^^,^^76^^,^^78^^,^^80^
U-Net and variants	7	^43^^,^^46^^,^^56^^,^^60^^,^^65^^,^^73^^,^^74^
ResNet and variants	9	^34^^,^^36^^,^^43^^,^^49^^,^^56^^,^^71^^,^^73^^,^^75^^,^^79^
Multiple instance learning (MIL)	4	^56^^,^^61^^,^^62^^,^^78^
Attention-based models	5	^41^^,^^61^^,^^62^^,^^70^^,^^78^
Graph neural networks (GNN)	2	^34^^,^^72^
Generative adversarial networks (GANs)	2	^69^^,^^72^
Support vector machine (SVM)	3	^48^^,^^49^^,^^64^
Random forest	4	^28^^,^^30^^,^^49^^,^^67^
Active learning	2	^67^^,^^70^
Self-supervised learning	3	^56^^,^^71^^,^^79^
Semi-supervised learning	3	^46^^,^^70^^,^^79^
Clustering algorithms	5	^53^^,^^61^^,^^62^^,^^73^^,^^76^
Image processing techniques	10	^26^^,^^27^^,^^37^^,^^38^^,^^45^^,^^51^^,^^52^^,^^59^^,^^64^^,^^66^
Segmentation algorithms	8	^43^^,^^48^^,^^51^^,^^57^^,^^65^^,^^68^^,^^72^^,^^74^
Feature extraction methods	5	^27^^,^^49^^,^^64^^,^^76^^,^^78^
Dimension reduction techniques	2	^34^^,^^76^
Visualization techniques	5	^35^^,^^44^^,^^54^^,^^70^^,^^76^
Web-based frameworks	7	^30^^,^^35^^,^^44^^,^^47^^,^^50^^,^^63^^,^^77^
DL frameworks	3	^57^^,^^65^^,^^69^
Statistical models	2	^48^^,^^52^
Natural language processing	1	^78^

Among CNNs, ResNet architectures were popular (*n* = 9, e.g.,^34^^,^^36^^,^^71^^,^^73^), likely due to their ability to train deep networks effectively on complex datasets. Segmentation algorithms (*n* = 8, e.g.,^43^^,^^65^^,^^72^^,^^74^) and U-Net variants (*n* = 7, e.g.,^43^^,^^65^^,^^73^^,^^74^) highlight the importance of precise tissue/cellular delineation. The use of attention-based models (*n* = 5, e.g.,^41^^,^^61^^,^^62^^,^^78^) and multiple instance learning (MIL) (*n* = 4, e.g.,^56^^,^^61^^,^^62^^,^^78^) indicates growing interest in focusing on relevant regions and handling weakly labeled data.

Advanced techniques like graph neural networks (GNNs) and generative adversarial networks (GANs) (*n* = 2 each) are less common, suggesting areas for future exploration. The single use of natural language processing (NLP)^78^ points towards emerging interdisciplinary approaches. This variety, from traditional ML to advanced DL, reflects the field's multi-faceted nature and rapid evolution in addressing histopathology data challenges.

#### Performance metrics and evaluation methods

A diverse range of performance metrics and evaluation methods are used to quantify and validate algorithms in this field (overview in Table 6). Accuracy was the most frequent metric (*n* = 24 studies, e.g.,^64^^,^^65^^,^^71^^,^^73^), reflecting concern for overall correctness. Area under the ROC curve (AUC) was also common (*n* = 17, e.g.,^34^^,^^51^^,^^52^^,^^76^), providing a comprehensive measure of discriminative ability. F1-score (*n* = 14, e.g.,^34^^,^^65^^,^^71^^,^^73^), often reported alongside precision and recall (*n* = 12 each), indicates the importance of balancing the identification of relevant instances while minimizing false positives. Domain-specific metrics like Dice coefficient (*n* = 8, e.g.,^36^^,^^64^^,^^73^) and intersection over union (IoU) (*n* = 6, e.g.,^40^^,^^58^^,^^73^) are frequently used, reflecting the significance of accurate segmentation.

**Table 6:** Overview of performance metrics and evaluation methods in histopathology visual analytics research.

Performance metrics	No. of studies	Study ref.	Evaluation methods	No. of studies	Study ref.
Accuracy	24	^27^^,^^30^^,^^48^^,^^49^^,^^51^^,^^52^^,^^56^, ^57^, ^58^^,^^61^^,^^62^^,^^64^, ^65^, ^66^^,^^69^, ^70^, ^71^^,^^73^^,^^76^^,^^80^	Cross-validation	15	^34^^,^^41^^,^^48^^,^^49^^,^^52^^,^^56^^,^^58^^,^^61^^,^^62^^,^^69^, ^70^, ^71^^,^^76^^,^^79^^,^^80^
AUROC/AUC	17	^27^^,^^34^^,^^41^^,^^51^^,^^52^^,^^56^, ^57^, ^58^^,^^61^^,^^62^^,^^67^^,^^69^^,^^70^^,^^72^^,^^76^^,^^79^^,^^80^	Comparison with baseline/other models	12	^26^^,^^27^^,^^34^^,^^41^^,^^43^^,^^47^^,^^52^^,^^56^^,^^69^^,^^71^^,^^72^^,^^78^
F1-score	14	^30^^,^^34^^,^^46^^,^^49^^,^^51^^,^^56^, ^57^, ^58^^,^^65^^,^^69^, ^70^, ^71^^,^^73^^,^^74^	Visual assessment by experts	13	^26^^,^^30^^,^^41^^,^^48^^,^^51^^,^^55^, ^56^, ^57^^,^^64^^,^^67^^,^^69^^,^^72^^,^^73^
Precision	12	^30^^,^^40^^,^^49^^,^^51^^,^^56^, ^57^, ^58^^,^^65^^,^^70^^,^^73^^,^^75^^,^^80^	Ablation studies	6	^46^^,^^56^^,^^71^^,^^72^^,^^78^^,^^79^
Recall	12	^30^^,^^40^^,^^49^^,^^51^^,^^56^, ^57^, ^58^^,^^65^^,^^70^^,^^73^^,^^75^^,^^80^	Statistical analysis	7	^26^^,^^41^^,^^56^^,^^59^^,^^67^^,^^71^^,^^76^
Dice coefficient/score	8	^36^^,^^43^^,^^46^^,^^48^^,^^56^^,^^64^^,^^73^^,^^74^	External validation	5	^46^^,^^56^^,^^61^^,^^69^^,^^70^
Mean average precision (MAP)	5	^42^^,^^49^^,^^57^^,^^75^^,^^78^	User feedback/testing	6	^30^^,^^35^^,^^39^^,^^44^^,^^53^^,^^68^
Intersection over union (IoU)	6	^40^^,^^57^^,^^58^^,^^60^^,^^73^^,^^80^	Case studies	4	^29^^,^^44^^,^^50^^,^^54^
Cohen's kappa	4	^36^^,^^43^^,^^57^^,^^58^	Kaplan-Meier analysis	4	^26^^,^^41^^,^^56^^,^^69^
Hausdorff distance	4	^46^^,^^55^^,^^57^^,^^74^	Runtime analysis	4	^39^^,^^65^^,^^68^^,^^80^

Regarding evaluation, cross-validation was the most common method (*n* = 15 studies, e.g.,^34^^,^^48^^,^^49^^,^^71^), emphasizing the need to assess model generalizability. Comparison with baselines/other models (*n* = 12, e.g.,^34^^,^^71^^,^^72^) and visual assessment by experts (*n* = 13, e.g.,^26^^,^^41^^,^^64^) highlight the dual importance of quantitative benchmarking and qualitative expert validation. Ablation studies (*n* = 6, e.g.,^46^^,^^71^^,^^72^) and statistical analysis (*n* = 7, e.g.,^26^^,^^41^^,^^71^) provide deeper insights into model behavior and result significance. The use of external validation (*n* = 5, e.g.,^46^^,^^56^^,^^69^) and user feedback/testing (*n* = 6, e.g.,^35^^,^^39^^,^^44^) underscores the focus on real-world applicability and usability.

### Visualization and interaction techniques

This subsection analyzes the visualization and user interaction features crucial for effective visual analytics in histopathology (details in Supplementary Tables 8).

#### Visualization techniques

**Fig. 6:**
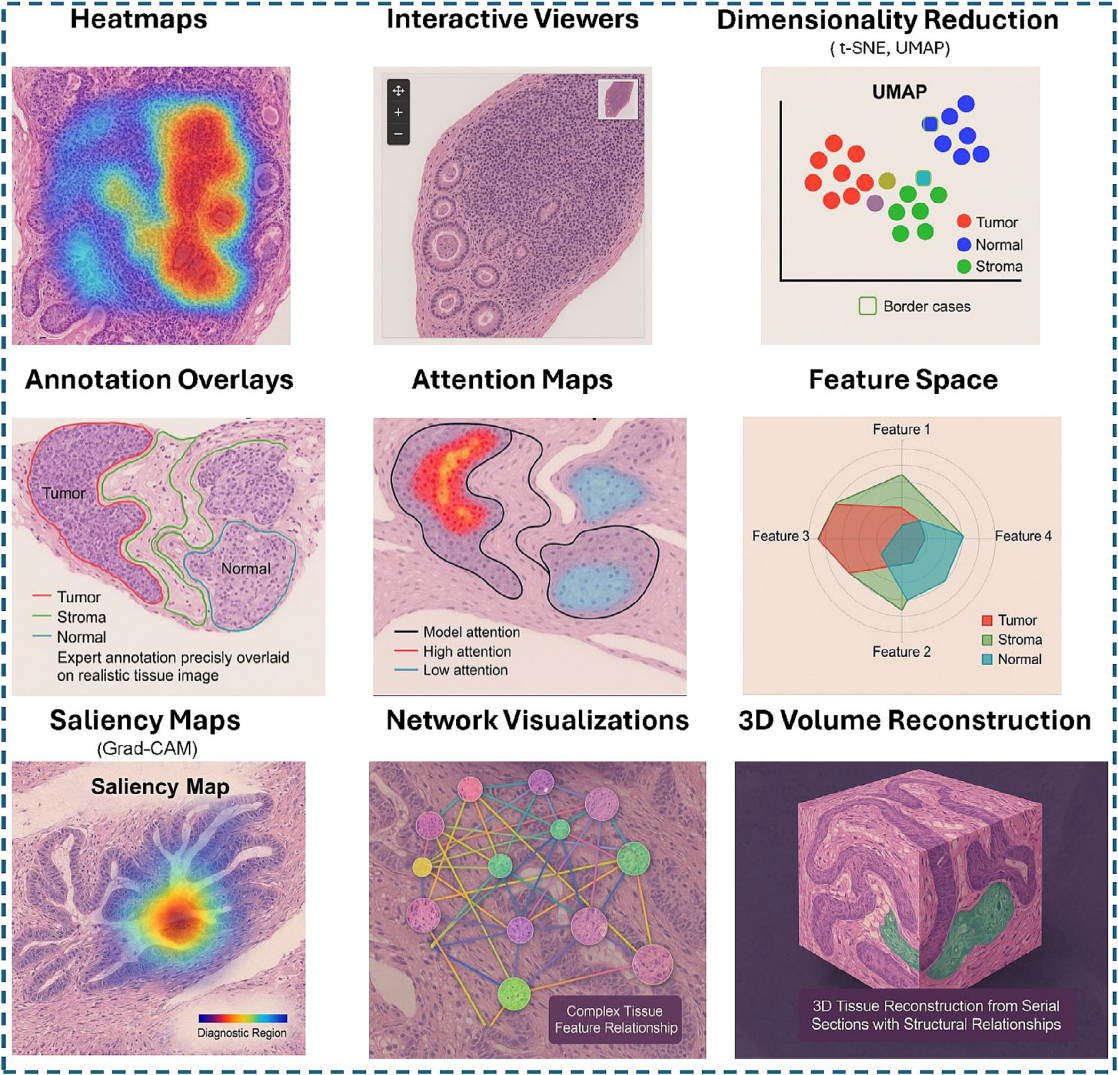
This figure illustrates common approaches reported in Table 7, showcasing nine distinct visualization methods: Heatmaps: displaying spatial distribution of cancer probability across tissue structures. Interactive viewers: enabling multi-resolution WSI exploration with navigation tools. Dimensionality reduction techniques (t-SNE, UMAP): revealing tissue type clustering patterns. Annotation overlays: precisely marking tumor, stroma, and immune components. Attention maps: highlighting cellular structures of model focus. Feature space visualization: comparing multi-dimensional tissue characteristics. Saliency maps (including Grad-CAM): identifying diagnostically relevant regions. Network visualizations: displaying weighted relationships between tissue components. 3D volume reconstruction: showing structural relationships across tissue sections. These complementary visualization approaches support interpretation of complex histopathological data.

A comprehensive overview of visualization techniques employed in the reviewed studies is presented in Table 7, with representative visual examples showcased in Fig. 6. Heatmaps were the most prevalent method (*n* = 17 studies), effective for showing spatial distributions of features or prediction confidences. Interactive viewers (zoomable/tile-based interfaces) were second most common (*n* = 12), essential for navigating large WSIs at multiple resolutions, often incorporating annotation overlays (*n* = 10) to link expert knowledge directly onto images. Dimensionality reduction plots (scatter plots, UMAP, and t-SNE) were used in (*n* = 11) studies, vital for visualizing high-dimensional data comprehensibly.

**Table 7:** Visualization techniques in histopathology visual analytics.

Visualization technique	No of studies	Study references
Heatmaps	17	^26^^,^^40^^,^^41^^,^^43^^,^^44^^,^^51^^,^^53^^,^^56^^,^^58^^,^^65^, ^66^, ^67^^,^^69^, ^70^, ^71^^,^^73^^,^^78^
Interactive viewers (e.g., zoomable, tile-based)	12	^29^^,^^30^^,^^35^^,^^39^^,^^44^^,^^45^^,^^47^^,^^57^^,^^63^, ^64^, ^65^^,^^77^
Scatter plots/UMAP/t-SNE	11	^26^^,^^34^, ^35^, ^36^^,^^39^^,^^49^^,^^51^^,^^56^^,^^58^^,^^61^^,^^69^
Annotation overlays	10	28, 29, 30^,^^50^^,^^57^^,^^58^^,^^64^^,^^65^^,^^68^^,^^70^
Attention maps	8	^40^^,^^41^^,^^56^^,^^61^^,^^69^^,^^76^^,^^78^^,^^80^
Feature space visualization	6	^36^^,^^49^^,^^56^^,^^61^^,^^62^^,^^69^
Thumbnails	5	^39^^,^^50^^,^^54^^,^^66^^,^^67^
Parallel coordinate plots	4	^29^^,^^39^^,^^58^^,^^66^
Network visualizations	4	^29^^,^^53^^,^^56^^,^^72^
Saliency maps	4	^61^^,^^69^^,^^76^^,^^80^
Bar charts/Histograms	4	^39^^,^^48^^,^^54^^,^^59^
Kaplan–Meier curves	2	^40^^,^^41^
3D volume reconstruction	2	^29^^,^^77^
Doughnut charts	1	^72^
Radial charts	1	^54^
Chessboard view	1	^55^
Mosaic maps	1	^69^
ROC curves	1	^52^
Score-weighted class activation mapping (Score-CAM)	1	^76^
Gradient-weighted class activation mapping (Grad-CAM)	1	^76^
Guided backpropagation	1	^76^
Channel-based rendering	1	^54^
Cell-based rendering	1	^54^
Segmentation with color-coding	1	^54^
Sliding-window search algorithm	1	^54^
Virtual magnification indicator	1	^68^
Line matching view	1	^55^
Warp image view	1	^55^
Annotation view	1	^55^
Gaussian mixture models (GMM)	1	^56^
Conditional GANs	1	^69^
Color-coded annotation layers	1	^57^
Interactive UI for toggling visualization layers	1	^57^
Contour assist mode	1	^60^
U-Net pre-segmentation	1	^60^
Integrated gradients	1	^80^
PCA for feature space visualization	1	^61^
Annotation galleries	1	^30^
Textual search engine	1	^30^
Proofreading tools	1	^30^
Interactive segmentation	1	^28^
Probability maps	1	^28^
Boundary maps	1	^28^
Region labeling	1	^70^
Integrated visual programming framework	1	^29^

Techniques like attention maps (*n* = 8) and feature space visualizations (*n* = 6) reflect the adoption of advanced ML, offering insights into model decision-making. Thumbnails (*n* = 5) provide quick overviews, whereas parallel coordinate plots, network visualizations, saliency maps, and bar charts/histograms (*n* = 4 each) offer various ways to represent complex data relationships. A wide array of more specialized techniques were also employed (e.g., Kaplan–Meier curves, 3D reconstruction, various CAM methods, specific rendering types, and interactive UI elements), each used in one or two studies. The frequent use of multiple techniques within single studies indicates a trend towards comprehensive platforms catering to diverse analytical needs. This diversity underscores the complexity of histopathology data and the field's rapid evolution, integrating advanced computational methods with traditional visualization to enhance interpretation, as illustrated in Fig. 6.

#### User interaction features

User interaction features employed are described in Table 8. Interactive navigation (panning/zooming) was most frequent (*n* = 17 studies), aligning with the common use of interactive viewers and highlighting the need for seamless WSI exploration. Annotation tools were second (*n* = 16), indispensable for pathologists to delineate regions, label structures, and provide ground truth. Data filtering/sorting (*n* = 12) and brushing/linking across visualizations (*n* = 9) enable deeper data exploration and pattern discovery. Interactive exploration via heatmaps/scatter plots was also noted (*n* = 9).

**Table 8:** User interaction features in histopathology visual analytics.

User interaction feature	Number of studies	Study references
Interactive navigation (e.g., panning, zooming)	17	^27^^,^^29^^,^^30^^,^^35^^,^^39^^,^^44^^,^^45^^,^^47^^,^^54^^,^^57^^,^^63^^,^^65^^,^^68^^,^^70^^,^^77^
Annotation tools (e.g., drawing, labeling)	16	^26^^,^^27^^,^^30^^,^^36^^,^^48^^,^^49^^,^^54^^,^^57^^,^^60^^,^^64^^,^^65^^,^^67^^,^^70^^,^^74^^,^^77^
Filtering and sorting of data	12	^27^^,^^29^^,^^30^^,^^39^^,^^51^^,^^52^^,^^58^^,^^66^^,^^69^^,^^70^^,^^72^
Brushing and linking across visualizations	9	^27^^,^^29^^,^^30^^,^^39^^,^^56^^,^^58^^,^^61^^,^^69^^,^^70^
Interactive visualization of results (e.g., heatmaps, scatter plots)	9	^27^^,^^30^^,^^40^^,^^41^^,^^51^^,^^61^^,^^62^^,^^69^^,^^70^
Web-based interface for data management and analysis	8	^29^^,^^30^^,^^53^^,^^54^^,^^57^^,^^63^^,^^68^^,^^70^
Manual refinement of annotations	6	^27^^,^^30^^,^^36^^,^^60^^,^^70^^,^^74^
Collaborative features (e.g., session sharing, storytelling)	5	^27^^,^^29^^,^^30^^,^^44^^,^^70^
Point-and-click interface for non-programmers	3	^27^^,^^29^^,^^70^
Visual programming interface	3	^27^^,^^29^^,^^70^
Tangible interactions (e.g., multi-touch tabletop)	2	^56^^,^^72^
Voice narration	1	^35^
Lensing feature for close-up inspections	1	^54^
Virtual magnification indicator	1	^68^
Drag-and-drop functionality	1	^50^
Menu options for editing cells, adding comments	1	^50^
Linking images	1	^50^
Access to external resources (e.g., OMERO image viewer)	1	^50^
Interactive selection of color regions	1	^52^
Model training and detection phases	1	^52^
Labeling objects by clicking on them in the image	1	^67^
Reviewing predictions and adjusting classification rules	1	^67^
Exploring images at multiple resolutions	1	^67^
Interactive project management	1	^55^
Algorithm management	1	^55^
Visualization of registration results	1	^55^
Interactive retrieval using image or text inputs	1	^78^
Real-time predictions and heatmap generation	1	^69^
Interactive zoom and inspection of mosaic maps	1	^69^
Editing FTU mask boundaries	1	^57^
Choice of color deconvolution	1	^59^
Threshold setting	1	^59^
Batch processing	1	^28^
Save and share analysis pipelines	1	^27^
Develop, share, and extend image analysis protocols	1	^29^

Web-based interfaces (*n* = 8) indicate a shift towards cloud-based, collaborative solutions, supported by features like session sharing/storytelling (*n* = 5). Efforts to improve usability include manual annotation refinement (*n* = 6) and graphical interfaces for non-developers or programming (*n* = 3 each). Various specialized features (tangible interactions, voice narration, lensing, drag-and-drop, model training/visualization tools) were also implemented, reflecting the need for multi-faceted interaction approaches. The common integration of multiple features suggests a move towards comprehensive, user-friendly interfaces supporting the full spectrum of histopathology analysis tasks. Supplementary Table 10 provides practical guidelines (links, installation, usage) for relevant tools, aiming to facilitate their adoption.

#### Discussion of key open-source tools

To illustrate the current landscape, we discuss five prominent open-source tools: QuPath,^26^ Ilastik,^28^ CellProfiler,^27^ ICY,^29^ and Cytomine,^30^ representing diverse approaches to scalability and interactivity.

**QuPath**^26^ is highly popular for WSI analysis, offering robust annotation, segmentation, and an extendable plugin architecture for ML integration. Its interactive interface is optimized for gigapixel images but requires significant computational resources for large datasets.

**Ilastik**^28^ excels in user interactivity with an intuitive brush-based interface for rapid classifier training (segmentation/classification), allowing quick refinement. However, scalability can be limited for extremely large WSIs without pre-processing.

**CellProfiler**^27^ is known for robust quantitative analysis and high-throughput cell image processing via a modular design, valuable for reproducible research measurements. It offers less interactive visualization compared to newer tools, potentially making real-time exploration less intuitive.

**ICY**^29^ provides a versatile platform with many plugins for cell detection/classification. While offering strong analytical capabilities, its interface may have a steeper learning curve than newer platforms, but its flexibility is valuable for advanced tasks.

**Cytomine**^30^ features a web-based architecture supporting collaborative annotation and analysis of multi-gigapixel WSIs. Designed for scalability and concurrent use, it addresses remote collaboration and large data volumes, integrating ML tools. Its reliance on web infrastructure necessitates network stability and server support.

In summary, these tools represent a spectrum: QuPath and Cytomine offer scalable, interactive platforms for large datasets and collaboration; Ilastik provides rapid, user-friendly interactivity; CellProfiler and ICY deliver robust quantitative analysis, sometimes with trade-offs in interactive visualization. Tool selection depends on specific study needs regarding data volume, interactivity requirements, and analytical tasks.

### Technical contributions

The open-source visual analytics solutions for histopathology reviewed in this study demonstrate a rich tapestry of mathematical and algorithmic innovations. These contributions span a wide spectrum of computer vision, ML, and image processing techniques, reflecting the multi-disciplinary nature of visual analytics. For a comprehensive overview of these contributions, the reader is directed to Supplement Table 6.

Several solutions introduce novel network architectures and learning paradigms that push the boundaries of DL in histopathology. CS-CO^71^ presents a unified framework that ingeniously combines generative (cross-stain prediction) and discriminative (contrastive learning) methods, leveraging stain separation and vector perturbation to enhance learning. This approach addresses the critical challenge of stain variability in histopathological images while simultaneously improving feature representation. DMMN-ovary^40^ introduces a deep multi-magnification network with a sophisticated multi-encoder, multi-decoder, multi-concatenation architecture for segmentation, effectively capturing multi-scale information crucial for accurate tissue analysis.

Advanced segmentation techniques feature prominently among the contributions. NuClick^74^ incorporates multi-scale convolutional blocks to capture features at various scales, enabling effective segmentation of both small and large objects. It employs a weighted hybrid loss function, combining soft dice loss and weighted cross-entropy, to address class imbalance and penalize false segmentation. This approach is particularly valuable in histopathology, where object sizes can vary significantly. Omni-Seg^46^ introduces a scale-aware controller that extends dynamic neural networks from single-scale to multi-scale segmentation, adapting to the inherent multi-scale nature of histological structures.

Several solutions focus on improving classification and feature extraction. WSI-GTFE^34^ integrates topological data analysis (TDA) techniques, specifically the Mapper algorithm, to summarize GAN embeddings into interpretable topological structures. This novel approach bridges the gap between the high-dimensional feature spaces of DL models and the need for interpretable results in clinical settings. PanCancer^41^ introduces the use of Kronecker Product for pairwise feature interactions, enhancing the interpretability of multimodal data, which is crucial in integrating diverse data types in cancer research.

Innovative approaches to data representation and processing are evident in several solutions. OpenHI^68^ introduces methods for calculating the number of superpixels based on desired sub-region size and total WSI resolution, as well as a method for calculating virtual magnification. These contributions address the computational challenges of working with large-scale WSIs. Slideflow^69^ presents techniques for efficient tile extraction and real-time stain normalization, crucial for processing large datasets efficiently.

Domain adaptation and generalization, critical challenges in deploying AI models in diverse clinical settings, are addressed by solutions like Style Transfer for Digital Pathology,^80^ which introduces STRAP for better domain generalization. HistoColAi^70^ presents a teacher–student model paradigm for pseudo-supervised learning, enhancing tumor region classification and segmentation accuracy with limited labeled data.

Active learning and user interaction are focus areas for solutions like HistomicsML,^67^ which utilizes instance-based and heatmap-based active learning methods to improve classifier training efficiency. It also introduces novel metrics like the Clustering Index (CI) and Hypertrophy Index (HI) for quantifying spatial patterns and morphological changes in nuclei.

Several solutions introduce novel loss functions and optimization techniques. FGCR^78^ defines anchor-based and prompt-based representations for semantic alignment and introduces multivariate cross-modal loss functions. SSL CR Histo^79^ presents a novel self-supervised pretext task (Resolution Sequence Prediction) and a semi-supervised consistency training paradigm, addressing the challenge of limited labeled data in histopathology.

Spatial attention mechanisms are leveraged in solutions like PathoRICH^56^ and Histo Cloud,^58^ which introduce spatial transformer networks to improve classification and segmentation by focusing attention on relevant sub-areas using affine transformation matrices.

Other notable contributions include the use of Gaussian-based kernel density estimation for multi-scale characterization of tumor–stroma interactions,^48^ the development of robust image analysis algorithms for nuclear segmentation and feature extraction,^51^ and the introduction of statistical modeling of color detection and removal of luminance channel.^52^

This diverse contributions show the fast growing innovation of open source visual analytics for histopathology. They stress the developments of the field towards more precise, faster, and explainable analysis of histopathological images. The existence of a wide number of methods demonstrates that histopathological image analysis is a challenging task and current research tries to tackle issues like stain heterogeneity, multi-scale analysis, lack of labeled data, and the requirement of explainable solutions for clinical applications.

To see a list of specific technical contributions made by each solution as well as corresponding references, the readers are referred to Supplement Table 6. As such, this comprehensive overview of visual analytics highlights the need to advance the studies in this area with more work to be done in this field to drive the advancements of visual analytics as well as its use in clinical settings.

### Clinical applications and impact

This subsection discusses the strengths, limitations, and potential clinical applications of the reviewed open-source visual analytics solutions for histopathology, drawing upon details in Supplementary Tables 8 and 10.

#### Strengths of open source visual analytics solutions

The reviewed solutions exhibit several key strengths (summarized in Table 9). User-friendly interfaces were frequently highlighted (*n* = 22 studies), facilitating adoption. High classification accuracy (*n* = 11) and integration with AI/ML techniques (*n* = 11) demonstrate advanced analytical capabilities. Scalability for large datasets and efficient processing (*n* = 9 each) are crucial for handling typical histopathology data volumes. Interpretable results (*n* = 8) and support for collaborative analysis (*n* = 8) enhance clinical trust and teamwork. Reduced need for manual annotation (*n* = 7), interoperability (*n* = 7), real-time visualization (*n* = 6), and potential for enhancing learning/standardization (*n* = 5) were also noted strengths, collectively indicating potential to improve efficiency, accuracy, and collaboration in histopathology.

**Table 9:** Summary of strengths of open-source visual analytics solutions for histopathology.

Strengths	Study references
High classification accuracy	^26^^,^^28^^,^^34^^,^^41^^,^^48^^,^^51^^,^^52^^,^^56^, ^57^, ^58^^,^^71^
User-friendly interface	26, 27, 28, 29^,^^35^^,^^36^^,^^39^, ^40^, ^41^^,^^50^, ^51^, ^52^^,^^54^^,^^55^^,^^57^^,^^58^^,^^63^, ^64^, ^65^, ^66^^,^^68^^,^^70^
Scalability for large datasets	^26^^,^^27^^,^^39^^,^^44^^,^^51^^,^^57^^,^^63^, ^64^, ^65^
Interpretable results	^26^^,^^28^^,^^34^^,^^41^^,^^48^^,^^61^^,^^62^^,^^76^
Efficient processing and performance	26, 27, 28^,^^38^^,^^43^^,^^44^^,^^47^^,^^65^^,^^69^
Integration with AI/ML techniques	26, 27, 28^,^^42^^,^^46^^,^^49^^,^^60^^,^^64^^,^^65^^,^^70^^,^^75^
Supports collaborative analysis	^26^^,^^30^^,^^44^^,^^50^^,^^63^^,^^68^^,^^70^^,^^72^
Minimal manual annotation required	^28^^,^^40^^,^^67^^,^^73^^,^^74^^,^^78^^,^^79^
Interoperability with various systems/datasets	^26^^,^^27^^,^^35^^,^^45^^,^^53^^,^^55^^,^^63^
Real-time visualization capabilities	^26^^,^^44^^,^^54^^,^^63^^,^^65^^,^^69^
Enhances learning and standardization	^26^^,^^35^^,^^37^^,^^49^^,^^63^
Robust across different datasets/inputs	^26^^,^^28^^,^^52^^,^^71^^,^^74^^,^^79^^,^^80^
Supports 3D reconstruction	^26^^,^^27^^,^^77^
Data-efficient	^26^^,^^28^^,^^61^^,^^62^
Facilitates reproducible research	^26^^,^^27^^,^^29^
High annotation precision	^26^^,^^28^^,^^68^
Handles large-scale WSIs	^26^^,^^63^^,^^65^^,^^78^
High predictive performance	^26^^,^^28^^,^^56^
Comprehensive and flexible	26, 27, 28^,^^69^
Adaptable for different tissue types and stains	^26^^,^^27^^,^^58^
Reduces observer bias	^26^^,^^28^^,^^59^
Accelerates annotation tasks	^26^^,^^28^^,^^60^
Improves generalizability	^26^^,^^28^^,^^80^

#### Limitations of open source visual analytics solutions

Despite strengths, these solutions face limitations (outlined in Table 10). High computational resource requirements were the most common issue (*n* = 19 studies), potentially hindering adoption in resource-limited settings. Many solutions are limited to specific histopathology types or stains (*n* = 14), restricting generalizability. Limited user studies or usability evaluations (*n* = 12) suggest a need for more real-world clinical assessment.

**Table 10:** Summary of limitations of open source visual analytics solutions for histopathology.

Limitations	Study references
High computational resource requirements	^26^^,^^27^^,^^36^^,^^38^^,^^41^^,^^42^^,^^51^^,^^57^^,^^58^^,^^61^^,^^62^^,^^64^, ^65^, ^66^^,^^70^^,^^71^^,^^73^^,^^75^^,^^80^
Limited to specific histopathology types or stains	^26^^,^^41^^,^^42^^,^^46^^,^^48^^,^^49^^,^^51^^,^^52^^,^^57^^,^^58^^,^^66^^,^^70^^,^^71^^,^^78^
Limited user studies or usability evaluation	^26^^,^^39^^,^^49^^,^^57^^,^^58^^,^^65^^,^^66^^,^^68^^,^^71^^,^^73^, ^74^, ^75^
Requires manual annotation or expert input	^34^^,^^35^^,^^42^^,^^48^^,^^59^^,^^60^
Limited validation or need for further testing	^26^^,^^40^^,^^41^^,^^51^^,^^56^^,^^71^
Dependence on data quality or completeness	^30^^,^^35^^,^^64^
Scalability issues with large datasets	^53^^,^^63^^,^^67^^,^^68^
Potential for bias or inter-observer variations	^37^^,^^43^^,^^52^^,^^68^^,^^76^
Limited support for 3D or time-lapse analysis	^27^^,^^39^
Complexity in handling diverse image types	^27^^,^^55^
Interpretability challenges	^41^^,^^75^
Dependence on specific software or hardware	^65^^,^^69^^,^^77^
Limited annotation features	^54^^,^^65^
Requires internet connectivity	^50^
Limited to predefined workflows	^28^
Initial setup complexity	^27^^,^^29^^,^^70^
Dependence on community contributions	^29^
Performance variability with different inputs	^36^^,^^47^
Limited to specific tasks or analyses	^79^
Requires large datasets for training	^61^^,^^62^^,^^80^

Regulatory approval significantly influences clinical adoption. Whereas the FDA has cleared some commercial DP platforms,^88^ open-source tools require rigorous validation to meet regulatory standards for diagnostic use, as demonstrated by QuPath's accreditation in a UK lab.^89^ Most current open-source tools (e.g., CellProfiler, Fiji, and Icy) are primarily validated for research.^88^ Transitioning to clinical diagnostics necessitates extensive validation following frameworks like FDA guidelines for software as a medical device, including documented analytical validation, standard operating procedures, quality assessment participation, and version control.^90^^,^^91^ Our analysis focuses on research applications, acknowledging that clinical implementation requires additional regulatory validation specific to jurisdictional requirements, especially if the software is classified as a medical device based on intended use.^88^

Other limitations include scalability issues (*n* = 4), potential for bias/interobserver variations (*n* = 5), limited support for 3D/time-lapse analysis (*n* = 2), complexity with diverse image types (*n* = 2), interpretability challenges (*n* = 2), and software/hardware dependencies (*n* = 3). These highlight areas for future development.

#### Clinical applications and real-world implementation

Potential clinical applications of open-source visual analytics solutions are diverse and impactful (summarized in Table 11). Diagnostic support and decision-making were most frequently cited (*n* = 25 studies), highlighting a key role in assisting pathologists. Cancer research and biomarker discovery (*n* = 18), along with general image analysis/visual analytics (*n* = 16), are significant applications. Prognostic prediction/risk stratification (*n* = 13), education/training (*n* = 11), and treatment planning/personalized medicine (*n* = 11) are also prominent. Other applications include automated tissue segmentation/quantification (*n* = 10), collaborative research/telepathology (*n* = 9), quality control/standardization (*n* = 8), clinical trial support (*n* = 6), tumor subtyping (*n* = 5), and various other specialized uses (*n* = 1 to *n* = 5 each).

**Table 11:** Summary of clinical applications of open source visual analytics solutions for histopathology.

Clinical application	Number of studies	Studies references
Diagnostic support and decision-making	25	^26^^,^^35^, ^36^, ^37^^,^^39^, ^40^, ^41^, ^42^, ^43^, ^44^^,^^49^^,^^51^^,^^56^^,^^57^^,^^61^^,^^62^^,^^64^^,^^65^^,^^67^^,^^69^^,^^70^^,^^72^^,^^73^^,^^78^^,^^80^
Cancer research and biomarker discovery	18	^26^^,^^39^, ^40^, ^41^, ^42^^,^^44^^,^^51^^,^^53^, ^54^, ^55^^,^^59^^,^^63^^,^^65^^,^^68^^,^^70^, ^71^, ^72^^,^^79^
Image analysis and visual analytics	16	^27^^,^^28^^,^^30^^,^^38^^,^^45^^,^^46^^,^^58^^,^^63^, ^64^, ^65^, ^66^^,^^68^^,^^71^^,^^73^^,^^74^^,^^79^
Prognostic prediction and risk stratification	13	^26^^,^^34^^,^^40^^,^^41^^,^^48^^,^^56^^,^^59^^,^^60^^,^^65^^,^^67^^,^^69^^,^^76^^,^^77^
Education and training	11	^29^^,^^35^, ^36^, ^37^^,^^42^^,^^44^^,^^49^^,^^50^^,^^63^^,^^64^^,^^77^
Treatment planning and personalized medicine	11	^26^^,^^35^^,^^40^^,^^41^^,^^43^^,^^53^^,^^56^, ^57^, ^58^, ^59^^,^^67^
Automated tissue segmentation and quantification	10	^28^^,^^30^^,^^45^^,^^46^^,^^48^^,^^52^^,^^55^^,^^64^^,^^66^^,^^74^
Collaborative research and telepathology	9	^29^^,^^35^^,^^42^^,^^44^^,^^50^^,^^53^^,^^61^, ^62^, ^63^
Quality control and standardization	8	^37^^,^^48^^,^^63^^,^^66^^,^^68^^,^^76^^,^^77^^,^^79^
Clinical trials support	6	^26^^,^^40^^,^^51^^,^^53^^,^^56^^,^^57^
Tumor subtyping and classification	5	^40^^,^^41^^,^^70^, ^71^, ^72^
Patient stratification and risk assessment	5	^34^^,^^40^^,^^41^^,^^56^^,^^65^
Prescreening and diagnosis	4	^42^^,^^48^^,^^65^^,^^66^
Epidemiological studies	3	^51^^,^^53^^,^^59^
Precision medicine and personalized diagnosis	3	^53^^,^^56^^,^^59^
Multimodal image analysis	2	^38^^,^^55^
Computational model training	2	^68^^,^^74^
Tissue-based investigations	1	^77^
Histological biomarker quantification	1	^52^
Renal disease pathology research	1	^58^

Case studies exemplify the potential real-world impact. QuPath^26^ has been used in clinical workflows for breast cancer analysis, improving diagnostic accuracy and supporting treatment decisions through biomarker quantification.^89^ Cytomine^30^ facilitates multi-center telepathology, enabling remote consultations and standardized analysis. Ilastik^28^ and CellProfiler^27^ support clinical research through automated segmentation and quantitative analysis for biomarker discovery and risk stratification in trials. These examples underscore the potential for open-source tools to enhance precision, reproducibility, and accessibility in histopathology.

Successful implementation in clinical environments validates practical utility. Specific examples of real-world clinical deployments include:

• **QuPath implementation:** Deployed in hospitals like Belfast Health and Social Care Trust (meeting UK accreditation standards^26^) and Leeds Teaching Hospitals NHS Trust.^92^ Used for routine breast cancer biomarker quantification, showing high concordance with manual assessment and reduced inter-observer variability in Belfast.^93^ Integrated into Leeds' DP initiative, improving diagnostic consistency and reducing quantitative assessment time by 30%.^94^ Leeds has published validation guidelines.^92^
• **Cytomine in collaborative diagnostics:** Deployed in European hospital networks for remote consultations and standardized analysis. At University Hospital of Liège, it facilitated telepathology for regional hospitals, achieving 98.2% diagnostic concordance between digital and conventional microscopy for primary diagnosis.^30^
• **CellProfiler/Ilastik in clinical research:** Whereas primarily research tools, CellProfiler was validated at Massachusetts General Hospital for quantifying immune cell infiltration in colorectal cancer specimens for clinical trials, showing 92% concordance with manual assessment and reducing analysis time >75%.^95^

Common barriers to clinical implementation include integration with LIS, computational resource requirements, and the need for comprehensive validation protocols.^96^ Institutions like Leeds address these challenges with phased implementation strategies and thorough training/competency assessments, proving effective in overcoming adoption resistance.^97^ Regulatory frameworks (e.g., FDA) guide the validation process; whereas open-source tools themselves may not be classified as medical devices, their use in clinical diagnosis requires adherence to these standards.^98^

These examples demonstrate that successful integration is achievable, offering tangible benefits in accuracy, efficiency, and collaboration, and providing valuable roadmaps for wider adoption in hospital settings.

## Discussion

Our comprehensive systematic review illuminates the rapidly evolving landscape of open-source visual analytics in histopathology, situated at the dynamic intersection of AI, computer vision, and the ongoing digital transformation of pathology. The findings underscore the substantial potential of these tools to revolutionize histopathological analysis while simultaneously highlighting critical challenges and areas demanding future research and development. The field has witnessed significant growth, evidenced by the surge in publications over the past 4 years (2020–2024). This momentum reflects the increasing recognition of open-source solutions' value in advancing visual analytics, aligning with the broader movement towards open science, reproducibility, and collaborative innovation in biomedicine. Whereas contributions are globally distributed, indicating diverse perspectives, there is a noticeable concentration in the United States. Concurrently, our analysis reveals a maturation trend, marked by a clear preference for practical, user-oriented standalone tools (*n* = 29) over more foundational software (*n* = 13) or frameworks (*n* = 10). This suggests a shift from exploratory development towards creating applications readily integrable into specific pathology workflows, particularly focused on high-impact areas like cancer research (especially breast, colon, and ovarian cancers). Technologically, the field leverages a rich tapestry of innovations. The dominance of WSIs as the primary data modality (46/52 studies) mirrors pathology's digital shift, enabling advanced AI applications and facilitating remote collaboration. Algorithmically, CNNs, particularly ResNet variants, are prevalent, consistent with broader AI trends in medical imaging. However, the emergence of more sophisticated techniques like attention mechanisms, GANs, and self-supervised learning signals a push towards more nuanced and efficient data analysis. Notably, novel approaches are tackling key issues, such as CS-CO's^71^ framework for stain variability and WSI-GTFE's^34^ use of TDA for enhancing model interpretability—a critical need in clinical settings.

A growing emphasis on user-centric design is evident in the visualization and interaction techniques employed. The common use of heatmaps for spatial insights, interactive viewers for navigating large WSIs, dimensionality reduction plots for high-dimensional data, and annotation overlays reflects efforts to present complex results intuitively and integrate pathologist expertise. Advanced features like attention maps and real-time visualization further suggest a move towards more dynamic and collaborative DP workflows.

Significant challenges to clinical translation:

• **Computational demands**: High-computational resource requirements (reported in 19/52 studies) are a major barrier, particularly in resource-constrained settings. The gigapixel nature of WSIs demands substantial processing power, high-end GPUs, and large memory capacities for storage, real-time visualization, and analysis, often leading to processing times unsuitable for immediate diagnostic needs.
• **Generalizability and robustness**: Many tools exhibit limitations, often being specific to certain histopathology types or stains (14 studies). Achieving robust performance across diverse tissue types, staining protocols, and scanning variations remains a challenge, necessitating the development of more generalizable solutions.
• **Clinical integration and validation**: Integrating these tools into established clinical workflows presents considerable difficulties. Seamless interoperability with LIS, adherence to stringent regulatory standards (e.g., FDA, College of American Pathologists (CAP)), and robust data security and privacy protocols are essential but often lacking in open-source solutions. Furthermore, a critical gap exists in rigorous clinical validation; the scarcity of external validation studies (only a few identified) and comprehensive user/usability evaluations (reported in only 12 studies) hinders assessment of real-world performance and pathologist acceptance. Addressing these integration and validation challenges is paramount for translating potential benefits into routine practice.

Nevertheless, the potential clinical applications are vast. Diagnostic support and decision-making were the most cited applications (22 studies), highlighting the potential to augment pathologist accuracy and efficiency. Applications in cancer research, biomarker discovery, and prognostic prediction further underscore the role these tools can play in advancing personalized medicine.

### Key findings

Our review uncovered several important findings that illuminate the current state and future directions of open-source visual analytics in histopathology:

• **Growth and tool preference:** Substantial growth (2020–2024) with a clear preference for standalone tools (*n* = 29), suggesting demand for specialized, integrable solutions.
• **Data modality and algorithms:** WSIs dominate (46/52 studies); CNNs are the most prevalent algorithm (20 studies).
• **Visualization and interaction:** Emphasis on user-friendly interfaces (heatmaps, interactive viewers, annotation tools).
• **Clinical applications:** Wide potential, led by diagnostic support (22 studies).
• **Performance reporting gaps:** Variable reporting quality, limited calibration/multi-metric reporting hinders generalizability assessment.
• **Limited external validation:** Scarcity raises concerns about model generalizability and practical implementation.
• **Interpretability needs:** Lack of focus on model interpretability/explainability is a critical gap for clinical trust.

### Implications and future directions

Based on our findings, we propose several implications and directions for future research:

• **Flexible, interoperable solutions:** Focus on modular, easily integrated tools compatible with LIS/DP platforms.
• **Multimodal integration:** Explore models integrating WSIs with other data (genomics, clinical) for holistic patient views.
• **Explainable AI (XAI):** Prioritize interpretable models and clear explanations using histopathology-adapted XAI methods.
• **User-centric design and validation:** Conduct extensive user studies and clinical validations to bridge the technology adoption gap.
• **Rigorous external validation:** Prioritize external validation in diverse settings and populations for clinical readiness.
• **Standardized reporting:** Adopt standardized evaluation metrics (accuracy, precision, recall, calibration) for consistent comparison.
• **Collaboration:** Foster collaboration between developers, pathologists, and IT specialists.
• **Education and training:** Invest in programs to equip pathologists with skills for utilizing these tools.

Looking ahead, the evolution of open-source visual analytics must embrace emerging technologies. Integrating multimodal data (genomics, proteomics, and clinical) with imaging can enable more comprehensive disease models, improved biomarker discovery, and precise patient stratification. Concurrently, advancements in AI, particularly LLMs, offer potential for bridging image analysis and NLP—enabling automated report generation, integration of unstructured notes, and context-aware insights. Future research should focus on robust multimodal fusion pipelines and exploring LLM utility.

Furthermore, establishing rigorous standardization and validation frameworks is crucial for clinical adoption. Adherence to standards like DICOM for DP and OME-TIFF^99^ for bioimaging data ensures interoperability. Aligning with regulatory guidelines (e.g., U.S. FDA,^100^ CAP) and implementing comprehensive clinical validation (external validation, multi-center trials, and SOPs) are essential for building trust and meeting benchmarks.

Finally, careful consideration of ethical and regulatory challenges is paramount. Ensuring data privacy, security, algorithmic transparency, accountability, and governance, as highlighted by recent studies,^101^, ^102^, ^103^, ^104^ is fundamental for the safe, effective, and trustworthy deployment of AI tools in clinical workflows. Addressing these multifaceted challenges is key to realizing the full potential of open-source visual analytics in transforming pathology practice.

## Conclusion

This systematic review highlights the transformative potential of open-source visual analytics in histopathology, enhancing diagnostic accuracy, efficiency, and research capabilities. The recent publication surge underscores their growing importance. Key findings show a preference for flexible standalone tools, the dominance of WSIs reflecting digital transformation, and the effectiveness of CNNs. User-centric design focusing on advanced visualization and interaction is critical. Significant challenges persist, including high-computational demands, limited generalizability, insufficient external validation, and the need for comprehensive user evaluations. Future research must prioritize developing robust, generalizable, and interpretable solutions, alongside rigorous clinical validation and standardized evaluation. Fostering collaboration and investing in education are vital. Emerging trends like integrating multimodal data and leveraging LLMs/LMMs offer promising avenues for creating more powerful and intuitive diagnostic tools, representing important future directions. Realizing the full potential requires balancing technological innovation with practical clinical applicability, ensuring these tools genuinely enhance pathology practice and improve patient care.

## CRediT authorship contribution statement

Z.A. and M.AL. contributed to conceptualization. M.A. searched the electronic databases and conducted backward and forward reference list checking. Z.A. and M.AL. performed screening, study selection, and data extraction. K.A. performed data synthesis and contributed to writing—the original draft. Z.A., M.AL., M.A. and C.C. performed writing—review, and editing. M.A., J.S and C.C. supervised the study. All authors approved the manuscript for publication and agreed to be accountable for all aspects of the work.

## Code availability

This study did not involve the utilization of any custom code or mathematical algorithm.

## Declaration of generative AI and AI-assisted technologies in the writing process

The manuscript was edited for English language and consistency using generative AI tools, specifically ChatGPT-4o and Claude Sonnet 3.5. After using this tool/service, the authors reviewed and edited the content as needed and takes full responsibility for the content of the published article.

## Declaration of competing interest

The authors declare that they have no known competing financial interests or personal relationships that could have appeared to influence the work reported in this article.

## Data Availability

All data generated during this study is provided as Supplementary materials.
