# Supplementary material for: Advancing open-source visual analytics in digital pathology: A systematic review of tools, trends, and clinical applications
Source: J Pathol Inform. 2025 May 23;18:100454. doi: 10.1016/j.jpi.2025.100454 (PMC12210317; doi:10.1016/j.jpi.2025.100454)
Supplement: Supplementary material 1 [file mmc1.pdf]

Supplementary Table 1: PRISMA checklist

Section and topic	Item #	Checklist item	Location where item is reported
**Title**			
Title	1	Identify the report as a systematic review.	Title page
**Abstract**			
Abstract	2	See the PRISMA 2020 for Abstracts checklist (Table 2).	Abstract
**Introduction**			
Rationale	3	Describe the rationale for the review in the context of existing knowledge.	Introduction
Objectives	4	Provide an explicit statement of the objective(s) or question(s) the review addresses.	Introduction
**Methods**			
Eligibility criteria	5	Specify the inclusion and exclusion criteria for the review and how studies were grouped for the syntheses.	Methods
Information sources	6	Specify all databases, registers, websites, organizations, reference lists and other sources searched or consulted to identify studies. Specify the date when each source was last searched or consulted.	Methods
Search strategy	7	Present the full search strategies for all databases, registers and websites, including any filters and limits used.	Supplementary Table 2 (referenced in Methods)
Selection process	8	Specify the methods used to decide whether a study met the inclusion criteria of the review, including how many reviewers screened each record and each report retrieved, whether they worked independently, and if applicable, details of automation tools used in the process.	Methods
Data collection process	9	Specify the methods used to collect data from reports, including how many reviewers collected data from each report, whether they worked independently, any processes for obtaining or confirming data from study investigators, and if applicable, details of automation tools used in the process.	Methods and Supplementary Table 3
Data items	10a	List and define all outcomes for which data were sought. Specify whether all results that were compatible with each outcome domain in each study were sought (e.g., for all measures, time points, analyses), and if not, the methods used to decide which results to collect.	Methods and Supplementary Table 3
Study risk of bias assessment	11	Specify the methods used to assess risk of bias in the included studies, including details of the tool(s) used, how many reviewers assessed each study and whether they worked independently, and if applicable, details of automation tools used in the process.	Methods
Effect measures	12	Specify for each outcome the effect measure(s) (e.g., risk ratio, mean difference) used in the synthesis or presentation of results.	N/A
Synthesis methods	13	Describe the processes used to decide which studies were eligible for each synthesis (e.g., tabulating the study intervention characteristics and comparing against the planned groups for each synthesis (item #5)).	Methods
**Results**			
Study selection	14a	Describe the results of the search and selection process, from the number of records identified in the search to the number of studies included in the review, ideally using a flow diagram (see Fig 1).	Results, Fig. 2A
	14b	Cite studies that might appear to meet the inclusion criteria, but which were excluded, and explain why they were excluded.	Results
Study characteristics	15	Cite each included study and present its characteristics.	Results and Supplementary Table 4 -10.
Risk of bias in studies	16	Present assessments of risk of bias for each included study.	N/A

Results of individual studies	17	For all outcomes, present, for each study: (a) summary statistics for each group (where appropriate) and (b) an effect estimate and its precision (e.g., confidence/credible interval), ideally using structured tables or plots.	Results and Supplementary Table 4-10
Results of syntheses	18	Summarize and/or present the charting results as they relate to the review questions and objectives.	Results
**Discussion**			
Discussion	18a	Provide a general interpretation of the results in the context of other evidence.	Discussion
	18b	Discuss any limitations of the evidence included in the review.	Discussion
	18c	Discuss any limitations of the review processes used.	Discussion
	18d	Discuss implications of the results for practice, policy, and future research.	Discussion
**Other information**			
Registration and protocol	19	Provide registration information for the review, including register name and registration number, or state that the review was not registered.	N/A
Support	20	Describe sources of financial or non-financial support for the review, and the role of the funders or sponsors in the review.	Acknowledgements
Competing interests	21	Declare any competing interests of review authors.	Competing Interests
Availability of data, code, and other materials	22	Report which of the following are publicly available and where they can be found: template data collection forms; data extracted from included studies; data used for all analyses; analytic code; any other materials used in the review.	Availability of Data, Code, and Other Materials

Supplementary Table 2: Search Strategy (list of Queries used to search various databases)

Database	Query
PubMed	(("Visual Analytics"[Title/Abstract] OR "visual analytic*"[Title/Abstract] OR "Data Visualization"[Title/Abstract] OR "visual annotation"[Title/Abstract] OR "Image Visualization"[Title/Abstract] OR "Interactive Visualization"[Title/Abstract] OR "Visual Data"[Title/Abstract] OR "Information Visualization"[Title/Abstract] OR "Graphical Data Representation"[Title/Abstract] OR "Visual Representation"[Title/Abstract]) AND ("Histopathology"[Title/Abstract] OR "histopatholog*"[Title/Abstract] OR "Pathological"[Title/Abstract] OR "Tissue Analysis"[Title/Abstract] OR "Microscopic Imaging"[Title/Abstract] OR "Digital Pathology"[Title/Abstract] OR "Histological Image"[Title/Abstract] OR "Histopathology Image"[Title/Abstract] OR "Pathology Imaging"[Title/Abstract] OR "Tissue Imaging"[Title/Abstract] OR "Slide Imaging"[Title/Abstract] OR "Biopsy Analysis"[Title/Abstract])) AND (2000:2024[pdat])<br><br>Results: 98
IEEE Xplore	("All Metadata":"Visual Analytics" OR "All Metadata":"Visual Analytic*" OR "All Metadata":"Data Visualization" OR "All Metadata":"visual annotation" OR "All Metadata":"Image Visualization" OR "All Metadata":"Interactive Visualization" OR "All Metadata":"Visual Data" OR "All Metadata":"Information Visualization" OR "All Metadata":"Graphical Data Representation" OR "All Metadata":"Visual Representation") AND ("All Metadata":"Histopathology" OR "All Metadata":"Histopatholog*" OR "All Metadata":"Pathological" OR "All Metadata":"Tissue Analysis" OR "All Metadata":"Microscopic Imaging" OR "All Metadata":"Digital Pathology" OR "All Metadata":"Histological Image" OR "All Metadata":"Histopathology Image" OR "All Metadata":"Pathology Imaging" OR "All Metadata":"Tissue Imaging" OR "All Metadata":"Slide Imaging" OR "All Metadata":"Biopsy Analysis")<br>Filters Applied: 2000 - 2024<br>Results: 115 studies
Scopus	TITLE-ABS-KEY ("Visual Analytics" OR "Visual Analytic*" OR "Data Visualization" OR "visual annotation" OR "Image Visualization" OR "Interactive Visualization" OR "Visual Data" OR "Information Visualization" OR "Graphical Data Representation" OR "Visual Representation") AND TITLE-ABS-KEY ("Histopathology" OR "Histopatholog*" OR "Pathological" OR "Tissue Analysis" OR "Microscopic Imaging" OR "Digital Pathology" OR "Histological Image" OR "Histopathology Image" OR "Pathology Imaging" OR "Tissue Imaging" OR "Slide Imaging" OR "Biopsy Analysis")<br><br>Result: 457
ACM	[[All: "visual analytics"] OR [All: "visual analytic*"] OR [All: "data visualization"] OR [All: "visual annotation"] OR [All: "image visualization"] OR [All: "interactive visualization"] OR [All: "visual data"] OR [All: "information visualization"] OR [All: "graphical data representation"] OR [All: "visual representation"]] AND [[All: "histopathology"] OR [All: "histopatholog*"] OR [All: "pathological"] OR [All: "tissue analysis"] OR [All: "microscopic imaging"] OR [All: "digital pathology"] OR [All: "histological image"] OR [All: "histopathology image"] OR [All: "pathology imaging"] OR [All: "tissue imaging"] OR [All: "slide imaging"] OR [All: "biopsy analysis"]] AND [E-Publication Date: (01/01/2000 TO 12/31/2024)]<br><br>Result 332
ScinceDirect	Year: 2000-2024Title, abstract, keywords: ("Visual Analytics" OR "Data Visualization" OR "Image Visualization" OR "Interactive" OR "Visual Data" OR "visual annotation") AND ("Histopathology" OR "Digital Pathology"OR "Microscopic Imaging")<br><br>Result : 236
Google scholar	("Visual Analytics" OR "Data Visualization" OR "visual annotation" OR "Image Visualization" OR "Interactive" OR "Visual Data") AND ("Histopathology" OR "Digital Pathology" OR "Microscopic Imaging" OR "slide Image" OR "Tissue Analysis") Result : 200
Eurographics Digital Library	“Histopathology”<br>Result: 14
Total	1452

Supplementary Table 3: Data extraction form

**Concept**	**Definition**
Study Characteristics	
ID	Unique ID assigned to each study
Paper Title	Full title of the paper
Authors	List of authors, with first author highlighted
Publication Year	Year the study was published
Journal Name	Name of the journal where the study was published
Country	Country of the first author's institution
Citation	Number of citations the paper has received
Research Objective	Main aim or objective of the study
Study Design	Type of study design used
Methodology	Brief description of the methodology employed
Data Characteristics	
Data Source	Source of data for the study (e.g., public dataset, private collection)
Data Preprocessing	Methods used to preprocess the data
Type of Used Image	Specific type of histopathological images used
Histopathology Type	Type of tissue or pathology studied
Model and Algorithm	
Algorithm/Model	Specific type of AI model or algorithm used
Mathematical Contribution	Any novel mathematical approaches introduced
Functionality	Main functions or capabilities of the model
Evaluation	
Performance Metrics	Metrics used to evaluate the model's performance
Evaluation Methods	Methods used to validate the model (e.g., cross-validation)
Results	Summary of key results
Visualization and Interaction	
Visualization Techniques	Methods used to visualize results or data
Tool/Application Developed	Name or description of any tool or application created
User Interaction	Description of how users interact with the tool/application
Clinical Application	Potential or demonstrated clinical uses
Reproducibility and Reporting	
Open-Source	Whether the tool/code is open-source (Yes/No)
Development level	Classification of the tool (e.g., standalone, framework)
Solution name	Any nickname or shorthand used for the tool
Diseases	Specific disorder or disease studied, if applicable
Strengths and Limitations	
Strengths	Key strengths or advantages of the approach
Limitations	Main limitations or challenges identified

Supplementary Table 4: Study's Characteristics

ID	Year	Journal	Country	Citation	Objective	Design	Methodology
S1	2022	Medical Image Analysis	China	28	Propose hybrid self-supervised visual representation learning for H&E-stained images	Experimental	Hybrid approach with generative and discriminative learning, cross-stain prediction, and contrastive learning
S2	2021	Pacific Symposium on Biocomputing	USA	57	Develop modeling framework integrating GNNs and TDA for colon cancer staging	Experimental	Patch-level CNN embeddings, GNN with message passing, Mapper for feature extraction, logistic regression
S3	2022	Nature Biomedical Engineering	USA	17	Describe ecosystem of software for tissue image analysis	Perspective	Minerva software for interactive guides integrating multi-omic and tissue-atlas features
S4	2024	Computers in Biology and Medicine	Sweden	1	Develop web-based tool for annotating histopathology WSIs	Experimental	Self-supervised learning, seeded iterative clustering, CRF for annotation
S5	2005	Human Pathology	Finland	112	Evaluate validity of virtual microscopy for Gleason grading	Experimental	Digitized glass slides, interobserver variability assessed, agreement analysis using weighted coefficients
S6	2011	Computerized Medical Imaging and Graphics	USA	50	Develop HistoStitcher© for reassembly of histology fragments	Experimental	GUI for reassembling histological sections, image transformation, parameterized linear coordinate transformation
S7	2017	Cancer Research	USA	157	Develop Digital Slide Archive (DSA) for managing histologic images	Development	Built on Girder, supports various WSI viewers, integration with HistomicsTK for image analysis
S8	2023	IEEE Transactions on Visualization and Computer Graphics	USA	12	Develop visual analytics system for analyzing cell interaction patterns	Experimental	Domain-specific workflow, scalable computational pipeline, case studies with biomedical experts
S9	2020	PLOS Computational Biology	Switzerland	126	Develop framework for objective WSI quantification integrating machine learning	Experimental	Tile-based map-reduce approach, pixel classification, object segmentation, distributed computing with Apache Spark
S10	2017	Scientific Reports	UK	5077	Develop bioimage analysis platform for WSI data	Experimental	Analyzed image sets from colon cancer patients, focused on IHC staining patterns
S11	2021	IEEE Access	Norway	20	Develop FastPathology platform for deep learning-based research in digital pathology	Experimental	WSI reading, GPU-based rendering, tissue segmentation, multiple inference engines, user-friendly GUI
S12	2023	Journal of Pathology Informatics	USA	15	Develop deep learning model for ovarian cancer segmentation	Experimental	DIaL with pretrained segmentation model, ResNet-182 for BRCA prediction, PyTorch implementation, Tesla V100 GPU
S13	2022	Cancer Cell	USA	200	Develop deep learning model for joint image-omic prognostic models	Experimental	Multimodal algorithm with H&E WSIs and molecular profile features, trained on 6,592 WSIs from 5,720 samples
S14	2024	Medical Image Analysis	UK	4	Develop SynCLay for generating realistic histology images	Experimental	Utilizes GANs, HoVer-Net for nuclear segmentation, graph convolutional networks, TheCoT for image generation
S15	2019	Journal of Pathology Informatics	Switzerland	46	Develop retrieval system for content-based visual search in WSIs	Experimental	Integrated retrieval system with WSI viewer, multimodal approach with text and image features
S16	2023	Medical Image Analysis	China	13	Develop weakly-supervised method for nuclei segmentation	Experimental	Coarse pixel-level labels, co-training strategy, self-supervised visual representation learning
S17	2021	Scientific Reports	India	139	Develop deep learning framework for histopathology tissue analysis	Experimental	Preprocessing-training-inference pipeline, ensemble segmentation models, patch-based training and inference
S18	2023	Computer Graphics Forum	Czech	0	Develop open-source WSI viewer with annotation tools	Experimental	Modular, protocol-agnostic architecture, efficient rendering with WebGL, neural network inspector plugin
S19	2020	Medical Image Analysis	UK	132	Develop CNN-based framework for interactive annotation of microscopic objects	Experimental	NuClick framework with encoder-decoder network, minimal user interaction, hybrid weighted loss function
S20	2019	JCO Clinical Cancer Informatics	USA	260	Develop HistoQC tool for automated assessment of digital pathology slides	Experimental	Image metrics, supervised classifiers, HTML5-based interface for real-time visualization and data filtering
S21	2023	CVPR	India	5	Improve cell detection and counting with Deep Guided Posterior Regularization	Experimental	DE GPR framework with explicit and implicit features, Gaussian Mixture Model, supervised contrastive loss

S22	2023	Nature Communications	USA	11	Develop SLIM, interoperable web-based slide microscopy viewer	Experimental	Single-page web application, DICOM standard for image communication, WebGL for GPU-accelerated rendering
S23	2023	IEEE Transactions on Biomedical Engineering	USA	10	Develop Omni-Seg for multi-tissue segmentation in renal pathology	Experimental	Residual U-Net backbone, class-aware and scale-aware controller, semi-supervised consistency regularization
S24	2021	Journal of Pathology Informatics	USA	5	Increase efficiency of digital slide visualization with FlexTileSource	Experimental	FlexTileSource for OpenSeadragon, performance comparison with traditional DziTileSource
S25	2021	Journal of Pathology Informatics	USA	12	Develop system for diagnosis screening of ovarian tumors using digital pathology	Experimental	Informatics system with Groovy scripts, QuPath, watershed segmentation, machine learning for classification
S26	2020	Computer Methods and Programs in Biomedicine	USA	26	Develop diagnostic system for thyroid frozen sections	Experimental	Feature extraction and fusion using deep neural networks, region classification and retrieval on classifiers
S27	2023	Journal of Pathology Informatics	UK	1	Develop Comparative Pathology Workbench for collaborative analysis	Developmental	Web-based visual analytics platform, Django backend, integration with tools like QuPath and ImageJ
S28	2021	Kidney International Reports	Japan	16	Develop unsupervised approach for assessing kidney biopsy images	Experimental	NASNet pretrained feature extraction, UMAP, GMM clustering, visualization with Score-CAM and Grad-CAM
S29	2017	Cancer Research	USA	60	Develop containerized software system for WSI feature management	Experimental	Docker containers for image analysis, data management, web applications for visualization
S30	2016	BioMedical Engineering OnLine	China	111	Develop statistical model for quantifying histological biomarkers in IHC images	Experimental	Statistical model for color detection, maximum likelihood classifier, implemented in ImageJ plugin
S31	2017	Scientific Reports	USA	68	Develop interactive machine-learning system for histologic object classification	Experimental	HistomicsML with active learning, web-based interface for gigapixel images, classification accuracy improvement
S32	2022	Physics in Medicine & Biology	USA	3	Develop web-based platform for WSI visualization, annotation, and analysis	Development	Ruby on Rails, PostgreSQL, OpenSeadragon, ThreeJS, WebGL, multi-resolution image registration, API for algorithms
S33	2019	ICBBE	Thailand	3	Develop RUN-ONCO platform for managing cancer precision medicine data	Experimental	Hierarchical clustering, K-means clustering, REST API, integration with public databases
S34	2022	IEEE Transactions on Visualization and Computer Graphics	USA	17	Develop scalable system for WSI exploration and annotation	Experimental	Image exploration, spatially referenced single-cell expressions, finding similar regions, annotation
S35	2019	International Journal of Data Mining and Bioinformatics	China	4	Present OpenHI for collaborative histopathological image annotation	Development	SLIC Superpixel algorithm, server-end module with MySQL, JavaScript-based GUI for WSI annotation
S36	2024	BMC Medical Informatics and Decision Making	Portugal	0	Develop cloud-based system for multimodal histological image registration	Experimental	Django framework, Docker deployment, multiple plugins for image registration
S37	2024	Medical Image Analysis	China	1	Propose cross-modal retrieval framework for WSIs and diagnostic reports	Experimental	Anchor-based WSI encoder, prompt-based text encoder, multivariate cross-modal loss function
S38	2024	Nature Communications	South Korea	0	Develop PathoRiCH for predicting platinum-based treatment responses in ovarian cancer	Experimental	MIL models for histopathological images, trained on in-house and external cohorts, combined with molecular biomarkers
S39	2024	BMC Bioinformatics	USA	11	Develop Slideflow library for digital histopathology	Experimental	Python package with PyTorch and Tensorflow, tools for WSI processing, stain normalization, whole-slide classification
S40	2024	Proc SPIE Int Soc Opt Eng	USA	0	Develop automated computational renal pathology suite	Experimental	Detectron2 library for panoptic segmentation, trained on 190 WSIs, segmentation masks saved as JSON annotations
S41	2022	Communications Medicine	USA	23	Develop Histo-Cloud for WSI segmentation with user-friendly interface	Experimental	CNNs for WSI segmentation, integration with Deeplab V3+ and Digital Slide Archive for cloud-based analysis
S42	2022	Medical Image Analysis	Canada	99	Leverage unlabeled data for histopathology image analysis	Experimental	Self-supervised pretext task, teacher-student semi-supervised consistency paradigm
S43	2014	PLoS ONE	India	1097	Develop open source plugin for automated IHC image scoring	Experimental	Digital IHC image analysis with color deconvolution, computerized pixel profiling

S44	2020	Molecular Biology of the Cell	Hungary	42	Develop ImageJ plugin for semiautomatic annotation of cellular compartments	Experimental	U-Net-based presegmentation, manual refinement for accurate annotation
S45	2021	IEEE Transactions on Medical Imaging	USA	52	Develop STRAP for domain-agnostic visual representations in computational pathology	Experimental	AdaIN style transfer, MobileNetv2 model, stochastic gradient descent with momentum
S46	2020	Nature Biomedical Engineering	USA	1002	Develop CLAM for computational pathology on WSIs	Experimental	Attention-based learning, clustering, CNNs
S47	2022	Nature Medicine	USA	65	Develop CRANE for computational pathology on WSIs	Experimental	Attention-based learning, clustering, CNNs
S48	2016	Bioinformatics	Belgium	183	Develop Cytomine for collaborative analysis and visualization of large-scale imaging data	Experimental	Web development methodologies, machine learning, Cytomine-Core, Cytomine-IMS, Cytomine-WebUI, Cytomine-DataMining
S49	2019	Nature Methods	USA	1710	Present ilastik, interactive tool for machine-learning-based bioimage analysis	Experimental	Supervised machine learning, Random Forest, pixel classification, object classification, tracking workflows
S50	2023	Arxiv	Spain	2	Develop open-source web platform for digital histology image annotation and analysis	Experimental	CNNs for tumor region identification, image annotation tools, visual analysis
S51	2006	Genome Biology	USA	5595	Develop open-source software for high-throughput cell image analysis	Experimental	CellProfiler software, pipeline of image processing modules for cell feature measurement
S52	2012	Nature Methods	France	1550	Develop collaborative bioimage informatics platform with visual programming framework	Experimental	Platform for developing and sharing image analysis protocols, centralized repository for plugins and workflows

Supplementary Table 5: Data Characteristics

ID	Data Source	Data Preprocessing	Histopathology Type	Image Type
S1	NCT-CRC-HE-100K, TCGA-HCC, Xiangya glioma dataset	Stain separation (Vahadane's method), normalization, stain vector perturbation	Colorectal cancer, hepatocellular carcinoma (HCC), glioma	Microscopy Images and WSI
S2	Colon and lymph node resection slides from Dartmouth Hitchcock Medical Center	PathFlowAI pipeline: extraction and preprocessing into NPY format	Colon cancer, lymph node tissues	WSI
S3	Human Cell Atlas, Human Tumor Atlas Network	Image annotation and detailed marking up	General; immune cell types, tumor boundaries, stromal cells, multiplexed immunofluorescence	Multiplexed Tissue Imaging and WSI
S4	CAMELYON17, DigestPath	Patch extraction, color normalization using Stainlib	Various, including breast cancer, digestive system	WSI
S5	Tampere University Hospital, Finland	Hematoxylin and eosin staining, image sharpening, stitching, wavelet compression	Prostatic needle biopsies	Digitized Histological Sections and WSI
S6	Digital images of histological sections from prostate specimens	Not mentioned	Prostate histology sections	Digitized Histological Sections and WSI
S7	Cancer Digital Slide Archive (CDSA), The Cancer Genome Atlas (TCGA)	Not explicitly described	Frozen and formalin-fixed paraffin-embedded (FFPE) tissue samples	WSI
S8	Multiplexed tissue images (CyCIF)	OME standard for microscopy image data, MCMICRO image processing pipeline	Histopathology images from multiplexed tissue imaging techniques (CyCIF)	Multiplexed Tissue Imaging
S9	Real-world datasets (IPF quantification, IENFD quantification, glomeruli detection)	Tile-based processing, feature extraction using structure-size parameter	Lung tissue (IPF), human skin biopsies (IENFD), kidney tissue (glomeruli)	WSI
S10	Surgical resection specimens (660 patients, Northern Ireland Biobank)	TMA dearraying, manual refinement, stain vector estimation using color deconvolution	Colon adenocarcinoma, tissue microarrays (TMAs), H&E staining, immunohistochemistry (IHC)	WSI, Microscopy Images, IHC, Multiplexed
S11	BACH, Breast Cancer Subtypes Project, PanNuke	Image normalization, tissue segmentation	Breast cancer tissue, multi-organ pan-cancer	WSI
S12	609 high-grade serous ovarian cancer cases (Memorial Sloan Kettering Cancer Center)	Image normalization, background removal	High-grade serous ovarian cancer (HGSOC)	Digitized Histological Sections and WSI
S13	TCGA	Image normalization, background removal	Various cancer types including breast, colorectal, lung, pancreatic, uterine carcinoma	Digitized Histological Sections and WSI
S14	CONIC, PanNuke	Nuclei objects extraction, centroid location, bounding box collection using OpenCV	Colon histology images	WSI
S15	ContextVision AB, PubMed Central dataset	Pyramidal format conversion using VIPS, patch extraction at different magnifications	Prostate cancer biopsies	WSI
S16	MoNuSeg, Computational Precision Medicine (CPM)	Images cropped to patches, data augmentation	H&E stained pathology images of different organs including breast, brain, bladder, colon, kidney, lung, prostate	WSI
S17	CAMELYON, DigestPath, PAIP	Tissue mask generation, HSV color space transformation, Otsu's thresholding, morphological operations, data augmentation	Breast cancer metastases, colon cancer, liver cancer	Digitized Histological Sections and WSI

S18	WSIs from various histopathology datasets	Configurable visualization options, extensible plugins, data handling	Various tissue types including those in cancer studies	Digitized Histological Sections and WSI
S19	Gland Segmentation dataset (GlaS), GRAG datasets, MonuSeg, CPM datasets	Image augmentations including flip, brightness, contrast, sharpness adjustments, Gaussian noise addition	Histopathology images from various sources including H&E, IHC, cytology images (Pap Smear)	Light Microscopy Images
S20	TCGA breast cancer cohort	N/A	Hematoxylin and eosin bright-field microscopy images	WSI
S21	MuCeD, CoNSeP, MoNuSac	Image slicing, resizing, normalization	Human duodenum biopsies (MuCeD), colorectal adenocarcinoma (CoNSeP), multi-organ nuclei (MoNuSac)	WSI, Microscopy Images
S22	TCGA, CPTAC, HTAN projects	TIFF to DICOM format conversion, metadata harmonization	Brightfield microscopy (H&E stained), fluorescence microscopy (immunostained)	WSI, Microscopy Images
S23	1,751 ROIs from 459 WSIs, NEPTUNE study	Tiling of large images, downsampling	Renal pathology images (human and mouse kidneys)	WSI, Microscopy Images
S24	8104 WSIs from routine clinical diagnostics	Not explicitly mentioned	General whole-slide images	WSI
S25	30 cases from institutional pathology system database	Manual annotation, cell segmentation using watershed algorithm	Serous borderline ovarian tumor (SBOT), high-grade serous ovarian cancer (HGSOC)	WSI
S26	Frozen sections of thyroid from Peking Union Medical College Hospital	Patches cropped from ROI, kept if ≥75% pixels inside ROI	Thyroid frozen sections	WSI
S27	Image Data Resource (IDR), OMERO, Single Cell Expression Atlas (SCEA)	Not explicitly mentioned, CPW stores URL references to images on external resources	Various types including H&E stained sections, histochemical special stains, immunohistochemical stains, immunofluorescence stains, in situ RNA/DNA hybridisation stains	WSI
S28	Kidney biopsy specimens (68 patients, Kyoto University Hospital)	H&E staining normalization, white area removal, image resizing, patch extraction	Kidney biopsy images, specifically for IgA nephropathy	WSI, Microscopy Images
S29	SEER registries, other cancer research datasets	Image normalization, segmentation, feature extraction	Various cancer types including prostate, NSCLC, breast cancer, lymphoma, colorectal cancer, melanoma	WSI
S30	Human oesophageal cancer, colon cancer, liver cirrhosis biopsy slides	Image normalization, histogram quantization	Human oesophageal cancer, colon cancer, liver cirrhosis	IHC Images, WSI
S31	TCGA Lower Grade Glioma (LGG) project	H&E color standard normalization, tissue pixels masked, cell nuclei highlighted using color deconvolution	Lower-grade glioma (LGG)	IHC Images, WSI
S32	User-uploaded histopathology WSIs	Conversion to Deep Zoom Image (DZI) pyramidal tiling format	Supports various types including H&E, IHC, Masson's trichrome	WSI, Microscopy Images
S33	Clinical records, biospecimens, -omics data from patients, public databases (STRING, OncoKB)	Data integration from various sources	Various cancer types including colon, lung, ovarian cancers	WSI
S34	Multiplexed tissue imaging data generated with CyCIF	High-resolution optical microscopes, non-rigid registration, cell segmentation, feature extraction	Lung and colorectal cancers	Multiplexed Tissue Imaging
S35	WSIs from TCGA	Pre-segmented using SLIC Superpixel algorithm	Designed for various oncological types	WSI
S36	Not explicitly mentioned	Image normalization, background removal, multi-resolution pyramid creation	Not specified	WSI, Multimodal Images
S37	In-house gastric dataset, public GastricADC dataset	Threshold segmentation, patch sampling, feature extraction using pre-trained CNN model	Gastric tissue	WSI

S38	In-house cohort (Yonsei Severance Hospital), TCGA-OV, Samsung Medical Center	Automated cancer segmentation using UNetPlusPlus, patch extraction at different magnifications, filtering non-cancerous areas	High-grade serous ovarian cancer (HGSOC)	WSI
S39	TCGA, internal datasets from University of Chicago	Image normalization, background removal, tile extraction, stain normalization	Head and neck squamous cell carcinoma (HPV status prediction), other types like thyroid, breast, lung cancer	WSI, IHC, Microscopy Images
S40	Kidney Precision Medicine Project (KPMP), Human BioMolecular Atlas Program (HuBMAP)	Image normalization, cropping large WSIs into small patches	Kidney-specific histopathology including diabetic nephropathy, lupus nephritis, transplant biopsies	WSI
S41	Public repositories, institutes including UC Davis, Johns Hopkins University	Image normalization, background removal, multi-resolution patch extraction	Renal histopathology including various renal disease pathologies and biopsy types	WSI
S42	BreastPathQ, Camelyon16, Kather multi-class datasets	Image normalization, geometric transformations, color perturbations	Tumor metastasis detection, tissue type classification, tumor cellularity quantification	WSI
S43	Human Protein Atlas, tumor tissue repository at TMC-ACTREC	Color deconvolution, histogram profiling	Various cancers including breast, colon, cervical, liver, lung, melanoma, thyroid, oral, ovarian cancer	Microscopy Images, IHC
S44	Microscopy images of various experimental conditions	Image normalization, region growing for segmentation initialization	Not applicable (no specific histopathology type mentioned)	Microscopy Images, IHC
S45	TCGA, CAMELYON17-WILDS	Image normalization, style transfer, random resizing	Colorectal cancer, breast cancer metastases	WSI
S46	TCGA, Camelyon16, Camelyon17	Segmentation, patch extraction, feature extraction using pretrained CNNs	Renal cell carcinoma, non-small cell lung cancer, breast cancer lymph node metastasis	WSI
S47	Brigham and Women's Hospital, Ege University, Bern University Hospital	Segmentation, patch extraction, feature extraction using pretrained CNNs	Endomyocardial biopsies	WSI
S48	Various bio(medical) imaging datasets including whole-slide virtual microscopy, automated volume electron microscopy	Image normalization, background removal, conversion to pyramidal format during upload	Various types including brightfield cytology, histology images, H&E stained digital slides, HDAB stained IHC slides	WSI, IHC, Microscopy Images
S49	Various biological image datasets	Image segmentation, boundary probability map creation, superpixel computation	Tissue images with membrane staining, light microscopy tissue images	WSI, IHC, Microscopy Images
S50	Private databases from University Clinic Hospital of Valencia, San Cecilio University Hospital	Images downsampled, divided into patches, pre-processed using Otsu method for tissue detection	Various neoplasms including leiomyomas, leiomyosarcomas, dermatofibromas, spindle cell melanomas, fibroxanthomas, squamous cell carcinoma	WSI
S51	Various biological image sets (RNAi screens, human, Drosophila, yeast cells)	Image normalization, illumination correction, object identification, segmentation, measurement	Not applicable	Microscopy Images
S52	Various bioimage datasets contributed by the community	Not explicitly mentioned	Not applicable	WSI, IHC, Microscopy Images, Multiplexed

Supplementary Table 6: Algorithm Characteristics

ID	Algorithm/Model	Mathematical Contribution	Functionality
S1	ResNet-based autoencoders, Siamese networks, MLPs, contrastive learning, cross-stain prediction, stain vector perturbation, Adam optimizer, early stopping	Combines generative (cross-stain prediction) and discriminative (contrastive learning) methods, using stain separation and vector perturbation to enhance learning	Feature extraction, tissue classification, cancer prognosis and subtyping, image reconstruction, unsupervised representation learning
S2	ResNet-50 CNN, GNN with message passing, Mapper for topological summarization, logistic regression	Integrates Topological Data Analysis (TDA) techniques, specifically Mapper, to summarize GNN embeddings into interpretable topological structures	Feature extraction, image segmentation, contextualization of patches, topological summarization, identification of ROI
S3	OpenSeadragon, client-side JavaScript, JSON, YAML	Leveraging OpenSeadragon for spatial and multi-channel image exploration, interoperability using OME-TIFF standards	Feature extraction, classification, segmentation, interactive exploration, narrative guidance, quantitative analysis integration
S4	SimCLR, ResNet18, Conditional Random Fields (CRF)	Introduced seeded iterative clustering, CRF post-processing on UMAP features	Interactive annotation, feature space gating, seeded iterative clustering
S5	Not applicable	Not applicable	Interactive web site with digitized prostatic needle biopsies, Gleason score assignment and comparison
S6	HistoStitcher©, linear transformations, Procrustes analysis, cubic spline interpolation	Novel approach for determining optimal linear transformation based on user-defined control points, incorporating scaling and reflection constraints	Interactive selection of control points, transformation constraints, optimal transformations, previewing stitched images, saving/loading transformations
S7	HistomicsTK, image analysis library for DSA	Utilizes image analysis algorithms and libraries like HistomicsTK, which rely on mathematical concepts	Managing and organizing large collections of histopathology images, annotation, visualization, handling WSIs, image analysis tasks
S8	Computational neighborhood quantification, ball tree, partition-based clustering, permutation testing	Methods for neighborhood quantification and pattern analysis, including ball tree, EM algorithm for GMMs, permutation testing	Exploring spatial neighborhoods, interactive clustering, visually querying patterns, comparing patterns within and across datasets
S9	Orbit, linear SVM, watershed algorithm, Mumford-Shah functional-based segmentation, DeepLab v2 (ResNet-101)	Concept of structure-size for context-based structure classification, tile-based map-reduce approach for parallel processing and distributed computing	Pixel classification, object segmentation, ROI definition, annotation creation, exclusion model training, mask generation, integration of external tools
S10	Cell detection algorithms, random trees classifiers, color deconvolution, SLIC, Haralick texture features, Gaussian weighted sum of features	Color deconvolution, Haralick texture features, Gaussian weighted sum of features, random trees classifiers	WSI viewer, annotation tools, automated segmentation, object classification, batch processing, data mining, visualization, data exchange
S11	CNNs (MobileNetV2, U-Net, TinyYOLOv3)	Tiled image pyramid approach with memory mapping and GPU-based rendering techniques	WSI visualization and navigation, CNN model deployment, patch-wise classification, segmentation, object detection, result visualization, data analysis and export
S12	Deep Interactive Learning (DIaL), DMMN, ResNet-182, stochastic gradient descent (SGD), Adam optimizer	DMMN with multi-encoder, multi-decoder, multi-concatenation architecture, SGD and Adam optimizer for training models	Feature extraction, classification, segmentation, BRCA mutation prediction, interactive exploration, anomaly detection, data augmentation, attention heatmaps, SHAP-styled plots, Kaplan-Meier curves
S13	Multimodal deep learning, AMIL, SNN, Kronecker Product, SHAP, Integrated Gradients	Kronecker Product for pairwise feature interactions, attention- and attribution-based interpretability for multimodal data	Feature extraction, classification, segmentation, interactive exploration, anomaly detection, data augmentation, attention-based interpretability, global and local interpretability
S14	GANs, HoVer-Net, graph convolutional networks, residual encoder–decoder network, TheCoT model	GANs for image synthesis, HoVer-Net for nuclear segmentation, graph convolutional networks for cellular layout processing, TheCoT for parameter-based image generation	Interactive synthesis of histology images, generation of nuclear segmentation masks, control over cellular layouts and image parameters, augmentation of limited datasets with synthetic images
S15	DenseNet-BC 121, Color and Edge Directivity Descriptor (CEDD)	N/A	WSI viewer, visual feature selection, content-based image retrieval (CBIR), text-based retrieval

S16	SC-Net (ResUNet architecture, colorization network)	Two U-Nets: segmentation network with cross-entropy loss, co-training loss using EMA, colorization network reconstructs H&E image	Nuclei segmentation, visualization of results including heatmaps and scatter plots
S17	Ensemble segmentation models (DenseNet-121, Inception-ResNet-V2, DeeplabV3Plus)	Dice loss function for segmentation	Tumor segmentation, lymph node metastases classification, viable tumor burden estimation, uncertainty estimation
S18	WebGL-based rendering, neural network inspector plugin	Efficient WebGL-based rendering, domain-specific image processing methods, neural network inspector plugin for feature maps visualization	Visualization of high-dimensional feature spaces, annotation tools, session sharing, storytelling, integration with AI models
S19	NuClick, encoder-decoder network architecture with multi-scale and residual blocks	Multi-scale convolutional blocks, weighted hybrid loss function, morphological skeleton for guiding signals	Interactive segmentation of nuclei, cells, and glands, robust to variations in user input, segmentation of objects of different scales, hybrid weighted loss function, post-processing step for refining segmentation masks
S20	HistoQC, color histograms, brightness and contrast analysis, edge detectors, supervised classifiers	Combines existing image processing techniques and machine learning algorithms in a novel way	Artifact identification, batch effect detection, region selection, outlier detection
S21	Yolov5, Faster-RCNN, EfficientDet, D E GPR, ResNet18, Gaussian Mixture Model (GMM)	Deep Guided Posterior Regularization (D E GPR), incorporates expert knowledge and data-driven features, KL divergence loss function	Enhances object detection models for cell detection and counting, uses explicit and implicit features, pre-training step for implicit feature encoder
S22	DICOM Profile Connection Space Transformation, DICOM VOI LUT Transformation, DICOM Advanced Blending Transformation	Affine transformations for mapping between slide coordinate systems and image pixel matrices	Interactive visualization, annotation, display of image analysis results, color management, multi-resolution image navigation
S23	Residual U-Net, Class-aware Controller, Scale-aware Controller, dynamic Head, Feature fusion Block, Semi-supervised Consistency Regularization	Scale-aware controller for multi-scale segmentation, semi-supervised constraints to enforce similar embedding of augmented images	Multi-label segmentation, multi-scale segmentation, scale-aware knowledge integration, semi-supervised learning, spatial relationship modeling
S24	Custom FlexTileSource for OSD	No specific mathematical contribution claimed	Efficient tile serving from native WSI pyramid levels, flexible specification of pyramid level dimensions and tile sizes
S25	SVM, LASSO, Gaussian-based KDE	Gaussian-based KDE for multi-scale characterization of tumor-stroma interactions	Cell detection, classification, feature extraction, tissue-level classification
S26	VGG16bn, InceptionV3, ResNet50, power mean with exponent p, decision tree, SVM, random forest, multilayer perceptron, supervised hashing methods	Power mean with exponent p for flexible patch feature fusion	ROI classification and retrieval, deep learning feature extraction and fusion
S27	Integration with QuPath and ImageJ	No specific mathematical contributions mentioned	Interactive comparison of images, ROI selection and annotation, shared workspace with discussion threads, integration with external tools and data sources, grid layout for arranging images and analysis results
S28	NASNet, UMAP, Gaussian Mixture Models, fine-tuned NASNet, convolutional autoencoder	No specific mathematical contribution claimed	Feature extraction, unsupervised clustering, classification, visualization of decision rationale
S29	Level set-based nuclear segmentation, Docker containers	Robust image analysis algorithms, including nuclear segmentation and feature extraction techniques	Feature extraction, classification, segmentation, interactive visualization, exploration of histopathology images
S30	Statistical color detection model, maximum likelihood classifier	Statistical modeling of color detection, removal of luminance channel, quantization in chromaticity spaces	Feature extraction, classification of stained pixels, automated and interactive analysis in ImageJ plugin
S31	Random Forests, Active Learning, confidence measure, Ripley's K-function, Principal Curve	Active learning methods, confidence measure based on tree votes, spatial clustering quantification with Ripley's K-function, hypertrophy score calculation with Principal Curve	Visualization and interaction with gigapixel images, training and evaluation of classification rules, reviewing labeled data, interactive exploration of image analysis boundaries, visualizing prediction confidences, active learning for improved training efficiency

S32	Multi-resolution registration, deep learning and conventional methods for cell detection, segmentation, quantification	Dynamic multi-resolution registration method for 3D reconstruction of serial WSIs	WSI upload, 2D/3D visualization, annotation, analysis, result visualization, sharing
S33	Hierarchical clustering, K-means clustering, variant call analysis, integrative analysis of gene expression profiles	Hierarchical clustering, K-means clustering, integration of multiple -omics data sources, REST API for data access and analysis	Integrative data analysis, visualization plugins, variant call comparison, clustering of gene expression profiles, visualization of protein-protein interaction networks
S34	Lens-centric focus+context technique, interactive exploration of multi-channel images and linked multivariate data	Not mentioned	Finding, magnifying, quantifying, organizing ROIs, interactive panning and zooming, image channel selection, color adjustments, descriptive statistics, comparison of image features, cell types, spatial arrangements
S35	SLIC Superpixel Algorithm, OpenSlide, OpenCV, Flask, MySQL, OpenSeadragon, JQuery	Calculation of the number of superpixels based on desired average sub-region size and total resolution of WSI, virtual magnification calculation	Annotation with pixel-level precision, online collaborative annotation, sub-region selection and grading, visualizing WSIs with smooth zooming and panning capabilities, virtual magnification indicator
S36	ORB, SIFT, FLANN, K-Nearest Neighbors (KNN)	Incorporates various image registration techniques and algorithms	Feature extraction, image registration, visualization, transformation of segmented annotations
S37	Anchor-based attention module, prompt-based text representation learning, CLIP, hierarchical kernel attention module	Defines anchor-based and prompt-based representations for semantic alignment, introduces multivariate cross-modal loss functions	Image-to-image, image-to-text, text-to-image, text-to-text retrieval tasks
S38	Multiple instance learning (MIL) models, contrastive self-supervised learning with SimCLR, UNetPlusPlus, ResNet34, ResNet18	Spatial Transformer Networks (STN) to improve classification and segmentation, affine transformation matrices	Predicting treatment response categories in HGSOC, visualization of model decisions, identifying histologic features associated with treatment response
S39	Xception, CTransPath, CLAM, GANs (StyleGAN2, StyleGAN3), SimCLR	Efficient tile extraction, real-time stain normalization, self-supervised learning (SimCLR), multiple-instance learning (CLAM), uncertainty quantification, feature space analysis using UMAP	Feature extraction, classification, segmentation, interactive exploration, anomaly detection, data augmentation, real-time whole-slide visualization
S40	Detectron2 library, panoptic feature pyramidal architecture, customized for FTU segmentation and classification	Panoptic segmentation neural network, spatial transformer networks	Feature extraction, classification, segmentation, interactive exploration, anomaly detection, data augmentation, segmentation mask generation and visualization, user interaction for quality evaluation and editing
S41	DeepLab V3+, CNNs	Spatial transformer networks to improve classification and segmentation, affine transformation matrices	Feature extraction, classification, segmentation, interactive exploration, anomaly detection, data augmentation
S42	ResNet-18, self-supervised learning, semi-supervised learning, teacher-student consistency paradigm	Self-supervised pretext task (Resolution Sequence Prediction), semi-supervised consistency training paradigm	Feature extraction, classification, regression, prediction consistency
S43	Spectral deconvolution method, ImageJ plugin	Score assignment formula based on pixel intensity values	Automated scoring, pixel-by-pixel analysis, histogram profiling
S44	U-Net, region-growing algorithm	Integration of U-Net and active contour models for object segmentation	Annotation of cellular compartments, export of annotations in multiple formats
S45	AdaIN style transfer, MobileNetv2	STRAP for better domain generalization in computational pathology	Improves robustness to domain shifts, facilitates learning of domain-agnostic representations
S46	Attention-based pooling, clustering-constrained multiple instance learning (CLAM)	Attention-based pooling for weakly-supervised learning, clustering-constrained learning	Interpretable whole slide image analysis, heatmap generation, class-specific slide representation
S47	Attention-based pooling, clustering-constrained multiple instance learning (CLAM)	Attention-based pooling for weakly-supervised learning, clustering-constrained learning	Interpretable whole slide image analysis, heatmap generation, class-specific slide representation
S48	Supervised and unsupervised learning algorithms, semantic segmentation, object classification, landmark detection, content-based image retrieval	Extremely randomized trees, supervised classification algorithms, object classification, semantic segmentation, landmark detection	Feature extraction, classification, segmentation, interactive exploration, anomaly detection, data augmentation, remote collaboration

S49	Random Forest, watershed algorithm, CNNs for boundary probability map creation	Introduction of multicut problem-solving for image segmentation, use of Random Forest for edge classification	Semantic segmentation, object classification, counting, tracking, interactive machine learning
S50	CNNs, Teacher-Student model paradigm for pseudo-supervised learning	Teacher-student model paradigm for pseudo-supervised learning to enhance tumor region classification and segmentation accuracy	Annotation tools, automatic tumor region detection, heat-map predictions, crowdsourcing system for collaborative annotation
S51	Voronoi-based segmentation, propagation algorithm, Zernike shape features, Haralick and Gabor texture features	Implements various image analysis algorithms, Voronoi-based segmentation, propagation algorithm, Zernike shape features, Haralick and Gabor texture features	High-throughput image analysis, object identification, cell feature measurement, data export to spreadsheets and databases, compatibility with cluster computing for large image sets
S52	Various image analysis algorithms and methods, segmentation, tracking, feature extraction	Supports integration of new algorithms through a visual programming interface	High-throughput image analysis, protocol development, community contribution and sharing, centralized plugin repository, visual programming framework

Supplementary Table 7: Evalution Characteristics

ID	Evaluation Methods	Performance Metrics	Results
S1	5-fold and Monte Carlo cross-validation, comparison with baseline models, ablation studies	Accuracy, Harrell's concordance index (C-index), weighted F1-score, statistical significance (paired t-test)	Demonstrates improved visual representation extraction, achieving higher classification accuracies and better cancer prognosis compared to other methods
S2	10-fold cross-validation, comparison of AUROC and F1-scores before and after GNN application, logistic regression for clinical outcome prediction	AUROC (Area Under the Receiver Operating Characteristic Curve), F1-score	GNN models improved classification scores significantly after incorporating contextual information from patches. Topological features extracted using Mapper provided meaningful biological insights into tissue architecture and tumor invasion.
S3	Qualitative feedback on usability and capacity to enhance understanding among pathologists and non-specialists.	Not explicitly measured in terms of traditional metrics but discussed in terms of usability and potential for enhancing diagnostic accuracy.	Minerva effectively disseminates digital histology data, aiding interpretation, educational purposes, and research sharing.
S4	Experimental evaluation with pathologists, simulations	Dice-Sørensen Coefficient (DSC), Cohen's kappa coefficient	High accuracy in patch-level segmentation, competitive results on multiple benchmarks
S5	Intraobserver and interobserver agreement analysis using weighted j coefficients	Weighted j coefficients (for intraobserver and interobserver agreement)	Excellent intraobserver agreement between conventional glass slides and virtual slides (j = 0.73). Moderate to substantial interobserver agreement among expert uropathologists (weighted j coefficients ranging from 0.55 to 0.62).
S6	A 6-point scoring scheme, based on expert pathologist assessment, was used to evaluate the quality of stitched sections.	Performance evaluated by comparing the quality of stitched sections generated by HistoStitcher© to those generated by an expert pathologist using Photoshop. A 6-point scoring scheme was used to assess alignment and continuity of anatomical structures.	HistoStitcher© consistently produced better quality stitched sections with smoother alignment of adjacent boundaries and better continuity of internal structures compared to manual reconstruction using Photoshop.
S7	Evaluation based on architecture, features, and usability for managing and analyzing histopathology images.	No quantitative performance metrics for specific algorithms	DSA is a scalable and customizable open-source platform for managing, integrating, and analyzing histopathology images. A cost-effective alternative to commercial software.
S8	User feedback, think-aloud protocols, runtime performance analysis	Time for neighborhood computation, permutation testing, clustering, and search	Visinity effectively identified known spatial arrangements of immune cells in human tonsil specimens and uncovered novel white-blood cell networks and immune-tumor interactions in lung cancer specimens from genetically engineered mice.
S9	Manual assessment by pathologists, correlation factor analysis, statistical measures like Dice index and mean Dice object index	Accuracy, correlation factor, Dice index, visual evaluation by experts	Successful application of Orbit in IPF quantification, IENFD quantification, and glomeruli detection. Deep learning segmentation model (ResNet-101) achieved a mean Dice object index of 0.8754±0.0181 across various staining types and species.
S10	Statistical analysis: log-rank tests for survival analysis, visual assessment by pathologists	Kaplan-Meier curves, log-rank tests, cell counts, H-score, tumor stromal percentage	Significant associations found between biomarker scores and patient survival, supporting the use of QuPath for analyzing histopathology images in research.
S11	Memory usage, runtime, performance metrics (accuracy, precision, recall, F1-score)	Runtime (inference and processing), memory usage, accuracy, precision, recall, F1-score	FastPathology showed competitive memory usage and inference speed, excelling with OpenVINO CPU inference for certain use cases.
S12	Intersection-over-Union (IOU), recall, precision, area-under-curves (AUCs)	IOU, recall, precision, AUCs	Segmentation model achieved IOU of 0.74, recall of 0.86, precision of 0.84; BRCA prediction models achieved AUCs ranging between 0.49 and 0.67 on validation set and between 0.40 and 0.43 on testing set.
S13	c-Index, Kaplan-Meier analysis, log-rank test, bootstrap replicates, TIL quantification, visual assessment by pathologists	c-Index, Kaplan-Meier curves, log-rank test, dynamic AUC	Multimodal model achieved a c-Index of 0.644, outperforming unimodal models; improved patient risk stratification across 14 cancer types.
S14	Quantitative metrics (FID, SSIM), pathologist assessment, performance comparison with state-of-the-art models, ablation studies	Frechet Inception Distance (FID), Structural Similarity Index Measure (SSIM), pathologist realism scores	Generated synthetic images appear realistic, comparable to real images, effective in reducing data imbalance, improved performance in cellular composition prediction tasks.

S15	Quantitative evaluation using standard information retrieval metrics (MAP, GM-MAP, P10, P30), qualitative evaluation through user tests with pathologists	Mean average precision (MAP), geometric MAP, precision after ten patches retrieved (P10), precision after 30 patches retrieved (P30)	Deep learning features (DenseNet) outperformed handcrafted features (CEDD) in retrieval performance. Positive feedback from pathologists on the interface's ease of use and speed.
S16	Various metrics, including pixel accuracy, F1 score, Diceobj, AJI, DQ, SQ, and PQ	Pixel accuracy, F1 score, Diceobj, AJI, DQ, SQ, PQ	Outperforms other weakly-supervised methods and achieves competitive performance compared to fully-supervised methods. Co-training strategy and colorization proxy task contribute to improved performance.
S17	Challenge leaderboards, comparison with other published methods, performance metrics (e.g., Dice coefficient, Cohen's Kappa score), uncertainty analysis	Dice coefficient, Jaccard index, Cohen's Kappa score, tumor burden, uncertainty maps	Achieved state-of-the-art performance on CAMELYON, DigestPath, and PAIP challenges, ranking within the top 5 for each.
S18	Case studies with AI algorithm developers and research pathologists, qualitative feedback, performance evaluation	Performance evaluation through frame rates and user feedback from case studies	Demonstrated utility in developing and verifying AI algorithms, supporting efficient and accurate pathological analysis, and facilitating collaboration between researchers and clinicians.
S19	AJI, Dice, Hausdorff distance, DQ, SQ, F1-score, DiceObj, qualitative assessment	AJI, Dice coefficient, Hausdorff distance, DQ, SQ, F1-score, DiceObj	Achieved state-of-the-art performance on various datasets for nuclei, cells, and gland segmentation. Outperformed other interactive segmentation methods in terms of accuracy and robustness. Demonstrated generalizability and domain adaptability.
S20	Expert consensus: visual assessment by two experienced pathologists, quantitative metrics: accuracy (agreement between HistoQC and human experts)	Agreement between HistoQC and human pathologists, interobserver agreement between two pathologists	HistoQC achieved an agreement greater than 95% with human pathologists for identifying artifact-free regions. Interobserver agreement between pathologists was 96% for the 50 slides evaluated by both.
S21	Standard object detection metrics: precision, recall, mAP, MAE, MRE, Q-histology ratio	Precision, recall, mean average precision (mAP), mean absolute error (MAE), mean relative error (MRE), Q-histology ratio	D E GPR framework consistently improves detection and counting performance across all three datasets. Achieves significant gains in mAP (up to 9%) and reductions in MAE (up to 35%) for cell counting. Increases F1-score for celiac disease classification from 0.774 to 0.902.
S22	Participation in DICOM Connectathons, interoperability testing with commercial systems	Not explicitly stated, focus on interoperability and functionality	Successfully demonstrated interoperability with multiple DICOMweb server implementations and commercial slide scanners.
S23	Internal validation on publicly available dataset, external validation on murine kidney dataset, ablation studies, qualitative visual assessment of segmentation masks, quantitative evaluation using various metrics	Dice Similarity Coefficient (Dice), Hausdorff distance (HD), Mean Surface Distance (MSD), Pearson correlation, accuracy, precision, recall, F1-score, AUC	Superior segmentation performance compared to other methods, effective scale-aware controller and semi-supervised learning, good generalization ability when applying model trained on human kidney images to mouse kidney images without retraining.
S24	Comparison of FOV loading times between FlexTileSource and DziTileSource across different block and tile size configurations	Time to display field of view (FOV), normalized to standard FOV size of 1920x1080 px	FlexTileSource accelerated display of FOV by 67 ms overall, with 117 ms improvement when block size and tile size increased to 1024 px.
S25	Cross-validation, confusion matrix, ROC curve, bootstrap resampling for subject-level classification	Accuracy, confusion matrix, receiver operating characteristic (ROC) curve	Cell classification accuracy >90%. Tissue-level classification accuracy 90.5-90.7%. Subject-level classification accuracy 97%.
S26	k-fold cross-validation for classification performance, average of five splits for retrieval evaluation, top nine retrieved images used to calculate retrieval accuracy	Accuracy, precision, recall, F1-score for classification, mean average precision (MAP), precision, recall, accuracy for retrieval	System achieves 96.1% cross-validated classification accuracy and retrieval MAP of 0.972 on 345 thyroid frozen sections. Deep learning-based representation generation shows superior performance compared to GIST features.
S27	Case studies demonstrating CPW's use in dermatopathology, Crohn's disease research, and a coeliac disease audit, qualitative assessment of user feedback and usability	Not mentioned	Demonstrates usability through real-world examples, including a dermatopathology project, a Crohn's disease study, and a coeliac disease audit. Facilitates collaboration, review, and discussion among pathologists and researchers.
S28	Statistical analysis of relationships between histological scores and clinical variables, AUROC for classification performance	Area Under the Receiver Operating Characteristic curves (AUROCs), linear regression coefficients, p-values	Histological scores obtained by unsupervised clustering showed significant relationships with clinical variables in IgAN patients. 1-vs-1 weighted AUROC average: 0.921, 1-vs-rest weighted AUROC average: 0.918.

S29	Accuracy, AUC, precision, recall, F1-score, visual assessment by pathologists	Accuracy, precision, recall, F1-score, segmentation quality	High accuracy in segmentation and classification of tissue images, development of a comprehensive software infrastructure for tissue image analysis.
S30	ROC curves, comparison with manual ground truth, comparison with color deconvolution and CMYK models	Accuracy, true positive ratio, false positive ratio, AUROC	High accuracy in detecting DAB and PSR stains, robust across different datasets and users, higher accuracy compared to color deconvolution and CMYK models.
S31	AUC, c-index, manual validation by a neuropathologist	Area Under Curve (AUC), Concordance Index (c-index)	Developed an accurate classifier of vascular endothelial cell nuclei (VECN) using minimal training data. Quantified microvascular phenotypes using Hypertrophy Index (HI) and Clustering Index (CI), predicting survival of lower-grade glioma patients independent of grade and molecular subtype.
S32	Not explicitly described, appears to be based on functionality demonstration	Not explicitly stated for overall system	Successfully developed web platform with described functionalities.
S33	User testing, system performance evaluation, qualitative feedback from oncologists and researchers	Efficiency of data management, accuracy of variant call analysis, effectiveness of integrative analyses, user satisfaction	RUN-ONCO demonstrated efficient management and analysis of clinical and -omics data, enhanced visualization capabilities, and high user satisfaction.
S34	Use cases involving lung and colorectal cancer datasets, domain collaborator assessment through user studies and questionnaire	Not mentioned	Validated with domain experts and applied in case studies involving lung and colorectal cancers. Useful for characterizing tumor features, including cell types, states, and spatial organization in the tumor microenvironment.
S35	Annotation precision comparison, processing time measurement	Annotation quality, processing time	High annotation precision and decent processing speed, making OpenHI suitable for efficient large-scale annotation of WSIs.
S36	Visual assessment, evaluation using metrics like TRE, SSD, CC, MI	Accuracy, relative Target Registration Error (TRE), Sum of Squared Differences (SSD), Cross-correlation (CC), Mutual Information (MI)	Effective in registering high-resolution images of various types, including histological and non-histological images.
S37	Ablation studies, comparison with state-of-the-art methods, evaluation on in-house and public datasets	MAP@5, PIoU@10, P@3, PTACC	Proposed method achieved superior performance in retrieval tasks compared to state-of-the-art methods.
S38	Cross-validation, external validation, Kaplan-Meier survival analysis, Cox regression analysis, attention maps, clustering analysis	AUC-ROC, precision, recall, F1 score, Kaplan-Meier survival analysis	PathoRiCH showed significant predictive performance across internal and external cohorts, achieving better predictive performance than current molecular biomarkers.
S39	Cross-validation, external test set validation, accuracy, AUROC, AP, uncertainty quantification, visual assessment by pathologists	Accuracy, AUROC, average precision (AP)	High accuracy and AUROC in predicting HPV status in head and neck squamous cell carcinoma using weakly-supervised tile-based and multiple-instance learning models.
S40	Accuracy, AUC, precision, recall, F1-score, visual assessment by pathologists	Accuracy, precision, recall, F1-score	Accurate and precise segmentation results of kidney FTUs verified by nephropathologists.
S41	Accuracy, AUC, precision, recall, F1-score, visual assessment by pathologists	Accuracy, precision, recall, F1-score, Matthews correlation coefficient (MCC), Cohen's kappa, Intersection over Union (IoU)	High accuracy and robustness in segmenting glomeruli with an F-score of 0.97 and MCC of 0.97.
S42	Supervised fine-tuning, consistency training	Accuracy, Intra-class Correlation Coefficient (ICC), AUC	Improvements close to or outperforming other state-of-the-art methods in limited-label data regimes.
S43	Comparison with manual scoring by pathologists, kappa statistics	Match percentage with manual scoring, statistical significance (P value)	88.6% match with manual scoring, improved accuracy, reduced observer bias.
S44	Manual annotation speed comparison, mean IoU score comparison	Annotation accuracy, mean Intersection over Union (IoU)	AnnotatorJ significantly accelerates hand-annotation tasks and offers high annotation accuracy.
S45	Accuracy, AUROC, cross-validation	Accuracy, AUROC	STRAP outperformed baseline methods in domain generalization tasks.
S46	Cross-validation, independent test cohorts, AUC	AUC, accuracy	CLAM outperforms standard weakly-supervised classification methods, achieves high AUC.
S47	Cross-validation, independent test cohorts, AUC	AUC, accuracy	CRANE outperforms standard weakly-supervised classification methods, achieves high AUC.

S48	Accuracy, precision, recall, F1-score, visual assessment by experts, inter-observer reliability studies	Accuracy, precision, recall, F1-score, visual assessment by experts	Enabled semi-automatic tumor area assessment, object counting, scoring, landmark detection, and morphometric measurements in various imaging studies.
S49	Classifier accuracy, quality of segmentation, user satisfaction	Classifier accuracy, segmentation quality, processing efficiency	ilastik provides efficient and user-friendly workflows for various bioimage analysis tasks.
S50	Performance evaluated by comparison with expert pathologists' annotations, usability tested with non-expert pathologists	Accuracy, F1-score, AUC	High accuracy and F1-score for various types of neoplasms, effective in automatic tumor region detection.
S51	Comparison with manual counting, accuracy in measuring cell features, usability testing with non-programmers	Accuracy, consistency of quantitative measures, ability to handle crowded cell samples, comparison to gold standards (visual inspection, Coulter particle counters)	Accurate in measuring various cell phenotypes, validated against manual counting and other standards.
S52	Not explicitly mentioned	Not explicitly mentioned	Demonstrated utility in assembling and sharing reproducible bioimage analysis workflows.

Supplementary Table 8: Visualization and Interaction Characteristics

ID	Visualization Techniques	Tool/Application Developed	User Interaction	Clinical Application
S1	Heatmaps for highlighting spatial distribution of features, image reconstruction results for cross-stain prediction	Two-stage self-supervised learning framework (CS-CO)	N/A (focuses on algorithm/method development and evaluation rather than user interaction)	Potential for use in computational pathology for tissue classification, cancer prognosis, and subtyping
S2	UMAP projection for patch-level embeddings visualization, Mapper graphs for summarizing ROI and their interactions	WSI-GTFE (GitHub repository: https://github.com/jlevy44/WSI-GTFE)	Not explicitly described, involves interactive analysis via extraction and summarization of high-dimensional data	Determining colon cancer stage, predicting lymph node involvement
S3	Interactive views, waypoints, text annotations, audio narration, interactive UMAP plots for spatial data exploration	Minerva - a narrative image browser for multiplexed tissue images	Interactive navigation using panning, zooming, waypoint selection, channel toggling, and using narratives to guide exploration of complex images	Education, diagnostic support, treatment decision support, research dissemination
S4	UMAP for dimensionality reduction, feature space gating	DEPICTER: A web-based tool for interactive annotation of WSI	Manual gating in feature space, interactive filtering and correction of annotations	Assists pathologists in cancer detection and annotation tasks
S5	Not applicable (no specific visualization techniques were used in this study)	Interactive Web site for Gleason grading using virtual microscopy (http://www.webmicroscope.net/gleason)	Users interact by browsing virtual slides, assigning Gleason scores, and comparing their scores with expert assessments	Improving accuracy and reproducibility of Gleason grading, standardized training, interlaboratory quality control
S6	Visual comparison of stitched sections obtained from different methods provides an implicit visualization	HistoStitcher©	Users interact by selecting pairs of anatomical landmarks using the GUI and specifying constraints on the image transformation	Improved pathologist annotation and multimodal image fusion (e.g., registration of WMH with MRI)
S7	Web-based WSI viewers: Openseadragon, Openlayers, Leaflet, GeoJS, SlideAtlas	Digital Slide Archive (DSA)	Web-based interface for managing image collections, metadata, annotations, and access controls; RESTful programming interface	Collaborative research projects, new image analysis methods development, integration of histopathology with other data types in cancer research
S8	Multiple rendering modes, superimposed neighborhood encodings, cohort view, parallel coordinate plot, UMAP embedding, correlation matrix, scatterplots, bar charts	Visinity: Web-based visual analytics system for spatial neighborhood analysis of multiplexed tissue images	Interactive tools like lasso selection, brushing and linking across views, drag-and-drop interaction for axis reordering, interactive filtering of features	Cancer research and diagnosis, supporting pathologists in identifying and understanding cell interactions and their implications for disease progression and treatment
S9	Overlays on the original image, including classification masks, segmented objects, and manual annotations	Orbit Image Analysis	Graphical user interface for manual annotation, training models, exploring results, and automating tasks through a script editor	Supporting diagnosis and treatment decisions by pathologists, particularly in lung fibrosis, nerve fibre density assessment, and glomeruli detection
S10	Markup images, heatmaps, scatter plots	QuPath	Graphical user interface for loading images, annotating regions of interest, applying analysis algorithms, training classifiers, and visualizing/exporting results	Prognostic biomarker discovery, companion diagnostics, tissue analysis for clinical trials
S11	OpenGL for GPU-based rendering of WSIs and predictions, transparent colored overlays, heatmaps, bounding boxes	FastPathology: Open-source platform for digital pathology	GUI widgets for WSI loading, project management, pipeline creation, result visualization, data analysis, and result export	Improved diagnostic accuracy, research and development in digital pathology, prescreening and prognosis
S12	High-resolution attention heatmaps, SHAP-styled	Segmentation model for ovarian cancer using DIaL and pretrained breast cancer model (GitHub: DMMN-ovary)	Visualization of segmented cancer regions and BRCA mutation prediction, interactive	Screening tool for BRCA mutation prediction, assisting in patient risk

	attribution decision plots, Kaplan-Meier curves		correction of mislabeled regions during training	stratification, supporting diagnosis and treatment decisions
S13	High-resolution attention heatmaps, SHAP-styled attribution decision plots, Kaplan-Meier curves	Pathology-Omics Research Platform for Integrative Survival Estimation (PORPOISE) (pancancer.mahmoodlab.org)	Visualization of raw H&E image pyramidal TIFFs overlayed with attention-based interpretability, local explanations of molecular features, global patterns of morphological and molecular feature importance	Assisting in patient risk stratification, supporting diagnosis and treatment decisions, discovery of novel prognostic biomarkers
S14	Doughnut charts, multi-layer visualizations, clustering visualizations, gene and patient network visualizations, interactive touch and tangible interactions	SynCLay framework for generating synthetic histology images from cellular layouts	Interaction with tangible objects and multi-touch tabletop, selection and filtering of data, construction of queries through tangible interactions (stacking, shaking, tapping)	Research in cancer genomics, understanding genetic mutations and their effects, improving personalized treatment strategies
S15	Retrieval results in list format, images or articles displayed alongside their corresponding metadata	Web-based digital pathology retrieval system integrated with a WSI viewer	Users define ROIs in the viewer and use the retrieval system to search for similar regions, refine searches by adding keywords, selecting specific features, magnification levels	Supporting pathologists in diagnosis and decision-making by providing visually similar cases, facilitating teaching and training by presenting diverse cases for comparison
S16	Heatmaps to highlight spatial distribution of features, scatter plots to visualize relationships between features	SC-Net: Self-supervised framework combining a Segmentation network with a Colorization network	Not explicitly mentioned	Facilitating development of tools for pre-screening, diagnosis, and treatment decision support in pathology
S17	Heatmaps to display tumor probability, uncertainty maps, morphological features extracted from segmented regions	DigiPathAI: Open-source GUI application for histopathology image analysis (GitHub, PyPi)	Not explicitly described, suggested user interaction for uploading, analyzing, and visualizing data	Accurate and efficient initial diagnosis by histopathologists, supporting informed decision-making and treatment planning, providing uncertainty maps to guide further analysis
S18	Heat maps, bi-polar heat maps, color maps, edges, feature maps visualization, and interactive exploration using WebGL	xOpat: Open-source WSI viewer for digital pathology	Interactive exploration, annotation, session sharing, storytelling through a browser-based interface, visualization of neural network feature maps	Remote collaborative diagnosis, pathology education and training, integration of AI algorithms into clinical practice
S19	Segmentation results, not specific visualization techniques mentioned	Deep learning framework (NuClick)	Users provide clicks for nuclei and cells, squiggles for glands, designed to be robust against variations in user input	Accelerating annotation collection in computational pathology, training fully automatic methods for downstream tasks like disease diagnosis and prognosis
S20	Thumbnails with overlay masks, interactive parallel coordinate plots, heatmaps	HistoQC: Open-source software application written in Python	Interactive filtering of data based on metrics and visualized results, clickable thumbnails and rows for higher-magnification views of masks and outputs	Pre-screening tool to identify slides unsuitable for computational analysis, improving repeatability and robustness of digital pathology workflows by identifying and removing artifacts
S21	Qualitative examples of model predictions, highlighting improvements in bounding box detection and misclassification	Novel framework (D E GPR) enhancing existing object detection models	Not explicitly discussed	Celiac disease diagnosis, automated cell counting in pathological settings, further research and clinical validation required before widespread adoption
S22	Multi-resolution pyramid rendering, color space transformations, additive	Slim: Web-based slide microscopy viewer and annotation tool	Interactive panning and zooming, annotation drawing tools, adjustment of	Digital pathology workflows, computational pathology research

	blending of pseudocolor images, overlay of vector and raster graphics for annotations and analysis results		display parameters, selection of presentation states	
S23	Segmentation masks for visualization, not specific visual analytics techniques	Single dynamic neural network (Omni-Seg)	Not explicitly discussed	Automated segmentation of renal tissue, quantitative assessment of tissue types and spatial relationships, aiding in disease diagnosis and prognosis
S24	Web-based whole-slide image viewer utilizing OpenSeadragon for zoomable image navigation	FlexTileSource: New tile source for OpenSeadragon optimized for WSI visualization	Standard WSI viewer interactions (pan, zoom) with faster response times	Improving efficiency of digital pathology workflows by reducing slide loading times
S25	QuPath for interactive visualization of annotations and results, histograms of distances to SVM hyperplane for visualizing classification separations	Informatics system for digital pathology analysis of SBOT vs HGSOC	Interactive annotation of regions of interest, visualization of cell classification and tissue-level results in QuPath	Assisting pathologists in differentiating SBOT and HGSOC
S26	t-SNE to embed extracted representations of ROIs into a 2D space for visualization	Interactive thyroid WSI diagnostic system	Pathologists interact by drawing the region of interest (ROI) on the WSI, system provides classification and retrieval information based on the ROI	Assisting pathologists in diagnosing thyroid nodules, leading to more accurate and efficient diagnosis
S27	Thumbnail images of whole-slide images for quick overview, linking of images to indicate relationships between them, visualization of analysis results (charts, graphs) within cells of the grid	Comparative Pathology Workbench (CPW): Web-based visual analytics platform	Drag-and-drop functionality for arranging images within the grid, menu options for editing cells, adding comments, linking images, access to external resources (OMERO image viewer)	Pathology audit studies, expert review and consensus diagnosis, teaching tool for histology and histopathology
S28	Score-weighted Class Activation Mapping (Score-CAM), Gradient-weighted Class Activation Mapping (Grad-CAM), guided backpropagation	Unsupervised deep learning workflow for histological assessment of kidney biopsy images	Not explicitly described	Standardization of histological assessment, quantitative evaluation of disease progression
S29	Scatter plots, heatmaps, interactive web-based visualization tools	caMicroscope, FeatureScape, and other web-based applications for visual analytics of histopathology images	Interactive web applications for exploring segmentation results, feature values, image patches	Epidemiological studies, clinical decision support, assessment of tumor characteristics and treatment response
S30	Interactive selection of color regions, statistical modeling, ROC curves for performance evaluation	Semi-automatic plugin for ImageJ for color detection in IHC images	Interactive selection of color regions, filtering background pixels, model training and detection phases	Automated quantification of histological biomarkers in IHC images, aiding in the diagnosis of cancers and liver fibrosis
S31	Heatmaps for visualizing prediction confidences, thumbnails for labeled objects	HistomicsML: Open-source web application	Web-based interface for labeling objects by clicking on them in the image, reviewing predictions and adjusting classification rules, exploring images at multiple resolutions	Supporting diagnosis and treatment decisions for lower-grade gliomas, providing prognostic information that complements other clinical and genomic data
S32	2D WSI viewer with zooming/panning, 3D volume reconstruction and visualization with adjustable opacity and clipping planes, overlay of analysis results on images	Digital Pathology Laboratory (DPLab) web platform	Web interface for uploading images, drawing annotations, configuring and running analyses, visualizing and filtering results	Research and clinical settings for tissue-based investigations
S33	Heatmap visualization, clustergrammer for gene expression profiles,	RUN-ONCO: Web-based platform for cancer precision medicine	Web-based interface for data management and analysis, interactive visualization	Enhancing cancer research and treatment, supporting precision medicine,

	Cytoscape.js for protein-protein interaction networks		plugins, REST API for flexible access to data	facilitating personalized diagnosis and treatment plans
S34	Channel-based and cell-based rendering, histograms, radial charts, segmentation with color-coding, sliding-window search algorithm for similar region identification	Scope2Screen: Web-based application with a Python backend and a JavaScript frontend	Interactive exploration and analysis of multi-channel images, switching between different image channels, applying filters and color mappings, close-up inspections using the lensing feature, annotation and summarization of regions of interest, saving snapshots, and exporting data	Patient-facing (translational) cancer research, diagnosis and patient care
S35	Overlay of annotations on the original WSI with transparency, virtual magnification indicator for visual approximation of the microscope's zooming power	OpenHI: Open-source platform for collaborative histopathological image annotation	Web-based GUI providing a WSI viewer, virtual magnification indicator, control panel for annotation configuration, slide information panel	Creating large-scale datasets with precise and semantically rich annotations, training computational models for automated diagnosis, mass screening, and phenotype-genotype association studies
S36	Original images view, feature extraction view, line matching view, warp image view, chessboard view, annotation view	MMIR: Web application for multimodal histological image registration	Interactive project management, algorithm management, visualization of registration results	Multimodal histological image analysis and research
S37	Attention maps, heatmaps, retrieval result visualizations	Framework for cross-modal retrieval of WSIs and diagnosis reports	Interactive retrieval using image or text inputs to find relevant cases from the database	Aids in diagnostic reference by retrieving similar cases, supports pathologists in diagnosis and treatment planning
S38	Attention maps, clustering of high-score patches using Gaussian mixture models (GMM), t-SNE for dimensional reduction	PathoRiCH: Histopathologic image-based deep learning classifier	Not explicitly described beyond visualization of model decisions	Guiding patient-tailored therapy in HGSOC, aiding in selection of primary and maintenance treatments, planning surveillance frequency, counseling patients about clinical trials
S39	Heatmaps for predictions, uncertainty and attention, UMAP plots for feature space visualization, mosaic maps, saliency maps (Grad-CAM, vanilla gradients, integrated gradients, XRAI), synthetic histology using conditional GANs	Slideflow and Slideflow Studio: Whole-slide user interface for generating predictions, heatmaps, feature space visualizations	Interactive filtering of features, brushing and linking across different visualizations, real-time predictions and heatmap generation, interactive zoom and inspection of mosaic maps	Diagnostic support, risk stratification, treatment selection based on digital histopathology
S40	Color-coded annotation layers overlaid on WSIs, interactive UI for toggling visualization layers	ComPRePS: Open-source cloud-based computational tool for data upload, storage, digital histology visualization, and complex image analysis tasks	Interactive UI for panning, zooming, displaying annotation layers on WSIs, editing FTU mask boundaries	Supporting diagnosis and treatment decisions by pathologists, clinical diagnostics and research
S41	Heatmaps, scatter plots for feature relationships, interactive annotation layers in HistomicsUI	Histo-Cloud: Cloud-based tool for segmentation of whole slide images	Interactive filtering, brushing and linking across visualizations, web-based interface for uploading and analyzing WSIs	Supporting diagnosis and treatment decisions by pathologists, research in renal disease pathologies
S42	None specified explicitly	Code and pretrained models available at: GitHub	None specified explicitly	Enhancing histopathology image analysis with limited manual annotations
S43	Histogram profiles	IHC Profiler plugin for ImageJ	Interactive analysis via ImageJ, choice of color deconvolution, threshold setting	Screening of prognostic biomarkers, unsupervised analysis in clinical and research laboratories
S44	Contour assist mode, U-Net presegmentation	AnnotatorJ ImageJ plugin	Manual refinement of presegmented contours, interactive annotation assistance	Not specifically discussed for clinical applications

S45	Saliency maps, integrated gradients	STRAP (Style TRansfer Augmentation for histoPathology)	Not explicitly discussed in detail	Supports diagnosis in pathology by improving classification robustness
S46	Attention heatmaps, PCA for feature space visualization	CLAM (Clustering-constrained Attention Multiple instance learning)	Interactive demo available online for heatmap visualization	Diagnostic support in computational pathology, telepathology
S47	Attention heatmaps, PCA for feature space visualization	CRANE (Cardiac Rejection Assessment Neural Estimator)	Interactive demo available online for heatmap visualization	Diagnostic support in computational pathology, telepathology
S48	Zoomable, tile-based viewer, annotation layers, ontology editor, annotation galleries, textual search engine, proofreading tools	Cytomine platform for collaborative analysis and visualization of multi-gigapixel imaging data	Interactive filtering of features, brushing and linking across different visualizations, manual and semi-automatic annotation tools	Pathology for tumor segmentation, cell counting, morphometric measurements
S49	Interactive segmentation, probability maps, boundary maps	ilastik software for interactive bioimage analysis	Interactive training, live prediction updates, batch processing	Segmentation and analysis of tissue images for pathology research
S50	Heatmaps, interactive annotation tools, region labeling	HistoColAi platform for annotation and visualization of histopathology images with embedded AI models	Users can annotate, label, navigate WSIs, collaborate with other pathologists through the platform's web interface	Diagnostic support tool for pathologists, improving efficiency and accuracy in tumor detection
S51	None explicitly mentioned related to histopathology	CellProfiler: Open-source software for cell image analysis	Graphical user interface for setting up analysis pipelines, point-and-click interface for non-programmers, ability to save and share analysis pipelines	Not applicable
S52	Integrated visual programming framework, interactive user interface, various visualization tools for image analysis workflows	ICY platform for bioimage informatics and extended reproducible research	Users can develop, share, extend image analysis protocols through a visual programming interface and a centralized online repository	Not applicable

Supplementary Table 9: Reproducibility and Reporting

ID	Open-Source	Development Level	Solution Name	Diseases
S1	GitHub	Framework	CS-CO	Colorectal Cancer
S2	GitHub	Tool	WSI-GTFE	Colon Cancer
S3	GitHub	Tool	Minerva Story	Multiple Diseases (Cancer)
S4	GitHub	Tool	Depicter	Breast Cancer
S5	Web	Tool	Webmicroscope	Prostate Cancer
S6	GitHub	Tool	HistoStitcher	Prostate Cancer
S7	GitHub	Software	Digital Slide Archive	Multiple Diseases (Cancer)
S8	GitHub	Tool	Visinity	Lung Cancer
S9	GitHub	Software	Orbit Image Analysis	Idiopathic Pulmonary Fibrosis
S10	GitHub	Software	QuPath	Colon Cancer
S11	GitHub	Software	FAST Pathology	Breast Cancer
S12	GitHub	Tool	DMMN-ovary	Ovarian Cancer
S13	GitHub	Tool	PanCancer	Multiple Diseases (Cancer)
S14	GitHub	Framework	SynCLay	Multiple Diseases (Cancer)
S15	C4Science	Tool	Desuto Platform	Multiple Diseases (Cancer)
S16	GitHub	Framework	SC-Net	Multiple Diseases (Cancer)
S17	GitHub	Tool	DigiPathAI	Colon Cancer
S18	GitHub	Tool	Xopat	Multiple Diseases (Cancer)
S19	GitHub	Framework	NuClick	Multiple Diseases (Cancer)
S20	GitHub	Software	HistoQC	Breast Cancer
S21	GitHub	Framework	DeGPRR	Colon Cancer
S22	GitHub	Tool	SLIM	Multiple Diseases (Cancer)
S23	GitHub	Tool	Omni-Seg	Kidney Disease
S24	GitHub	Tool	FlexTileSource	Multiple Diseases (Cancer)
S25	GitHub	Tool	Cellular Composition	Ovarian Cancer
S26	GitHub	Tool	Thyroid Interactive	Thyroid Cancer
S27	GitHub	Tool	Comparative Pathology	Multiple Diseases (Cancer)
S28	GitHub	Framework	Glomerulus Clustering	Kidney Disease
S29	GitHub	Tool	Quip Distro	Multiple Diseases (Cancer)
S30	NIH	Tool	IHC Toolbox	Colon Cancer
S31	GitHub	Software	HistomicsML	Lower Grade Glioma (LGG)
S32	GitHub	Framework	Digital Pathology	Multiple Diseases (Cancer)
S33	GitLab	Tool	Run Onco	Multiple Diseases (Cancer)
S34	GitHub	Tool	Scope2Screen	Multiple Diseases (Cancer)
S35	GitLab	Software	OpenHI	Multiple Diseases (Cancer)
S36	GitHub	Tool	MMIR	Multiple Diseases (Cancer)

S37	GitHub	Framework	FGCR	Gastric Cancer
S38	GitHub	Tool	PathoRICH	Ovarian Cancer
S39	GitHub	Software	Slideflow	Multiple Diseases (Cancer)
S40	GitHub	Tool	ComPRePS	Kidney Disease
S41	GitHub	Tool	Histo Cloud	Kidney Disease
S42	GitHub	Framework	SSL CR Histo	Breast Cancer
S43	GitHub	Tool	IHC Profiler	Multiple Diseases (Cancer)
S44	GitHub	Tool	AnnotatorJ	Multiple Diseases (Cancer)
S45	GitHub	Framework	Style Transfer for Digital Pathology	Breast Cancer
S46	GitHub	Tool	CLAM	Breast Cancer
S47	GitHub	Tool	CRANE	Multiple Diseases (Cancer)
S48	GitHub	Software	Cytomine	Multiple Diseases (Cancer)
S49	GitHub	Software	Ilastik	Multiple Diseases (Cancer)
S50	GitHub	Software	HistoColAi	Multiple Diseases (Cancer)
S51	GitHub	Software	CellProfiler	Multiple Diseases (Cancer)
S52	ICY	Software	ICY	Multiple Diseases (Cancer)

Supplementary Table 10: Strengths and Limitations

ID	Strengths	Limitations
S1	Utilizes domain-specific knowledge without requiring side information, achieves high accuracy and robustness, effective on multiple histopathology tasks	Relies on computationally intensive stain separation and vector perturbation; limited to H&E stains; tested on a limited set of datasets; not extensively validated via user studies
S2	High classification accuracy, interpretable summaries of tissue interactions, enhances understanding of tissue architecture in disease progression	Coarse physician annotations may limit accuracy, limited exploration of Mapper parameter space, potential for clustering effects due to repeated measurements
S3	User-friendly, interoperable with various datasets, supports web-based interactions, enhances learning and data dissemination	Depends on the initial data quality and completeness of annotations, requires effort from experts to create detailed guides
S4	User-friendly, interactive, accurate segmentation, minimal training required	Requires high computational resources, performance variability with different networks
S5	Demonstrates that Web-based virtual microscopy is a viable tool for teaching and standardizing Gleason grading. Provides a platform for self-testing and learning. Achieves good intraobserver agreement for Gleason grading.	Study limited to a small sample size (62 biopsies). No effort was made to standardize the pathologists' Gleason grading beforehand. The interobserver agreement among the 3 expert uropathologists was only moderate to substantial.
S6	HistoStitcher© is fast, memory efficient, and produces high-quality stitches, even for high-resolution images.	The paper does not discuss specific limitations of HistoStitcher©. However, it is implied that the program might require substantial computational resources for high-resolution images.
S7	Open-source and freely available, reducing cost and allowing customization; Scalable for large image datasets; Integrates with existing image analysis libraries like HistomicsTK; Provides a user-friendly web interface	Initially designed for deidentified data and may not have the security features required for HIPAA compliance; Limited quantitative evaluation of image analysis algorithms
S8	Visinity's key strengths include its scalability to large datasets, intuitive user interface, ability to analyze and compare patterns across multiple specimens, and integration of visual querying and exploration functionalities.	Dependence on single-cell information, challenges in analyzing 3D imaging data, and need for further user studies for usability evaluation
S9	Orbit offers a comprehensive, flexible, and scalable framework for WSI analysis, integrating machine learning and tile-processing techniques. It provides a user-friendly interface, script editor, and the ability to integrate external tools.	Performance may be limited by the quality of the training data and the complexity of the histopathology images. It might require significant computational resources for complex deep learning tasks.
S10	Comprehensive functionality: Provides a wide range of tools for analyzing and exploring whole slide images; User-friendly interface; Open source: Allows for collaboration and extension by the research community; High performance: Capable of processing large datasets efficiently.	Limited to specific histopathology types: The study primarily focused on colon cancer. Further testing is needed on other histopathology types. Requires high computational resources: Analyzing whole slide images can be computationally intensive. Limited user studies for usability evaluation.
S11	Open-source: Provides access to source code for community contributions and customization; User-friendly interface: Enables users without programming experience to deploy and utilize deep learning models; Efficient memory management: Minimizes memory consumption for handling large WSIs; High performance: Achieves competitive runtime performance with GPU inference; Flexible model deployment: Supports multiple inference engines and model architectures; Real-time visualization: Provides interactive visualization of predictions as overlays.	Limited user studies: The platform's usability was not extensively evaluated with end-users; Hardware dependency: Performance can be affected by hardware capabilities, particularly GPU availability and speed; Model complexity: Runtime can be influenced by model complexity, requiring adjustments in batch size and other parameters; Limited annotation features: The platform does not currently include advanced annotation tools.
S12	Reduces manual annotation time significantly using DIaL with pretrained model, accurate segmentation of ovarian cancer, potential for mutation-related pattern discovery, user-friendly interface	Suboptimal performance in predicting BRCA mutation, potential false positives/negatives due to underrepresented training data, further validation required for broader application
S13	Achieves high classification accuracy, improves interpretability compared to black-box models, user-friendly interface, enables discovery of new prognostic biomarkers, supports exploration and validation of multimodal biomarkers	Limited to specific cancer types, requires high computational resources, interpretability does not always explain why discovered features are prognostic, needs further validation with larger datasets
S14	Effective for collaborative data exploration, intuitive interface, supports complex data manipulation and visualization, promotes understanding and discussion	Requires cellular layouts for high-quality image generation, some minor imperfections in generated nuclei masks, reliance on TheCoT for generating layouts from user-defined parameters
S15	Combines visual and text-based retrieval, integrates with a dynamic WSI viewer, uses deep learning models for improved feature extraction and retrieval performance	Requires manual annotation for training the deep learning model, limited to specific histopathology types, requires substantial computational resources
S16	The method achieves high segmentation accuracy while significantly reducing annotation efforts. It is robust to perturbations in point annotations.	Requires high computational resources. The method relies on complete point annotations. Further user studies for usability evaluation are needed.

S17	Generalizable framework across multiple cancer sites, ensemble segmentation for improved performance, efficient patch-based processing, uncertainty estimation for aiding interpretation	Limited discussion on user interaction and usability, requires specialized knowledge of deep learning and histopathology, potential for bias due to limited data heterogeneity
S18	Flexibility, modularity, supports various data inputs, enhances collaboration, provides explainability for AI decisions, and efficient real-time visualization.	Requires further development for hierarchical annotation categories and user progress tracking.
S19	NuClick offers minimal user interaction, high accuracy, robustness to variations in user input, and generalizability across datasets and domains.	The study does not mention specific user studies for usability evaluation.
S20	Open-source and freely available; User-friendly interface; Offers a comprehensive set of artifact detection and batch effect analysis tools; Achieves high agreement with human pathologists; Provides quantitative metrics for evaluating slide quality.	Limited to specific histopathology types (H&E bright-field); Might require significant computational resources; Limited user studies for usability evaluation; Does not currently detect compression artifacts.
S21	Improves the performance of existing object detectors, model-agnostic (can be applied to various object detection models), leverages both expert knowledge and data-driven features for more accurate cell detection and counting, shows promising results in downstream tasks like celiac disease classification.	Relies on the availability of expert knowledge to define explicit features, requires significant computational resources for training and inference, lacks a comprehensive evaluation of user experience and usability.
S22	Interoperability with DICOM-compliant systems, support for both brightfield and fluorescence microscopy, client-side processing for improved performance	Requires conversion of non-DICOM formats, dependency on DICOMweb services
S23	Single dynamic network architecture for multi-label and multi-scale segmentation; Explicit modeling of scale information; Semi-supervised learning with pseudo-labels improves accuracy; Superior performance compared to existing methods; Generalizability to different species (human and mouse) without retraining.	Computational time for aggregating tissue-wise segmentation results into a final multi-label mask; Not designed for visual analytics or user interaction; Limited to specific histopathology types (renal pathology).
S24	Significantly faster FOV rendering, especially for WSI with more native levels. Compatible with arbitrary pyramid structures.	Performance gain may vary depending on WSI native level structure
S25	Achieves high classification accuracy, interpretable features, interactive annotation process	Limited to specific ovarian tumor types, requires manual annotation for training, does not address all histologic features used in clinical practice (e.g., stromal invasion)
S26	The study demonstrates that an interactive system based on ROI selection can be more efficient and effective compared to analyzing the entire WSI directly. The deep learning based representation generation and fusion outperform traditional methods like GIST. The system provides valuable assistance to pathologists, allowing them to make more informed decisions.	The study focuses specifically on thyroid frozen sections. The number of "Uncertain" samples used for training might be limited, affecting the performance on this category. The study doesn't explore usability evaluation through user studies.
S27	Facilitates collaborative analysis and discussion of histopathology images; Provides an interactive and user-friendly interface for comparing images; Integrates with various external resources for image analysis and data visualization; Open-source and available for public use.	Requires internet connectivity; Can be slow to render for large workbenches with many images; Not suitable for constrained devices with small screens.
S28	Unsupervised approach avoiding manual labeling, quantitative assessment of histological findings, visualization of decision rationale	Short observation period limiting prognostic evaluation, subjective evaluation of clustering results, low R^2 values in linear models
S29	High classification accuracy, modular design, robust feature extraction, scalability, and user-friendly interface	High computational requirements, limited to specific cancer types, need for extensive validation studies
S30	High classification accuracy, robust across different datasets and users, user-friendly interface	Limited to specific color stains, requires careful selection of training pixels, potential inter-observer variations
S31	HistomicsML enables efficient training of classifiers with minimal user input, improves prognostication for lower-grade gliomas, facilitates investigation of genotype-phenotype associations	Requires existing segmentation and feature extraction algorithms, scalability is limited by memory footprint of feature data, inter-reader variation is not systematically addressed
S32	Cloud-based, accessible via web browsers, supports 3D reconstruction, extensible algorithm library	Full functionality only supported in Google Chrome browser
S33	High extensibility with plugins, integration of clinical and -omics data, flexible data management, efficient data analysis and visualization	Requires further development for more complex analyses, potential challenges in handling extremely large datasets
S34	Provides a seamless visual experience, supporting interactive exploration and analysis of multi-channel images. Offers intuitive interaction with large multi-channel images and linked multivariate data. Enables pathologists and biologists to characterize tumor features and perform spatial analysis.	Does not support analysis and annotation of image collections or work interactively with image analysis pipelines. Lacks certain desired features, such as simultaneous access to single-cell marker intensity distribution and improved handling of occlusion by the lens.
S35	Achieves high annotation precision with pixel-level accuracy; Supports online collaboration between multiple users; Offers multiple pre-segmentation levels	Does not include methods for consolidating multi-expert annotations; Does not address inter-rater reliability; Pre-segmentation method could be improved by incorporating nuclei localization; Relies on the WSI pixel size parameter, which is not always provided in WSI metadata; Processing

	for user-adjustable precision; Provides a virtual magnification indicator for accurate zooming approximation; Is open-source and freely available.	time increases with larger areas of the image being viewed; Limited user studies for usability evaluation.
S36	User-friendly interface, supports multiple registration algorithms, adaptable architecture, enables annotation transfer across modalities	Specific algorithm performance may vary, complexity in handling diverse image types
S37	Handles large-scale WSIs, leverages diagnostic reports for training without additional annotations, enables fine-grained semantic retrieval	Dependence on accurate diagnostic reports, limited to specific types of histopathology
S38	High predictive performance, integration with molecular biomarkers for enhanced stratification, identification of key histologic features associated with treatment response	Suboptimal performance in external validation cohorts, need for additional multicenter validations, limited performance in predicting BRCA mutation and HRD status
S39	Comprehensive, flexible, supports multiple deep learning backends, optimized for computational efficiency, includes real-time visualization and interpretability features.	Some functions require specific deep learning backends, limited support for graph neural networks (planned for future updates).
S40	High classification accuracy, user-friendly interface, open-source software, scalable, facilitates collaboration and democratization of AI in digital pathology.	Limited to specific histopathology types, requires high computational resources, limited user studies for usability evaluation.
S41	High classification accuracy, user-friendly interface, adaptable for different tissue types and stains.	Requires high computational resources, limited to specific histopathology types, limited user studies for usability evaluation.
S42	Effectively leverages unlabeled data, robust to noisy input data	Limited to specific tasks, dependence on data augmentation
S43	Open source, reduces observer bias, compatible with ImageJ, high match percentage with manual scoring	May require expert supervision, not compatible with membrane immunomarkers
S44	Accelerates annotation tasks, integrates deep learning for improved pre-segmentation	Requires manual refinement of contours, limited to users familiar with ImageJ
S45	Improves generalizability, addresses domain shifts effectively	Computationally expensive, requires large dataset for training
S46	Data-efficient, interpretable, adaptable to different data sources	Requires large datasets for training, computationally expensive
S47	Data-efficient, interpretable, adaptable to different data sources	Requires large datasets for training, computationally expensive
S48	Facilitates collaborative analysis, supports large-scale image data, extensible architecture, open-source availability.	Recognition performance depends on image quality, acquisition protocols, and quantity of annotations.
S49	User-friendly, efficient processing of large datasets, interactive machine learning	Requires high-quality boundary maps, limited to predefined workflows
S50	Open-source, user-friendly interface, supports collaborative annotation, integrates AI for predictive analysis	Limited to specific histopathology types, requires high computational resources, initial setup may be complex for inexperienced users
S51	Open-source, flexible, supports high-throughput analysis, accurate cell identification and measurement, user-friendly interface, compatible with various cell types and assays.	Limited support for time-lapse and 3D image stack analysis, initial setup may take several days for new users, computationally intensive tasks require powerful hardware or cluster computing.
S52	Open-source, user-friendly interface, supports community contribution, facilitates reproducible research.	Limited to the scope of bioimage informatics, requires initial learning for effective use, dependent on community contributions for extensibility.

Supplementary Table 11: Open-Source Histopathology Image Analysis and Their User Manuals

ID	Solution name	Links	User manual
S1	CS-CO	GitHub	"Clone and Install: Clone the repository using git clone https://github.com/easonyang1996/CS-CO.git and install dependencies with pip install -r requirements.txt.<br>Prepare Data: Organize your dataset as specified in the repository, ensuring proper folder structure for training and testing.<br>Train and Evaluate: Train the model using CUDA_VISIBLE_DEVICES=0 python3 -u 4_csco_train.py configs/NCT_CRC/cs_conf_resnet18.ini and evaluate with the provided scripts."
S2	WSI-GTFE	GitHub	Installation: Clone the repository with git clone https://github.com/jlevy44/WSI-GTFE.git and navigate to the directory. Use the included example notebooks to get started.<br>Data Preparation: Follow the example in the notebooks directory, such as 1_create_graph_dataset.ipynb, to create a graph dataset from your slide images.<br>Usage: The code currently supports inductive patch-level classification. Customize the example notebooks for your dataset to perform analysis.
S3	Minerva Story	GitHub	Clone the Repository: Clone the repository using git clone git@github.com:labsyspharm/minerva-story.git and navigate to the directory.<br>Set Up Environment: Install Node.js and npx for running a local server. Install npx globally using npm install -g npx.<br>Run Local Server: Start the local server with npx http-server -p 8000 and access it at localhost:8000 in your browser.
S4	Depicter	GitHub	Installation: Create a new conda environment with conda env create -n depicter_env -f environment.yml and activate it using conda activate depicter_env. This will set up the necessary dependencies.<br>Setting Up TissUUmaps: Install TissUUmaps as described in its documentation. Then, start TissUUmaps, navigate to the Plugins menu, add the DEPICTER plugin, and restart TissUUmaps to enable the plugin.<br>Using Depicter: Load your images and masks, extract patches using extract_patches.py, and generate embeddings with generate_embeddings.py. You can then use TissUUmaps with the DEPICTER plugin for interactive annotation.
S5	Webmicroscope	Web	Contact Author
S6	HistoStitcher	GitHub	Download and install "Histostitcher_Setup" on a Windows 7 (64-bit) system with OpenGL 3.0. The installation includes the necessary Matlab runtime and AMD SDK.
S7	Digital Slide Archive	GitHub	Docker Installation:<br>1. Install Prerequisites:<br>- Docker<br>- Docker Compose<br>2. Clone the Repository:<br>git clone https://github.com/DigitalSlideArchive/digital_slide_archive<br>3. Navigate to the Directory:<br>cd digital_slide_archive/devops/dsa/<br>4. Pull Docker Images:<br>docker compose pull<br>5. Start the Digital Slide Archive:<br>DSA_USER=$(id -u):$(id -g) docker compose up<br>6. Access the Girder instance:<br>http://localhost:8080<br>7. Stop the Digital Slide Archive:<br>docker compose down -v<br>Additional Steps:<br>- Add Sample Data:<br>python utils/cli_test.py dsarchive/histomicstk:latest --test<br>- Development:<br>- Access containers: docker compose exec girder bash<br>- Restart Girder: restart_girder.sh<br>- Rebuild and restart Girder: rebuild_and_restart_girder.sh<br>Usage:<br>- Web interface accessible at http://localhost:8080<br>- Log in with admin credentials<br>- Use Slicer CLI Web plugin settings to add default tasks
S8	Visinity	GitHub	Installation:<br>Conda Environment: Install Conda and create an environment using the command conda env create -f requirements.yml. Activate the environment with conda activate visinity.<br>Start Server: Run python run.py to start the web server. You can then access the tool at http://localhost:8000/.<br>Setting Up:<br>Node.js Setup: If you plan to edit JavaScript code, install Node.js, navigate to /minerva_analysis/client, and run npm install to install dependencies. Use npm run start to package the JavaScript, or npm run watch to edit dependencies.<br>Usage:<br>Data Import: Import images, segmentation masks, and single-cell quantifications via the GUI. Match CSV columns with channels in the image and use the various views (e.g., UMAP embedding, neighborhood composition) for analysis.
S9	Orbit Image Analysis	GitHub	Clone the repository using git clone https://github.com/mstritt/orbit-image-analysis.git.<br>Build the project using Gradle with the command gradle jar.<br>Run the application with the command: java -Djavax.xml.parsers.DocumentBuilderFactory=com.sun.org.apache.xerces.internal.jaxp.DocumentBuilderFactoryImpl -cp "build/libs/orbit-image-analysis.jar;build/libs/lib/*" com.actelion.research.orbit.imageAnalysis.components.OrbitImageAnalysis<br>Setting Up:<br>Data Preparation: Store training data in the data directory with images in JPEG format and masks in PNG format. Use preprocess_data.py to prepare data by resizing images to 512x512 and creating vector masks.<br>Training: Generate dataset files using generate_dataset_txt.py and start training with main.py. Adjust hyperparameters in main.py as needed.Usage:<br>TensorBoard: Visualize training progress using TensorBoard by running tensorboard --logdir=log.<br>Testing/Validation: Adjust parameters in main.py and run with the test option to evaluate the model's performance.

S10	QuPath	GitHub	"Download: Choose the appropriate installer for your operating system from the QuPath download page. Options are available for Windows, macOS (both Intel and Apple Silicon), and Linux.<br>After installation, you can explore the software's features through the step-by-step guides and video tutorials."
S11	FAST Pathology	GitHub	"Windows:<br>Download the installer from the release page.<br>Run the installer and follow the prompts. If a security warning appears, select ""More info"" and then ""Run anyway"".<br>Launch the software from the Start menu.<br>Ubuntu Linux:<br>Ensure OpenCL is installed (CUDA for NVIDIA, OpenCL NEO for Intel, ROCm for AMD).<br>Download the Debian package from the release page and install it using sudo dpkg -i fastpathology_ubuntu*.deb.<br>Run the software from the /opt/fastpathology/bin/ directory or search for it in the Ubuntu menu.<br>macOS:<br>Install Homebrew, then install OpenSlide and libomp using brew install openslide libomp.<br>Download and open the appropriate .dmg file for your CPU architecture (x86_64 for Intel, arm64 for Apple Silicon) from the release page.<br>Drag the FastPathology app to the Applications folder and open it.<br>Additional Features:<br>NVIDIA GPU Inference: For accelerated performance, install CUDA, cuDNN, and TensorRT for NVIDIA GPUs. Set the environment variable CUDA_VISIBLE_DEVICES to select the desired GPU."
S12	DMMN-ovary	GitHub	"Setup:<br>Install Anaconda with Python 3.7.7 and OpenSlide tools (sudo apt-get install openslide-tools).<br>Create and activate the conda environment with clam.yaml:<br>conda env create -n clam -f docs/clam.yaml<br>conda activate clam<br>Install additional packages like smooth-topk.<br>Data Preparation:<br>Download WSIs and molecular data. Use the CLAM toolbox to extract features and store them as .pt files.<br>Execute experiments with:<br>CUDA_VISIBLE_DEVICES=<DEVICE ID> python main.py --which_splits <SPLIT FOLDER PATH> --split_dir <CANCER TYPE> --mode <MODALITY> --model_type <MODEL>"
S13	PanCancer	GitHub	"Set Up Environment:<br>Install Anaconda with Python 3.7.7.<br>Install OpenSlide tools using sudo apt-get install openslide-tools.<br>Create and activate a new conda environment using the provided clam.yaml file:<br>conda env create -n clam -f docs/clam.yaml<br>conda activate clam<br>Install the necessary package for experiments, smooth-topk:<br>git clone https://github.com/oval-group/smooth-topk.git<br>cd smooth-topk<br>python setup.py install<br>Data Preparation:<br>Download diagnostic WSIs and molecular profile data from the NIH Genomic Data Commons Data Portal and cBioPortal.<br>Process the data using the CLAM WSI-analysis toolbox to extract 256x256 patches and save them as .pt files."
S14	SynCLay	GitHub	"Setup:<br>Environment: Install Python 3.6 and PyTorch 1.10. Clone the repository and navigate to the project directory.<br>Dependencies: Install necessary packages as specified in the repository's documentation.<br>Data Preparation:<br>Use the scripts in the preprocessing directory to collect semantic masks for datasets like CoNiC and PanNuke.<br>Training:<br>Adjust the paths and values in main.py and train the model with the command:<br>python main.py<br>Inference:<br>For generating images, use:<br>python main.py --mode test<br>For generating images from cellular layouts, use:<br>python main.py --mode thecot"
S15	Desuto Platform	C4Science	To install and use the Desuto Platform, follow these steps:<br>Prerequisites: Ensure Docker CE and Docker Compose are installed. A machine with at least 10GB RAM is recommended.<br>Setup: Define the necessary data paths in the .env file and configure volumes for uploaded images and retrieval data.<br>Start Containers: Use docker-compose up -d to start all containers and docker-compose stop to stop them. Access the Web Viewer at http://localhost after a few minutes.
S16	SC-Net	GitHub	"Install: Clone the repo and install dependencies:<br>git clone https://github.com/hust-linyi/SC-Net.git<br>pip install -r requirements.txt<br>Data Preparation: Download and preprocess datasets using provided scripts.<br>Train: Configure parameters in options.py and start training: python train.py<br>Run the model with: python test.py"
S17	DigiPathAI	GitHub	"Installation:<br>Just the UI: pip install DigiPathAI<br>Full AI Pipeline: pip install ""DigiPathAI[gpu]""<br>This installs the UI along with dependencies for the AI segmentation pipeline, including PyTorch, TensorFlow, and OpenCV.<br>Usage:<br>Local Server: Navigate to the directory containing the OpenSlide images and start the server with: digipathai <host: localhost (default)> <port: 8080 (default)><br>Python API: For segmentation, use:"
S18	Xopat	GitHub	Setup:

			Install Node.js and npm. Run the following commands to set up the environment:<br>npm install<br>grunt env<br>Deployment:<br>You can run the viewer locally or with Docker. For a local setup, use: npm run node<br>For a Docker setup, clone the xOpat Docker repository and use:<br>docker compose build<br>docker compose up<br>Configuration:<br>Configure the viewer with the desired settings in env/env.json. You can also add data to the ./data/ folder for viewing.<br>Access:<br>The default viewer address is http://localhost:8080. Ensure proper permissions are set for the data directory.
S19	NuClick	GitHub	Setup:<br>Clone the repository and install the required dependencies using: pip install -r requirements.txt<br>The required packages include Keras, TensorFlow, OpenCV, and other necessary libraries.<br>Inference:<br>Download the pre-trained weights for nucleus segmentation from the provided link and save them inside the weights folder.<br>Set the application in config.py as per your use case.<br>To run the interface, use: python test.py<br>For processing existing detection points, save the points in .mat format and update the mat_path, images_path, and save_path in config.py. Then run: python test_all_images.py
S20	HistoQC	GitHub	Docker Installation<br>Pull the Docker Image: docker pull histotools/histoqc:master<br>Run the Docker Container: docker run -v <local-path>:/data --name <container-name> -p <local-port>:5000 -it histotools/histoqc:master /bin/bash<br>This command mounts your local data directory, starts a container, and exposes the web interface on your host machine.<br>Start the Container:<br>If you stop the container, restart it with: docker start -i <container-name><br>Pip Installation<br>Clone the Repository: git clone https://github.com/choosehappy/HistoQC.git<br>cd HistoQC<br>Install Requirements:<br>pip install -r requirements.txt<br>pip install .<br>Usage<br>Running HistoQC:<br>python -m histoqc -c config.ini -n 3 "*.svs"<br>Replace "*.svs" with your file pattern and config.ini with your configuration file.<br>Viewing Results:<br>Launch the UI server to view results in your browser: python -m histoqc.ui ./histoqc_output/results.tsv<br>Navigate to http://<hostname>:5000 to access the interface.
S21	DeGPRR	GitHub	Setup:<br>Clone the repository and navigate to the directory:<br>git clone https://github.com/dair-iitd/DeGPR.git<br>cd DeGPR<br>Install the required dependencies: pip install -r requirements.txt<br>Data Preparation:<br>Prepare your data as per the guidelines provided in the repository. The datasets used include CoNSeP, MoNuSAC, and MuCeD. For accessing the MuCeD dataset, fill out the form provided by the repository.<br>Model Weights:<br>Download pre-trained model weights from the provided link and place them in the appropriate directory.<br>Running the Model:<br>Use the provided scripts for training and evaluation. The detailed commands and configurations are outlined in the README file.
S22	SLIM	GitHub	Setup:<br>Clone the repository and navigate to the directory:<br>git clone https://github.com/ImagingDataCommons/slim.git<br>cd slim<br>Install the necessary dependencies and build the application:<br>yarn install<br>PUBLIC_URL=/ yarn build<br>Local Deployment:<br>Use Docker to set up a local instance with the provided Docker Compose file: docker-compose up -d<br>The app will be available at http://localhost:8008, and the DICOMweb services will be accessible at http://localhost:8008/dcm4chee-arc/aets/DCM4CHEE/rs.
S23	Omni-Seg	GitHub	Docker Setup:<br>Pull the Docker image: sudo docker pull lengh2/omni_seg<br>Run the container, specifying the input and output directories:<br>export input_dir=/home/input_dir<br>sudo mkdir $input_dir<br>export output_dir=$input_dir/output<br>sudo nvidia-docker run --shm-size 64G -it --rm -v $input_dir:/INPUTS/ -v $output_dir:/OUTPUTS lengh2/omni_seg<br>Data Preparation:<br>For single image processing, create folders for different magnifications (40X, 10X, 5X) and place corresponding PNG files in them. Ensure the directories "clinical_patches," "segmentation_merge," and "final_merge" are empty before starting the process.

			Navigate to the Omni_seg_pipeline_gpu directory and execute the scripts in the specified order.<br>The final output will be saved in the "final_merge" folder.
S24	FlexTileSource	GitHub	Installation:<br>npm: Install via npm with the command: npm install openseadragon<br>CDN: Alternatively, you can use a CDN like jsDelivr or CDNJS for quick integration into your web projects.<br>Setup:<br>Include the OpenSeadragon library in your project and initialize the viewer with your desired options. For example:<br>var viewer = OpenSeadragon({<br>id: "openseadragon1",<br>prefixUrl: "/path/to/images/",<br>tileSources: "/path/to/your/image.dzi"});
S25	Cellular Composition	GitHub	"patch classification<br>Draw ROIs (10 ROIs each case), and then process ROIs<br>detect cells in ROIs, export measurements (preparePatchClassification.groovy)<br>use the trained cell classifier to classify cells (../CellClassification/hand_crafted/cell_prediction.py)<br>import classification result (cell labels) into QuPath (load_updated_measurements.groovy)<br>export patches and masks<br>with cell classification results, prepare data for training and testing (aggregate cellular features as patch descriptors ...)<br>train patch classification model<br>Validation i. ROC curve ii. Confusion matrix iii. Feature importance vi. Visualize misclassified patches (import into QuPath: load_patch_locations.groovy)"
S26	Thyroid Interactive	GitHub	"Setup:<br>Clone the repository and install the required dependencies using: pip install -r requirements.txt<br>Data Preparation:<br>The system uses preselected suspicious regions identified by pathologists. It involves generating feature representations for these regions by extracting and fusing patch features using deep neural networks.<br>Modules:<br>patchCLS: Classifies image patches to extract features.<br>genFeas: Generates feature representations for analysis.<br>roiCLS: Diagnoses suspicious regions.<br>retrieval: Retrieves similar regions for reference."
S27	Comparative Pathology	GitHub	"Setup:<br>Clone the Repository: Clone the CPW repository from GitHub and navigate to the project directory.<br>Install Dependencies: Ensure you have Python and Django installed. Use the provided instructions in the repository to set up the environment and install necessary packages.<br>Configuration:<br>Server Setup: The platform can be deployed on a server. Follow the detailed configuration guide available in the repository, which includes setting up the Django environment and configuring WordPress integration if needed.<br>Data Integration: The CPW supports various datasets, including image and transcriptome data. Configure the system to access and analyze these datasets."
S28	Glomerulus Clustering	GitHub	Model Preparation:<br>Ensure you have the patch_model_he.hdf5 file in your working directory.<br>Set up a Python environment with TensorFlow and necessary dependencies.<br>Image Preparation:<br>Select H&E stained histopathology images for analysis.<br>If using whole slide images, prepare to extract 227x227 pixel patches.<br>Normalize the images to match the model's expected input.<br>Model Loading and Prediction:<br>Load the patch_model_he.hdf5 model using TensorFlow/Keras.<br>Feed prepared image patches into the model for prediction.<br>Result Interpretation:<br>Examine the model's output for each patch.<br>For whole slide images, reconstruct the full image prediction from individual patch results.<br>Post-processing and Visualization:<br>Apply any necessary thresholding or cleanup to the predictions.<br>Visualize the results, either as overlays on the original image or as separate output images.<br>Save or export the results as needed for further analysis
S29	Quip Distro	GitHub	Setup:<br>Requirements: Ensure Docker and Docker Compose are installed. Also, have Git and access to port 80 for web applications.<br>Clone the Repository: git clone https://github.com/SBU-BMI/quip_distro<br>Configuration: Navigate to the quip_distro directory. Copy the template configuration file and set up HTTPS if needed: cp config/httpd.conf.template config/httpd.conf<br>Building and Running:<br>Build the Docker containers using the command: docker-compose -f quip-pathdb.yml build<br>Start the services with: docker-compose -f quip-pathdb.yml up -d
S30	IHC Toolbox	NIH	"Installation:<br>Download and Install ImageJ:<br>Ensure you have ImageJ installed. You can download it from the ImageJ website.<br>Install the IHC Toolbox Plugin:<br>Download the IHC Toolbox plugin from the ImageJ plugin repository or a specified download link.<br>Place the downloaded .jar file into the plugins directory of your ImageJ installation.<br>Restart ImageJ. The plugin should now be available under the ""Plugins"" menu.<br>Usage:<br>Color Deconvolution:<br>Use the plugin to separate different staining components in the image, typically hematoxylin and diaminobenzidine (DAB).<br>Thresholding and Quantification:

			Apply thresholding to isolate stained areas and measure the area, intensity, or other parameters of interest."
S31	HistomicsML	GitHub	"Installation and Setup<br>Clone the Repository: git clone https://github.com/PathologyDataScience/HistomicsML.git<br>Install Dependencies: The repository includes a docker-compose.yml file for setting up the necessary environment using Docker. You can build and start the containers with: docker-compose up -d<br>For more detailed instructions and examples, please refer to the official documentation: https://histomicsml.readthedocs.io/en/latest/"
S32	Digital Pathology	GitHub	"Installation<br>Set Up Libraries and Machine:<br>Install Ruby 2.5.1 using RVM: sudo yum install -y curl gpg gcc gcc-c++ make<br>curl -sSL https://get.rvm.io | sudo bash -s stable<br>rvm install ruby-2.5.1<br>rvm --default use ruby-2.5.1<br>Install other necessary packages like Redis, Git, OpenSlide, and ImageMagick. For Ubuntu, use: sudo apt-get install -y redis git openslide-tools python3-openslide<br>Set Up PostgreSQL:<br>Install PostgreSQL and set up the imageviewer user:<br>sudo apt-get install postgresql postgresql-contrib libpq-dev<br>sudo systemctl start postgresql<br>sudo systemctl enable postgresql<br>Configure the database and set the appropriate user permissions.<br>Application Setup:<br>Clone the repository and set up the application:<br>git clone https://github.com/jkonglab/DigitalPathology<br>cd DigitalPathology<br>bundle install<br>Docker Deployment:<br>The application can also be deployed using Docker. The Dockerfile includes all necessary configurations. You can build and run the Docker image by navigating to the project directory and using: docker-compose up -d<br>Running the Application:<br>Start the application with Passenger or a similar server. Ensure environmental variables like DATABASE_USERNAME, DATABASE_PASSWORD, and SECRET_KEY_BASE are properly set."
S33	Run Onco	GitLab	"Requirements<br>Install Docker and Docker Compose: Ensure both are installed on your system.<br>Installation<br>Clone the Repository: git clone https://gitlab.com/peyrone/run-onco<br>Navigate to the Docker Directory: cd docker-and-compose<br>This directory contains the docker-compose.yml file.<br><br>Start the Docker Containers: source env.sh && docker-compose up --build -d<br>This command builds and starts the Docker containers in the background.<br><br>Optional: Stop the Docker Container: docker-compose down -v<br>Use this command to stop and remove the containers, including associated volumes.<br>Usage<br>Access the Web Application: Open a web browser and go to http://localhost:6002.<br>Default Login Information:<br>Username: icbbe<br>Password: demo2019"
S34	Scope2Screen	GitHub	Installation<br>Clone the Repository:<br>git clone https://github.com/labsyspharm/scope2screen.git<br>cd scope2screen<br>Docker Setup:<br>To run Scope2Screen as a Docker container, navigate to your data directory and execute: docker run --rm -v "$PWD":/data -p 8000:8000 labsyspharm/scope2screen:0.74<br>This command mounts the current directory as /data inside the container and exposes port 8000.<br>Conda Setup:<br>Install Conda and create a new environment: conda env create -f requirements.yml<br>Activate the Environment: conda activate scope2screen<br>Start the Server: python run.py<br>Access the application at http://localhost:8000/.<br>Executables: Precompiled executables for Windows and macOS are available for download, which can be run without additional installations
S35	OpenHI	GitLab	"Installation<br>Install Dependencies: Start by installing the necessary dependencies, including OpenSlide, libMI, MMSI, and Pyvips (for macOS and Windows). Use the following command to install Python dependencies: pip install -r requirements.txt<br>Database Setup:<br>Set up a MySQL database using the provided scripts in the module/legacy/static/ directory. Configure the database by copying and modifying the data_model_4-1_conf_example.ini file.<br>Initialize the database schema and fill it with data using: python data_model_4-1_fill_info.py<br>Install the OpenHI library with: python setup.py install<br>Usage<br>Configuration:<br>Configure the main settings in OpenHI_conf_example.ini, including database credentials and web server settings.<br>Run the Framework:<br>Start the framework by running the main application script:

			cd module/legacy/<br>python app.py<br>Access the application at http://127.0.0.1:5000."
S36	MMIR	GitHub	"Installation<br>Without Docker<br>Dependencies: Ensure you have MySQL and Python 3 installed.<br>Clone the Repository: git clone https://github.com/BMDSoftware/MMIR.git<br>cd MMIR<br>pip install -r requirements.txt<br>Database Setup:<br>Create a database in MySQL.<br>Set environment variables by executing the localVar.sh script (Linux) or manually setting them (Windows).<br>Migrate Database: python manage.py makemigrations<br>python manage.py migrate<br>Run the Server: python manage.py runserver<br>With Docker<br>Dependencies: Ensure Docker and Docker Compose are installed.<br>Build and Run:<br>docker-compose build<br>docker-compose up db<br>docker-compose up -d"
S37	FGCR	GitHub	"Installation<br>Clone the Repository: git clone https://github.com/hudingyi/FGCR.git<br>cd FGCR<br>Data Preparation<br>Dataset: The repository provides extracted patch features for the gastric dataset, which can be accessed from the provided Google Drive link. Ensure you have the data in the correct format and path.<br>Running the Model<br>Train and Test: Use the provided scripts (main.py, model.py) to train and evaluate the model. Make sure to adjust the configurations as needed for your specific data and computational setup."
S38	PathoRICH	GitHub	"Installation<br>Clone the Repository: git clone https://github.com/dmmoon/PathoRICH.git<br>Install Required Packages: pip install -r requirements.txt<br>Install Qupath: Download and install Qupath 0.3.0 on a Windows OS to visualize attention maps from the analysis.<br>Usage<br>Prepare Whole Slide Images (WSIs): Move the WSIs you want to analyze into the ""Input"" directory.<br>Run the Analysis: bash PathoRICH.sh<br>This script will generate patches, classify invasive cancer patches, extract features using a self-supervised CNN model, and analyze the WSIs at different magnifications (5x, 20x, and multiscale).<br>Output:<br>The analysis results, including probabilities of favorable and poor responses, are saved in the ""Result"" directory.<br>Attention maps and clustering results are also generated and can be viewed using Qupath.<br>Configuration: Modify config.py to adjust the number of clusters, figure size, and thresholds for cancer patch classification."
S39	Slideflow	GitHub	"Installation<br>git clone https://github.com/jamesdolezal/slideflow<br>cd slideflow<br>Set up the conda environment and install:<br>conda env create -f environment.yml<br>conda activate slideflow<br>python setup.py bdist_wheel<br>pip install dist/slideflow* cupy-cuda11x<br>Configuration and Usage<br>Backend Configuration: Set the backend (PyTorch or TensorFlow) using the SF_BACKEND environment variable.<br>Slide Reading: Specify the slide reading backend (cuCIM or Libvips) using SF_SLIDE_BACKEND.<br>Project Creation: Create projects to manage datasets, annotations, and extracted features. You can use preconfigured projects or set up custom ones with patient-level annotations.<br>Tile Extraction: Extract tiles from whole-slide images for analysis.<br>Training Models: Configure and train models with extracted tiles, supporting various learning methods like MIL, GANs, and SSL.<br>Visualization: Generate heatmaps, mosaic maps, and more for model interpretability and analysis."
S40	ComPRePS	GitHub	"Installation and Setup<br>1. Clone the Repository: git clone https://github.com/SarderLab/Multi-Compartment-Segmentation.git<br>Navigate to the multic/segmentationschool directory and create the necessary directories for data and output.<br>2. Model and Data Preparation:<br>Data Directory: Place your SVS images in the ./data directory.<br>Output Directory: Specify where the output should be saved in the ./output directory.<br>Model File: Update the slurm script with the path to the model file and the Singularity Image File (SIF).<br>3. Running the Segmentation:<br>Use the provided slurm script (slurm_predicting.sh) to perform the segmentation. Update the script with the correct paths to your data, output, and model files, then run it.<br>After setting up, you can use HistomicsUI in the Digital Slide Archive (DSA) to upload and visualize the results. The segmented compartments (such as cortical interstitium, medullary interstitium, non-sclerotic glomerulus, etc.) will be overlaid on the images, allowing for detailed analysis and visualization."
S41	Histo Cloud	GitHub	"Installation<br>1. Using Docker:<br>Clone the repository:

			git clone https://github.com/SarderLab/Histo-cloud.git<br>cd Histo-cloud<br>Build the Docker image: docker build --force-rm -t <username>/<docker imagename>:<tag> .<br>This Docker image can be imported into a running Digital Slide Archive (DSA) under ""Admin console / Plugins / Slicer CLI Web.""<br>2. Direct Python Installation:<br>For a direct installation, install the HistomicsTK package: pip install histomicstk<br>Follow additional steps provided in the repository for installing dependencies and setting up the environment."
S42	SSL CR Histo	GitHub	"Installation<br>1. Clone the Repository:<br>git clone https://github.com/srinidhiPY/SSL_CR_Histo.git<br>cd SSL_CR_Histo<br>2. Install Dependencies:<br>Make sure to have Python 3.7+ and install the required packages: pip install -r requirements.txt<br>Usage<br>1. Pretraining:<br>Use the scripts provided for different datasets (e.g., BreastPathQ, Camelyon16) to pretrain the model:<br>python pretrain_BreastPathQ.py<br>python pretrain_Camelyon16.py<br>These scripts will train a ResNet18 model using a self-supervised pretext task called Resolution Sequence Prediction (RSP).<br>2. Fine-tuning:<br>Fine-tune the pretrained model on specific tasks with limited labeled data:<br>python eval_BreastPathQ_SSL.py<br>python eval_Camelyon_SSL.py<br>python eval_Kather_SSL.py<br>3. Consistency Training:<br>Apply task-specific consistency training to refine the student network while keeping the teacher network frozen:<br>python eval_BreastPathQ_SSL_CR.py<br>python eval_Camelyon_SSL_CR.py<br>python eval_Kather_SSL_CR.py<br>Datasets<br>BreastPathQ: For tumor cellularity quantification.<br>Camelyon16: For breast tumor metastasis detection.<br>Kather dataset: For colorectal tissue-type classification."
S43	IHC Profiler	GitHub	"Install ImageJ: Ensure you have ImageJ installed on your system. You can download it from the ImageJ website.<br>IHC Profiler Installation<br>Download Plugin Files: Download the IHC_Profiler.class and IHC_Profiler.txt files from the repository.<br>Copy Files to ImageJ Directory:<br>Copy the IHC_Profiler.class file to the <ImageJ folder>/plugins directory.<br>Copy the IHC_Profiler.txt file to the <ImageJ folder>/macros directory.<br>Restart ImageJ: Restart ImageJ if it is already running. The ""IHC Profiler"" should now appear in the Plugins menu.<br>Once installed, you can use the plugin to analyze immunohistochemistry samples by creating a pixel-by-pixel analysis profile and assigning a score based on a four-tier system"
S44	AnnotatorJ	GitHub	"Installation<br>Prerequisites: Install JDK 1.8. Optionally, download ImageJ or Fiji.<br>Standalone: Download the pre-built version from the GitHub releases page. Extract the archive and run the annotator_Project-0.0.3-SNAPSHOT.jar file. No installation is needed, and it includes a basic ImageJ instance.<br>Fiji Update Site: Add the AnnotatorJ update site (https://sites.imagej.net/Spreka/) to your Fiji installation and update Fiji to install the plugin.<br>Usage<br>Starting the Plugin:<br>In Fiji, navigate to Plugins > AnnotatorJ to open the plugin.<br>Select the type of annotation: instance, semantic, or bounding box. Annotate objects and save your work in a sub-folder named after the selected class.<br>Additional Features:<br>Contour Assist: A trained U-Net model can assist in contouring. The model must be placed in the /plugins/models/ folder.<br>Exporting Annotations:<br>Use the AnnotatorJExporter plugin for exporting annotations in various formats."
S45	Style Transfer for Digital Pathology	GitHub	"Installation<br>Prerequisites: Ensure you have Miniconda installed with Python 3.<br>Create a Conda Environment: conda env create -f environment.yml<br>Activate the Environment: conda activate strap<br>Usage<br>1. Data Collection:<br>Prepare your dataset and download required datasets such as CRC-DX and Painter by Numbers from the provided sources.<br>2. repare Stylized Datasets:<br>Run the following command to create stylized datasets:<br>python create_stylized_dataset.py --content-path /path/to/content_images.hdf5 \\<br>--style-dir /path/to/style_images --out-path /path/to/save/stylized_dataset.hdf5 \\<br>--alpha 1.0 --content-size 1024 --style-size 256 --save-size 256<br>3. Train Models:<br>Train the models using different experiments (e.g., style_transfer, stain_augmentation):<br>python train.py --path2hdf5 /path/to/development-dataset.hdf5 \\<br>--save-dir /path/to/save/state-dicts --experiment 'style_transfer'<br>4. Evaluate Models:<br>Evaluate the trained models on the test dataset:

			python eval.py --data-dir /path/to/CRC-DX-TEST-dataset \ --state-dict-dir /path/to/state-dicts --experiment 'style_transfer'"
S46	CLAM	GitHub	"Installation<br>1. Clone the Repository:<br>git clone https://github.com/mahmoodlab/CLAM.git<br>cd CLAM<br>2. Set Up the Environment:<br>Install Miniconda and create an environment using the provided clam.yml file:<br>conda env create -f clam.yml<br>conda activate clam<br>Install other required packages with: pip install -r requirements.txt<br>3. Setup Pretrained Models:<br>Download pretrained models such as UNI and CONCH from Hugging Face and set the environment variables CONCH_CKPT_PATH and UNI_CKPT_PATH to their respective paths.<br>Usage<br>1. Feature Extraction:<br>Use the extract_features_fp.py script to extract features from whole slide images (WSIs). Set the --model_name to 'uni_v1' or 'conch_v1' depending on the model you wish to use.<br>2. Training:<br>Train the model with commands like: CUDA_VISIBLE_DEVICES=0 python main.py --task task_1_tumor_vs_normal --model_type clam_sb --data_root_dir DATA_ROOT_DIR<br>You can specify different tasks and models, such as clam_sb (single branch) or clam_mb (multi-branch), depending on your experimental setup.<br>3. Evaluation:<br>Evaluate the trained model on test data using: CUDA_VISIBLE_DEVICES=0 python eval.py --task task_1_tumor_vs_normal --model_type clam_sb --data_root_dir DATA_ROOT_DIR"
S47	CRANE	GitHub	"Installation:<br>Create a Conda Environment: conda env create -f env.yml<br>Activate the Environment: conda activate crane<br>Data Preparation:<br>Use the CLAM toolbox for tissue segmentation and feature extraction from biopsy slides. The extracted features are then used as inputs for the model."
S48	Cytomine	GitHub	"Installation<br>Clone the Repository:<br>git clone https://github.com/cytomine/Cytomine-Web-UI.git<br>cd Cytomine-Web-UI<br>Install Dependencies: npm install<br>Configuration:<br>Modify src/utils/constants.js to set the values for CYTOMINE_CORE_HOST and CYTOMINE_UPLOAD_HOST according to your Cytomine instance.<br>Build and Serve:<br>To build the front-end files, run: npm run build<br>For local development, you can start a server with: npm run serve<br>The application will be available at http://localhost:8080."
S49	Ilastik	GitHub	"Installation<br>1. Binary Installation:<br>Visit the ilastik download page and download the latest version suitable for your operating system. Follow the provided installation instructions for a straightforward setup.<br>2. Conda Installation (Experimental):<br>You can also install ilastik using conda:<br>mamba create -n ilastik --override-channels -c pytorch -c ilastik-forge -c conda-forge ilastik<br>conda activate ilastik<br>ilastik<br>This method supports various Python versions, including 3.7 to 3.9."
S50	HistoColAi	GitHub	"Installation on Windows<br>Install XAMPP:<br>Download and install XAMPP for Windows from the official source.<br>Set Up Project:<br>Create a new folder named histocolai in the xampp/htdocs directory.<br>Download the repository files and place them in the histocolai folder.<br>Initialize phpMyAdmin from the XAMPP console and create a new database named histocolai.<br>Import the data from histocolai.sql located in the DataBase folder of the repository.<br>Download additional resources from the provided SharePoint link and unzip them into the images directory within histocolai.<br>Run the Application:<br>Start the Apache server from the XAMPP control panel.<br>Access the application via http://localhost/histocolai/histocolai.html?source=images/batch_spitzoid_proof/dzi_images.json and log in with the credentials user: test2, password: test2"
S51	CellProfiler	GitHub	"Installation<br>Download: Obtain the stable release for macOS or Windows from the CellProfiler website.<br>Compile from Source: For those contributing to CellProfiler or needing specific configurations, follow the compilation instructions available on GitHub.<br>Usage<br>Interface: CellProfiler provides a graphical user interface for creating pipelines to analyze biological images. It supports various modules for image processing, object identification, and feature extraction.<br>Integration

			Plugins and Add-ons: The community-contributed CellProfiler plugins extend its capabilities, offering additional modules for specialized tasks."
S52	ICY	ICY	"Download and Install:<br>Visit the Icy website and navigate to the download section.<br>Choose the version compatible with your operating system (Windows, macOS, or Linux). Installation instructions are available on the site.<br>Features:<br>Image Analysis: Icy offers tools for visualization, annotation, and quantification of bioimaging data. It includes advanced features like 3D visualization, batch processing, and image registration.<br>Plugin Support: Users can extend functionality by adding plugins, which can be installed directly from the Icy interface.<br>Programming and Scripting: Icy supports Java-based plugin development and scripting in Python, offering flexibility for custom analyses.<br>Usage:<br>Once installed, you can start Icy and load your images for analysis. Utilize the available plugins and protocols to perform complex image processing tasks."

Supplementary Table 11: Comparison of Open-Source Bioimage Analysis Tools

Feature Category	QuPath	Ilastik	CellProfiler	ICY	Cytomine
Core Functionalities					
Whole Slide Imaging Support	Yes	Limited	Limited	Limited	Yes
Multi-channel Support	Yes	Yes	Yes	Yes	Yes
3D/Time-lapse Support	Limited	Yes	Limited	Yes	Limited
Batch Processing	Yes	Yes	Yes	Yes	Yes
Machine Learning Integration	Yes	Yes	Yes	Yes	Yes
Performance Metrics					
Large Image Processing Speed*	Fast	Medium	Medium	Medium	Fast
Memory Usage Efficiency**	High	Medium	Medium	Medium	High
Maximum Image Size Handled	>40GB	4GB	4GB	4GB	>40GB
Technical Requirements					
Programming Knowledge Needed	Optional	No	No	No	Optional
System Requirements	Medium	Medium	Medium	Medium	High
Cloud/Server Deployment	No	No	No	No	Yes
User Features					
GUI Interface	Yes	Yes	Yes	Yes	Yes
Scripting/Plugin Support	Yes	Yes	Yes	Yes	Yes
Collaborative Features	Limited	No	No	Limited	Yes
Application Areas					
Digital Pathology	Strong	Limited	Limited	Limited	Strong
Cell Biology	Good	Strong	Strong	Strong	Good
High-Content Screening	Good	Good	Strong	Good	Good

Notes:

Speed ratings based on processing 1GB whole slide images ** Memory efficiency based on handling 40GB whole slide images

Supplementary Table 12: Summary of Core Features, Supported Modalities, and Limitations of All Included Studies

ID	Tool Name	Category	Core Features	Supported Modalities	Limitations
S1	CS-CO	Framework	Hybrid self-supervised learning combining generative (cross-stain prediction) and contrastive methods	WSIs (H&E)	Computationally intensive; limited to H&E; limited user studies
S2	WSI-GTFE	Tool	Integrates GNNs and TDA to summarize patch-level CNN embeddings for cancer staging	WSIs	Scalability concerns; limited external validation
S3	Minerva Story	Tool	Interactive narrative browser with multi-omic and tissue atlas integration	Multiplexed Tissue Imaging, WSIs	Dependent on annotation quality; relies on user narratives
S4	Depicter	Tool	Web-based annotation tool using self-supervised clustering and iterative refinement	WSIs	High computational requirements; performance variability
S5	Webmicroscope	Tool	Virtual microscopy platform for Gleason grading; facilitates digital slide viewing	WSIs, Digitized Histological Sections	Limited standardization; moderate interobserver agreement
S6	HistoStitcher	Tool	GUI-based tool for reassembling fragmented histological images via image transformation and stitching	WSIs	May require substantial resources for high-res images
S7	Digital Slide Archive	Software	Scalable image management with integration of HistomicsTK; supports annotations and image analysis	WSIs	Limited security for clinical use; designed for deidentified data
S8	Visinity	Tool	Visual analytics system for spatial cell interaction analysis with interactive querying	Multiplexed Tissue Imaging	Dependent on single-cell data; further usability studies needed
S9	Orbit Image Analysis	Software	Framework for objective WSI quantification using tile-based deep learning segmentation	WSIs	High computational demands; quality depends on training data
S10	QuPath	Software	Comprehensive bioimage analysis platform with robust annotation, segmentation, and extendable plugins	WSIs, IHC	High resource demand; performance heavily hardware-dependent
S11	FAST Pathology	Software	Deep learning–based WSI analysis with GPU-based rendering and multiple inference engines	WSIs	Hardware dependent; limited advanced annotation features
S12	DMMN-ovary	Tool	Deep learning model for ovarian cancer segmentation using pretrained models and interactive refinement	WSIs	Suboptimal BRCA mutation prediction; further validation required
S13	PanCancer	Tool	Multimodal prognostic model integrating WSIs with molecular profiles for risk stratification	WSIs	High computational demands; limited external validation
S14	SynCLay	Framework	Generates realistic synthetic histology images using GANs, HoVer-Net, and graph CNNs	WSIs	Relies on user-defined parameters; minor imperfections in synthetic outputs
S15	Desuto Platform	Tool	Content-based visual search system for WSIs integrating visual and text features	WSIs	Requires manual annotation; limited to specific histopathology types
S16	SC-Net	Framework	Weakly-supervised nuclei segmentation using co-training and colorization networks	WSIs	High computation; depends on complete point annotations; further usability evaluation needed
S17	DigiPathAI	Tool	Interactive ML system for histologic object classification with active learning	WSIs	Requires specialized expertise; potential bias from limited data heterogeneity
S18	Xopat	Tool	Web-based WSI viewer with annotation tools built on Node.js and Docker	WSIs	Requires proper configuration; dependent on web infrastructure
S19	NuClick	Framework	Encoder-decoder network for interactive segmentation of nuclei, cells, and glands with minimal user input	WSIs	Limited usability evaluation; clinical validation limited
S20	HistoQC	Software	Automated quality control tool for digital slides using image metrics and supervised classifiers	WSIs	Limited to H&E bright-field; high computational requirements; limited usability evaluation
S21	DeGPRR	Framework	Enhances cell detection by integrating expert knowledge with deep learning features	WSIs	Relies on expert input; computationally intensive; limited user experience evaluation
S22	SLIM	Tool	Web-based slide microscopy viewer supporting DICOM standards with GPU-accelerated rendering	WSIs, Fluorescence	Requires conversion of non-DICOM formats; dependent on DICOMweb services
S23	Omni-Seg	Tool	Dynamic multi-tissue segmentation with scale- and class-aware controllers using semi-supervised learning	WSIs	Limited to renal pathology; not designed for interactive visual analytics
S24	FlexTileSource	Tool	Optimized tile source for OpenSeadragon that accelerates field-of-view rendering for WSIs	WSIs	Performance gain varies with native image pyramid structure
S25	Cellular Composition	Tool	System for cell classification and patch-level analysis integrated with QuPath for feature aggregation	WSIs	Limited to ovarian tumors; requires manual annotation; does not cover all histologic features
S26	Thyroid Interactive	Tool	Interactive system for thyroid WSI classification using deep neural network–based feature fusion	WSIs	Focused on thyroid; limited training samples; lacks extensive usability evaluation

S27	Comparative Pathology	Tool	Collaborative platform for comparing histopathology images with integrated external resources and annotations	WSIs	Requires internet; slow rendering for large workbenches; not optimized for mobile devices
S28	Glomerulus Clustering	Framework	Unsupervised clustering using NASNet, UMAP, and Gaussian Mixture Models for kidney biopsy assessment	WSIs, Microscopy Images	Short observation period; subjective clustering; low R^2 in regression models
S29	Quip Distro	Tool	Containerized software system for managing and analyzing WSI features using Docker	WSIs	High computational needs; limited to specific cancer types; extensive validation needed
S30	IHC Toolbox	Tool	ImageJ plugin for automated color deconvolution and quantification of IHC images using pixel profiling	IHC, WSIs	Limited to specific stains; requires manual selection; potential interobserver variability
S31	HistomicsML	Software	Interactive web application for training classifiers on WSIs using active learning and minimal user input	WSIs, IHC	Scalability limited by memory; relies on external segmentation; inter-reader variation not fully addressed
S32	Digital Pathology	Framework	Web-based platform supporting multi-resolution viewing, 3D reconstruction, and image registration	WSIs, Multimodal Images	Full functionality only in certain browsers (e.g., Chrome)
S33	Run Onco	Tool	Platform for managing cancer precision medicine data with integration of clinical and omics data	WSIs	Requires further development for complex analyses; handling extremely large datasets is challenging
S34	Scope2Screen	Tool	Web-based application for interactive exploration and annotation of multi-channel tissue images with linked multivariate data	WSIs, Multiplexed Tissue Imaging	Does not support batch annotation; limited single-cell marker analysis
S35	OpenHI	Software	Collaborative platform for precise WSI annotation with multi-level pre-segmentation and a virtual magnification indicator	WSIs	Lacks consolidation of multi-expert annotations; processing time increases with image area; limited usability
S36	MMIR	Tool	Web application for multimodal histological image registration using ORB, SIFT, FLANN, and KNN	WSIs, Multimodal Images	Performance varies with image types; complexity in handling diverse images
S37	FGCR	Framework	Anchor-based attention and prompt-based text representation for cross-modal retrieval of WSIs and diagnostic reports	WSIs	Dependent on accurate diagnostic reports; limited to specific histopathology types
S38	PathoRICH	Tool	Multiple instance learning framework with spatial transformer networks for predicting treatment responses in ovarian cancer	WSIs	Suboptimal external validation; requires multicenter validation; limited mutation prediction performance
S39	Slideflow	Software	Digital pathology library supporting multiple deep learning backends and real-time visualization with interpretability features	WSIs, IHC, Microscopy Images	Requires specific deep learning backend; limited support for graph neural networks; planned future updates
S40	ComPRePS	Tool	Automated computational renal pathology suite using Detectron2 for panoptic segmentation and FTU analysis	WSIs	Limited to kidney pathology; high computational resources; limited usability evaluation
S41	Histo Cloud	Tool	Cloud-based tool for WSI segmentation integrating DeepLab V3+ with a user-friendly interface	WSIs	High computational requirements; limited to specific tissue types; limited user studies
S42	SSL CR Histo	Framework	Self-supervised pretext task with teacher-student consistency training for limited-label analysis	WSIs	Limited to specific tasks; dependent on data augmentation; requires large datasets for training
S43	IHC Profiler	Tool	ImageJ plugin for automated IHC scoring using color deconvolution and pixel profiling	IHC, Microscopy Images	May require expert supervision; not compatible with certain immunomarkers
S44	AnnotatorJ	Tool	ImageJ plugin for semi-automatic annotation with contour assist via U-Net presegmentation	Microscopy Images, IHC	Requires manual refinement; steep learning curve for new users
S45	Style Transfer for Digital Pathology	Framework	AdaIN-based style transfer framework for domain-agnostic visual representation and stain augmentation	WSIs	Computationally expensive; requires large datasets for training
S46	CLAM	Tool	Attention-based pooling and clustering-constrained MIL for weakly-supervised WSI analysis	WSIs	Requires large datasets for training; computationally expensive
S47	CRANE	Tool	Attention-based pooling and clustering-constrained MIL for diagnostic analysis of WSIs	WSIs	Requires large datasets for training; computationally expensive
S48	Cytomine	Software	Collaborative web-based platform for multi-gigapixel image analysis with integrated ML tools	WSIs, IHC	Dependent on network stability; potential latency; requires robust server infrastructure
S49	Ilastik	Software	Interactive ML tool for bioimage analysis using supervised algorithms (e.g., Random Forest) for pixel/object classification	WSIs, IHC, Microscopy Images	Scalability limited for extremely large WSIs; relies on predefined workflows; limited flexibility
S50	HistoColAi	Software	Web platform for digital histology annotation and analysis with CNNs for tumor region identification and quantification	WSIs	Limited to specific neoplasms; high computational resource demands; complex initial setup for inexperienced users
S51	CellProfiler	Software	High-throughput, modular software for quantitative cell image analysis and feature measurement	Microscopy Images	Limited interactive visualization; not suited for real-time analysis; computationally intensive for large datasets

S52	ICY	Software	Collaborative bioimage informatics platform with advanced 3D visualization, batch processing, and plugin/scripting support	WSIs, IHC, Microscopy Images, Multiplexed	Steep learning curve; complex initial setup; relies on community contributions for extensibility

References

[S1] Yang P, Yin X, Lu H, Hu Z, Zhang X, Jiang R, et al. CS-CO: A Hybrid Self-Supervised Visual Representation Learning Method for H&E-stained Histopathological Images. Med Image Anal 2022;81:102539. https://doi.org/https://doi.org/10.1016/j.media.2022.102539.

[S2] Levy J, Haudenschild C, Barwick C, Christensen B, Vaickus L. Topological Feature Extraction and Visualization of Whole Slide Images using Graph Neural Networks. Biocomput. 2021, World Scientific; 2021, p. 285–96. https://doi.org/10.1142/9789811232701_0027.

[S3] Rashid R, Chen Y-A, Hoffer J, Muhlich JL, Lin J-R, Krueger R, et al. Narrative online guides for the interpretation of digital-pathology images and tissue-atlas data. Nat Biomed Eng 2022;6:515–26. https://doi.org/10.1038/s41551-021-00789-8.

[S4] Chelebian E, Avenel C, Ciompi F, Wählby C. DEPICTER: Deep representation clustering for histology annotation. Comput Biol Med 2024;170:108026. https://doi.org/https://doi.org/10.1016/j.compbiomed.2024.108026.

[S5] Helin H, Lundin M, Lundin J, Martikainen P, Tammela T, Helin H, et al. Web-based virtual microscopy in teaching and standardizing Gleason grading. Hum Pathol 2005;36:381–6. https://doi.org/https://doi.org/10.1016/j.humpath.2005.01.020.

[S6] Chappelow J, Tomaszewski JE, Feldman M, Shih N, Madabhushi A. HistoStitcher©: An interactive program for accurate and rapid reconstruction of digitized whole histological sections from tissue fragments. Comput Med Imaging Graph 2011;35:557–67. https://doi.org/https://doi.org/10.1016/j.compmedimag.2011.01.010.

[S7] Gutman DA, Khalilia M, Lee S, Nalisnik M, Mullen Z, Beezley J, et al. The Digital Slide Archive: A Software Platform for Management, Integration, and Analysis of Histology for Cancer Research. Cancer Res 2017;77:e75–8. https://doi.org/10.1158/0008-5472.CAN-17-0629.

[S8] Warchol S, Krueger R, Nirmal AJ, Gaglia G, Jessup J, Ritch CC, et al. Visinity: Visual Spatial Neighborhood Analysis for Multiplexed Tissue Imaging Data. IEEE Trans Vis Comput Graph 2023;29:106–16. https://doi.org/10.1109/TVCG.2022.3209378.

[S9] Stritt M, Stalder AK, Vezzali E. Orbit Image Analysis: An open-source whole slide image analysis tool. PLOS Comput Biol 2020;16:1–19. https://doi.org/10.1371/journal.pcbi.1007313.

[S10] Bankhead P, Loughrey MB, Fernández JA, Dombrowski Y, McArt DG, Dunne PD, et al. QuPath: Open source software for digital pathology image analysis. Sci Rep 2017;7:16878. https://doi.org/10.1038/s41598-017-17204-5.

[S11] Pedersen A, Valla M, Bofin AM, De Frutos JP, Reinertsen I, Smistad E. FastPathology: An Open-Source Platform for Deep Learning-Based Research and Decision Support in Digital Pathology. IEEE Access 2021;9:58216–29. https://doi.org/10.1109/ACCESS.2021.3072231.

[S12] Ho DJ, Chui MH, Vanderbilt CM, Jung J, Robson ME, Park C-S, et al. Deep Interactive Learning-based ovarian cancer segmentation of H&E-stained whole slide images to study morphological patterns of BRCA mutation. J Pathol Inform 2023;14:100160. https://doi.org/https://doi.org/10.1016/j.jpi.2022.100160.

[S13] Chen RJ, Lu MY, Williamson DFK, Chen TY, Lipkova J, Noor Z, et al. Pan-cancer integrative histology-genomic analysis via multimodal deep learning. Cancer Cell 2022;40:865-878.e6. https://doi.org/10.1016/j.ccell.2022.07.004.

[S14] Deshpande S, Dawood M, Minhas F, Rajpoot N. SynCLay: Interactive synthesis of histology images from bespoke cellular layouts. Med Image Anal 2024;91:102995. https://doi.org/https://doi.org/10.1016/j.media.2023.102995.

[S15] Schaer R, Otálora S, Jimenez-del-Toro O, Atzori M, Müller H. Deep Learning-Based Retrieval System for Gigapixel Histopathology Cases and the Open Access Literature. J Pathol Inform 2019;10:19. https://doi.org/https://doi.org/10.4103/jpi.jpi_88_18.

[S16] Lin Y, Qu Z, Chen H, Gao Z, Li Y, Xia L, et al. Nuclei segmentation with point annotations from pathology images via self-supervised learning and co-training. Med Image Anal 2023;89:102933. https://doi.org/https://doi.org/10.1016/j.media.2023.102933.

[S17] Khened M, Kori A, Rajkumar H, Krishnamurthi G, Srinivasan B. A generalized deep learning framework for whole-slide image segmentation and analysis. Sci Rep 2021;11:11579. https://doi.org/10.1038/s41598-021-90444-8.

[S18] Archambault D, Bujack R, Schreck T, Horák J, Furmanová K, Kozlíková B, et al. xOpat: eXplainable Open Pathology Analysis Tool. Comput Graph Forum 2023;42:63–73. https://doi.org/10.1111/cgf.14812.

[S19] Alemi Koohbanani N, Jahanifar M, Zamani Tajadin N, Rajpoot N. NuClick: A deep learning framework for interactive segmentation of microscopic images. Med Image Anal 2020;65:101771. https://doi.org/https://doi.org/10.1016/j.media.2020.101771.

[S20] Janowczyk A, Zuo R, Gilmore H, Feldman M, Madabhushi A. HistoQC: An Open-Source Quality Control Tool for Digital Pathology Slides. JCO Clin Cancer Informatics 2019:1–7. https://doi.org/10.1200/CCI.18.00157.

[S21] Tyagi AK, Mohapatra C, Das P, Makharia G, Mehra L, AP P, et al. DeGPR: Deep Guided Posterior Regularization for Multi-Class Cell Detection and Counting. 2023 IEEE/CVF Conf. Comput. Vis. Pattern Recognit., 2023, p. 23913–23. https://doi.org/10.1109/CVPR52729.2023.02290.

[S22] Gorman C, Punzo D, Octaviano I, Pieper S, Longabaugh WJR, Clunie DA, et al. Interoperable slide microscopy viewer and annotation tool for imaging data science and computational pathology. Nat Commun 2023;14:1572. https://doi.org/10.1038/s41467-023-37224-2.

[S23] Deng R, Liu Q, Cui C, Yao T, Long J, Asad Z, et al. Omni-Seg: A Scale-Aware Dynamic Network for Renal Pathological Image Segmentation. IEEE Trans Biomed Eng 2023;70:2636–44. https://doi.org/10.1109/TBME.2023.3260739.

[S24] Schüffler PJ, Ozcan GG, Al-Ahmadie H, Fuchs TJ. FlexTileSource: An OpenSeadragon Extension for Efficient Whole-Slide Image Visualization. J Pathol Inform 2021;12:31. https://doi.org/https://doi.org/10.4103/jpi.jpi_13_21.

[S25] Jiang J, Tekin B, Guo R, Liu H, Huang Y, Wang C. Digital Pathology-based Study of Cell- and Tissue-level Morphologic Features in Serous Borderline Ovarian Tumor and High-grade Serous Ovarian Cancer. J Pathol Inform 2021;12:24. https://doi.org/https://doi.org/10.4103/jpi.jpi_76_20.

[S26] Chen P, Shi X, Liang Y, Li Y, Yang L, Gader PD. Interactive thyroid whole slide image diagnostic system using deep representation. Comput Methods Programs Biomed 2020;195:105630. https://doi.org/https://doi.org/10.1016/j.cmpb.2020.105630.

[S27] Wicks MN, Glinka M, Hill B, Houghton D, Sharghi M, Ferreira I, et al. The Comparative Pathology Workbench: Interactive visual analytics for biomedical data. J Pathol Inform 2023;14:100328. https://doi.org/https://doi.org/10.1016/j.jpi.2023.100328.

[S28] Sato N, Uchino E, Kojima R, Sakuragi M, Hiragi S, Minamiguchi S, et al. Evaluation of Kidney Histological Images Using Unsupervised Deep Learning. Kidney Int Reports 2021;6:2445–54. https://doi.org/https://doi.org/10.1016/j.ekir.2021.06.008.

[S29] Saltz J, Sharma A, Iyer G, Bremer E, Wang F, Jasniewski A, et al. A Containerized Software System for Generation, Management, and Exploration of Features from Whole Slide Tissue Images. Cancer Res 2017;77:e79–82. https://doi.org/10.1158/0008-5472.CAN-17-0316.

[S30] Shu J, Dolman GE, Duan J, Qiu G, Ilyas M. Statistical colour models: an automated digital image analysis method for quantification of histological biomarkers. Biomed Eng Online 2016;15:46. https://doi.org/10.1186/s12938-016-0161-6.

[S31] Nalisnik M, Amgad M, Lee S, Halani SH, Velazquez Vega JE, Brat DJ, et al. Interactive phenotyping of large-scale histology imaging data with HistomicsML. Sci Rep 2017;7:14588. https://doi.org/10.1038/s41598-017-15092-3.

[S32] Shen A, Wang F, Paul S, Bhuvanapalli D, Alayof J, Farris AB, et al. An integrative web-based software tool for multi-dimensional pathology whole-slide image analytics. Phys Med Biol 2022;67:224001. https://doi.org/10.1088/1361-6560/ac8fde.

[S33] Peyrone N, Wichadakul D. RUN-ONCO: A Highly Extensible Software Platform for Cancer Precision Medicine. Proc. 2019 6th Int. Conf. Biomed. Bioinforma. Eng., New York, NY, USA: Association for Computing Machinery; 2020, p. 142–147. https://doi.org/10.1145/3375923.3375928.

[S34] Jessup J, Krueger R, Warchol S, Hoffer J, Muhlich J, Ritch CC, et al. Scope2Screen: Focus+Context Techniques for Pathology Tumor Assessment in Multivariate Image Data. IEEE Trans Vis Comput Graph 2022;28:259–69. https://doi.org/10.1109/TVCG.2021.3114786.

[S35] Puttapirat P, Zhang H, Deng J, Dong Y, Shi J, Lou P, et al. OpenHI: open platform for histopathological image annotation. Int J Data Min Bioinform 2019;22:328–49. https://doi.org/10.1504/IJDMB.2019.101393.

[S36] Escobar Díaz Guerrero R, Oliveira JL, Popp J, Bocklitz T. MMIR: an open-source software for the registration of multimodal histological images. BMC Med Inform Decis Mak 2024;24:65. https://doi.org/10.1186/s12911-024-02424-3.

[S37] Hu D, Jiang Z, Shi J, Xie F, Wu K, Tang K, et al. Histopathology language-image representation learning for fine-grained digital pathology cross-modal retrieval. Med Image Anal 2024;95:103163. https://doi.org/https://doi.org/10.1016/j.media.2024.103163.

[S38] Ahn B, Moon D, Kim H-S, Lee C, Cho NH, Choi H-K, et al. Histopathologic image--based deep learning classifier for predicting platinum-based treatment responses in high-grade serous ovarian cancer. Nat Commun 2024;15:4253. https://doi.org/10.1038/s41467-024-48667-6.

[S39] Dolezal JM, Kochanny S, Dyer E, Ramesh S, Srisuwananukorn A, Sacco M, et al. Slideflow: deep learning for digital histopathology with real-time whole-slide visualization. BMC Bioinformatics 2024;25:134. https://doi.org/10.1186/s12859-024-05758-x.

[S40] Mimar S, Paul AS, Lucarelli N, Border S, Naglah A, Barisoni L, et al. ComPRePS: an automated cloud-based image analysis tool to democratize AI in digital pathology. In: Tomaszewski JE, Ward AD, editors. Med. Imaging 2024 Digit. Comput. Pathol., vol. 12933, SPIE; 2024, p. 129330Z. https://doi.org/10.1117/12.3008469.

[S41] Lutnick B, Manthey D, Becker JU, Ginley B, Moos K, Zuckerman JE, et al. A user-friendly tool for cloud-based whole slide image segmentation with examples from renal histopathology. Commun Med 2022;2:105. https://doi.org/10.1038/s43856-022-00138-z.

[S42] Srinidhi CL, Kim SW, Chen F-D, Martel AL. Self-supervised driven consistency training for annotation efficient histopathology image analysis. Med Image Anal 2022;75:102256. https://doi.org/https://doi.org/10.1016/j.media.2021.102256.

[S43] Varghese F, Bukhari AB, Malhotra R, De A. IHC Profiler: An Open Source Plugin for the Quantitative Evaluation and Automated Scoring of Immunohistochemistry Images of Human Tissue Samples. PLoS One 2014;9:1–11. https://doi.org/10.1371/journal.pone.0096801.

[S44] Hollandi R, Diósdi Á, Hollandi G, Moshkov N, Horváth P. AnnotatorJ: an ImageJ plugin to ease hand annotation of cellular compartments. Mol Biol Cell 2020;31:2179–86. https://doi.org/10.1091/mbc.E20-02-0156.

[S45] Yamashita R, Long J, Banda S, Shen J, Rubin DL. Learning Domain-Agnostic Visual Representation for Computational Pathology Using Medically-Irrelevant Style Transfer Augmentation. IEEE Trans Med Imaging 2021;40:3945–54. https://doi.org/10.1109/TMI.2021.3101985.

[S46] Lu MY, Williamson DFK, Chen TY, Chen RJ, Barbieri M, Mahmood F. Data-efficient and weakly supervised computational pathology on whole-slide images. Nat Biomed Eng 2021;5:555–70. https://doi.org/10.1038/s41551-020-00682-w.

[S47] Lipkova J, Chen TY, Lu MY, Chen RJ, Shady M, Williams M, et al. Deep learning-enabled assessment of cardiac allograft rejection from endomyocardial biopsies. Nat Med 2022;28:575–82. https://doi.org/10.1038/s41591-022-01709-2.

[S48] Marée R, Rollus L, Stévens B, Hoyoux R, Louppe G, Vandaele R, et al. Collaborative analysis of multi-gigapixel imaging data using Cytomine. Bioinformatics 2016;32:1395–401. https://doi.org/10.1093/bioinformatics/btw013.

[S49] Berg S, Kutra D, Kroeger T, Straehle CN, Kausler BX, Haubold C, et al. ilastik: interactive machine learning for (bio)image analysis. Nat Methods 2019;16:1226–32. https://doi.org/10.1038/s41592-019-0582-9.

[S50] Pulgarín-Ospina CC, del Amor R, Colomera A, Silva-Rodríguez J, Naranjo V. HistoColAi: An Open-Source Web Platform for Collaborative Digital Histology Image Annotation with AI-Driven Predictive Integration 2023. https://doi.org/https://arxiv.org/abs/2307.07525.

[S51] Carpenter AE, Jones TR, Lamprecht MR, Clarke C, Kang IH, Friman O, et al. CellProfiler: image analysis software for identifying and quantifying cell phenotypes. Genome Biol 2006;7:R100. https://doi.org/10.1186/gb-2006-7-10-r100.

[S52] de Chaumont F, Dallongeville S, Chenouard N, Hervé N, Pop S, Provoost T, et al. Icy: an open bioimage informatics platform for extended reproducible research. Nat Methods 2012;9:690–6. https://doi.org/10.1038/nmeth.2075.
